# An insight into the sialome of *Simulium guianense *(DIPTERA:SIMulIIDAE), the main vector of River Blindness Disease in Brazil

**DOI:** 10.1186/1471-2164-12-612

**Published:** 2011-12-19

**Authors:** Andrezza C Chagas, Eric Calvo, Paulo FP Pimenta, José MC Ribeiro

**Affiliations:** 1 Laboratory of Malaria and Vector Research, National Institute of Allergy and Infectious Diseases, 12735 Twinbrook Parkway, National Institutes of Health, Rockville, Maryland 20892-8132, USA; 2 Entomology Laboratory, Centro de Pesquisa René Rachou, Belo Horizonte, Minas Gerais, Brazil

## Abstract

**Background:**

Little is known about the composition and function of the saliva in black flies such as *Simulium guianense*, the main vector of river blindness disease in Brazil. The complex salivary potion of hematophagous arthropods counteracts their host's hemostasis, inflammation, and immunity.

**Results:**

Transcriptome analysis revealed ubiquitous salivary protein families--such as the Antigen-5, Yellow, Kunitz domain, and serine proteases--in the *S. guianense *sialotranscriptome. Insect-specific families were also found. About 63.4% of all secreted products revealed protein families found only in *Simulium*. Additionally, we found a novel peptide similar to kunitoxin with a structure distantly related to serine protease inhibitors. This study revealed a relative increase of transcripts of the SVEP protein family when compared with *Simulium vittatum *and *S. nigrimanum *sialotranscriptomes. We were able to extract coding sequences from 164 proteins associated with blood and sugar feeding, the majority of which were confirmed by proteome analysis.

**Conclusions:**

Our results contribute to understanding the role of *Simulium *saliva in transmission of *Onchocerca volvulus *and evolution of salivary proteins in black flies. It also consists of a platform for mining novel anti-hemostatic compounds, vaccine candidates against filariasis, and immuno-epidemiologic markers of vector exposure.

## Background

Onchocerciasis (river blindness) is a disease caused by *Onchocerca volvulus*, a filarial worm transmitted by the bite of black flies. Onchocerciasis ranks fourth among the causes of blindness and visual impairment in developing countries [1]. InBrazil, about 1.8% of the population needs treatment, all of whom reside in a vast single focus (Amazonas-Roraima focus), bordering Venezuela [2].

*Simulium guianense *appears to be the main vector in this focus [3,4], but its biology is not well studied. Saliva of hematophagous arthropods contains a vast array of pharmacologically active compounds that act as anticlotting, antiplatelet, vasodilatory, anti-inflammatory, and immunomodulatory compounds. Some functional and biochemical characterizations have been previously obtained with salivary gland homogenates (SGHs) of *Simulium vittatum*, such as anti-fXa [5,6], antithrombin [7], apyrase [8], hyaluronidase [9], vasodilators [10] and immunomodulators [11-13].

Recently, sialotranscriptomes (from the Greek Sialo = saliva) of Nearctic and Neotropical black flies have revealed transcripts related to the functions previously described [14,15]. Analysis of salivary transcriptomes of bloodfeeding arthropods--including different genera of mosquitoes [16-26], sand flies [27-31], biting midges [32-34], black flies [14,15], ticks [35-45], bed bugs [46], triatomines [47-50], tse tse flies [51] and fleas [52]--have found a great diversity of protein families in different arthropods and suggested a fast evolution of several of these salivary protein families, possibly resulting from their host's immune pressure. Thus, because of this great diversity, many salivary proteins do not show sequence similarities to other known proteins. This also reflects the independent evolution of blood feeding within insects, which occurred approximately 30 times within this group [53].

Evidence suggests that the infraorder Culicomorpha originated from a single bloodfeeding ancestor during the Triassic, over 200 million years ago (MYA), with some families having lost this lifestyle [53]. Black flies appear as more basal of the Culicomorpha with regard to the medical importance in this clade. In contrast, the infraorder Psychodomorpha, which includes sand flies, probably had a very early origin and isolated phylogenetic position [53]. Data suggest that, alternatively, the blood feeding behavior could have evolved independently in each family of the Nematocera [54-56].

The Simuliidae family contains 2,025 named species, 12 of which are fossil, and is widely distributed to all biogeographic regions [57]. Their meal source is based on warm-blooded animals including man, cattle, and birds, but also reptiles [58]. In this work, we compare the sialotranscriptome of female *S. guianense *with those of other black flies available in the non-redundant (NR) protein database of the National Center for Biotechnology Information (NCBI, National Library of Medicine, NIH) database: *Simulium vittatum *(Neartic, autogenous, and zoophilic) and *Simulium nigrimanum *(Neotropical, anautogenous, and anthropophilic, but also zoophilic).

We present the analysis of a set 1,722 cDNA sequences out of 1,974 that yielded good sequence quality, 74.7% of which were associated with secreted products. We describe 174 coding sequences--mostly full length--the majority of which were confirmed by tryptic digestion/mass spectrometry (MS). Most salivary proteins found have no known function. Our results should help to understand the molecular evolution of black flies to blood feeding, characterize the role of some protein families associated with sugar feeding, and contribute to our understanding of the role of the *Simulium *saliva in the transmission of *O. volvulus*. It also consists of a platform for mining novel antihemostatic compounds and vaccine candidates against filariasis.

## Results and discussion

### cDNA Library Characteristics

A total of 1,772 clones out of 1,974 that were sequenced yielded good quality sequences and were used to assemble a database that yielded 752 clusters of related sequences, 491 of which contained only one EST. The consensus sequence of each cluster is named either a contig (deriving from two or more sequences) or a singleton (deriving from a single sequence). As indicated before [14,15], this paper uses "cluster" or "contig" to denote sequences derived from both consensus sequences and singletons. The 752 clusters were compared using the program blastx, blastn, or rpsblast [59] to the NR protein database of the NCBI, a gene ontology database (GO) [60], the CDD of the NCBI [61] and a custom-prepared subset of the NCBI nucleotide database containing either mitochondrial or rRNA sequences.

As indicated in our previous work [14,15], "because the libraries used are unidirectional, three-frame translations of the dataset were also derived, and open reading frames starting with a methionine and longer than 40-AA residues were submitted to the SignalP server [62] to help identify putatively secreted (S) proteins. The EST assembly, BLAST, and signal peptide results were loaded into an Excel spreadsheet for manual annotation and are provided in additional File 1."

**Figure 1:**
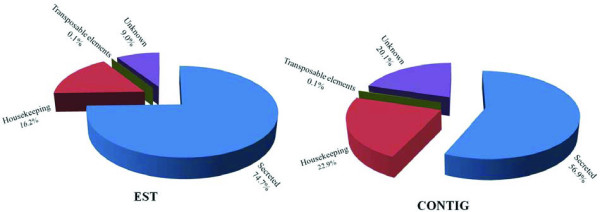
**Functional class distribution of expressed sequence tags (EST) or assembled contigs (Contigs) deriving from a salivary gland cDNA library from female adult *Simulium guianense *black flies**.

Four categories of expressed genes derived from the manual annotation of the contigs were created (Table 1 and Figure 1). The S category contained 56.9% of the clusters and 74.7% of the sequences, with an average of 3.1 sequences per cluster. This value is 46% larger (∑χ^2 ^= 44.2; *p *= 2.9E^-11^) than that seen in *S. vittatum*, where only 51% of ESTs encode S proteins, and 21.4% larger than in *S. nigrimanum *(∑χ^2 ^= 14.4; *p *= 0.00014).

**Table 1:** Functional classification of salivary transcripts originating from the salivary glands of *Simulium guianense*

Class	Number of ESTs	Number of Contigs	ESTs/Contig
Secreted products	1324	428	3.1
Housekeeping	288	172	1.7
Transposable element	1	1	1.0
Unknown products	159	151	1.1

Total	1772	752	

The housekeeping (H) category had 22.9% and 16.2% of the clusters and sequences, respectively, and an average of 1.7 sequences per cluster. One singleton was classified as a transposable element (TE), constituting less than 0.1% of the ESTs or contigs. TEs are a common finding in hematophagous sialotranscriptomes and most probably reflect regulatory transcripts repressing transposition rather than active transposition [63]. Transcripts with matches to TE were also found in *S. nigrimanum *sialotranscriptome [15]. Finally, 20.1% of the clusters, containing 9.0% of all sequences, were classified as unknown (U), because no functional assignment could be made. This category had an average of 1.1 sequences per cluster, and most of these consisted of singletons. A good proportion of these transcripts could derive from 3'or 5' untranslated regions of genes of the previous two categories, as was indicated for a sialotranscriptome of *Anopheles gambiae *[64].

### S Class of Expressed Genes

Inspection of Additional File 1 indicates 1,324 ESTs associated to secreted products that were characterized to several families, including ubiquitous proteins families such as Antigen-5, Kunitz domain-containing polypeptides, trypsin, amylase/maltase, apyrase, hyaluronidase, and lysozyme. Insect-specific families, such as Aegyptin and D7/OBP superfamily, were also found. About 63.6% of all secreted products revealed exclusively *Simulium*-specific families, where the SVEP family contained 22.5% of the sequences. Others *Simulium*-specific families were found such as collagen-like peptide, poly-Q mucin, and glycine histidine-rich. Some of these families (Table 2) were found in previous transcriptomes from black flies [14,15]. Novel putative families were deorphanized in *S. nigrimanum*.

**Table 2:** Functional classification of putative secreted transcripts originating from the salivary glands of *Simulium guianense*

Family	Number of ESTs	Contig	ESTs/Cluster
Ubiquitous protein families			
Antigen-5 family	5	2	2.5
Yellow family	1	1	1.0
ML domain family	1	1	1.0
Lipocalins	2	2	1.0
Immunity related products	36	12	3.0
Protease inhibitor domains			
Serpin	1	1	1.0
Kunitz-domain protease inhibitor	19	9	2.1
Enzymes			
Trypsin	69	28	2.5
Hyaluronidase	5	1	5.0
Apyrase	14	4	3.5
Amylase	61	14	4.4
Adenosine deaminase	1	1	1.0
Destabilase	1	1	1.0
Insect-specific families			
Aegyptin family	23	11	2.1
D7 family (OBP superfamily)	242	72	3.4
*Simulium*-specific families			
SVEP vasodilator family	190	56	3.4
Other *Simulium*-specific families	427	146	2.9
PolyQ mucin family	51	16	3.2
GH repeat family	127	33	3.8
Collagen-like family	48	17	2.8

Total	1324	428	

### H Genes

The 172 clusters (comprising 288 ESTs) attributed to H genes expressed in the salivary glands of *S. guianense *were further divided into 15 subgroups according to function (Table 3). Not surprisingly for an organ specializing in the secretion of polypeptides, the two larger sets within the H category were associated with protein synthesis machinery (71 clusters containing 113 ESTs; 39.2%) and energy metabolism (28 clusters containing 33 ESTs; 11.4%). This pattern was also observed in other sialotranscriptomes of hematophagous insects [22,65].

**Table 3:** Functional classification of housekeeping transcripts originating from the salivary glands of *Simulium guianense*

Function	Number of ESTs	Contig	ESTs/Cluster
Protein synthesis	113	71	1.6
Energy metabolism	33	28	1.2
Signal transduction	18	14	1.3
Transporters and storage	5	5	1.0
Proteasome machinery	1	1	1.0
Protein modification machinery	6	6	1.0
Protein export machinery	4	4	1.0
Nuclear regulation	2	2	1.0
Nucleotide metabolism	2	2	1.0
Transcription machinery	2	2	1.0
Carbohydrate metabolism	3	3	1.0
Amino acid metabolism	1	1	1.0
Detoxification metabolism	3	3	1.0
Cytoskeletal	2	2	1.0
Unknown conserved	93	28	3.3

Total	288	172	

Exceptionally, the protein synthesis class revealed a significant increase of 38% more ESTs in *S. guianense *compared with *S. vittatum *(∑χ^2 ^= 18.9; *p *= 1.35E^-05^). This increase was also observed in relation to *S. nigrimanum *(15.3%), but it was not significant statistically (∑χ^2 ^= 0.76; *p *= 0.38).

We have arbitrarily included a group of 93 ESTs (32.3%) with 28 clusters in the H category that represent highly conserved proteins of unknown function, presumably associated with cellular function. Previously, sialomes of *S. vittatum *and *S. nigrimanum *described 24% and 27% of ESTs to this category, respectively. Here, *S. guianense *also revealed an increase of 34.5% and 19.6% more ESTs than the species described above, respectively, which was statistically significant only in reference to *S. nigrimanum *(∑χ^2 ^= 18.4; *p *= 1.77E^-05^). They are named "unknown conserved" in Additional File 1. These sets may help functional identification of the "conserved hypothetical" proteins as previously reviewed by Galperin and Koonin [66]. The complete list of all 288 gene clusters, along with further information about each, is given in Table 3 and Additional File 1.

### Analysis of the *S. guianense *Sialome

**Figure 2:**
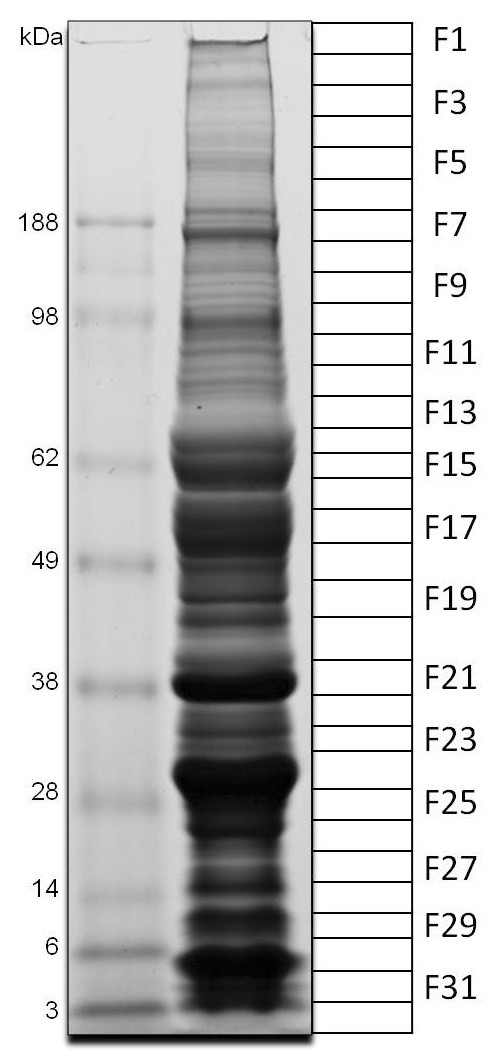
**1-D gel electrophoresis of *Simulium guianense *salivary gland homogenates**. The numbers at the left indicate the mol wt of the protein standards (kDa), shown in the left lane. The right gel lane shows the separation of the salivary gland proteins. The grid at the right (F1-32) represents the gel slices submitted for tryptic digest and tandem mass spectrometry identification.

Several clusters of sequences coding for H and S polypeptides indicated in Additional File 1 are abundant and complete enough to extract novel consensus sequences. A total of 174 novel sequences--164 of which code for S proteins-- are grouped together in Additional File 2. With this database, we characterized the proteome via analysis of SDS-PAGE separated proteins that were tryptic digested and submitted to MS/MS analysis (Figure 2). The results of this experiment are integrated within the description of the deduced proteins from the transcriptome analysis. Here, we used proteome analysis to confirm 28 of the 32 protein families found in this sialotranscriptome (Additional File 2), which are described in more detail below. The reader is here informed that the introduction of the diverse protein families may contain text previously used in our publications on Simulium sialomes [14,15] and such text will appear in "quotes".

### Functional Classification of S Families from *S. guianense*

#### Ubiquitous families or domains

#### Enzymes

Several transcripts found in the sialotranscriptome *S. guianense *encode proteins with sequence similarity to several secreted enzymes such as glycosidase, serine proteases, hyaluronidases, apyrase, adenosine deaminase (ADA), and destabilase. They can be associated with blood feeding, sugar feeding, or both, as follows:

##### Glycosidases

**Figure 3:**
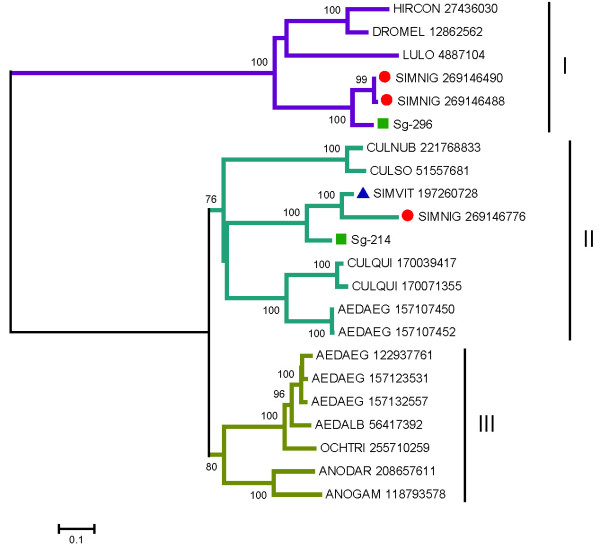
**The amylase/maltase family in Diptera**. Phylogram derived from the alignment of two *Simulium guianense *proteins (indicated by a square and starting with "Sg-"), with their best matches in the non-redundant protein database of the National Center for Biotechnology Information identified by the three first letters of the genus name, followed by the three first letters of their species name, followed by their NCBI accession numbers. Glycosidases from *Simulium vittatum *and *Simulium nigrimanum *are indicated by a triangle and a circle, respectively. The numbers on the tree bifurcations indicate the percentage bootstrap support above 75%. The bar at the bottom represents 10% amino acid substitution. Protein sequences were aligned by the Clustal program, and the dendogram was made with the Mega package after 10 000 bootstraps with the neighbor-joining algorithm.

"Amylases and maltases are ubiquitous enzymes that help digestion of carbohydrates and are commonly found in sialotrancriptomes from Nematocera including mosquitoes, biting midges, sand flies, and black flies [67]. These enzymes can be recognized by the KOG motif 0471, named Alpha-amylase." The proteome of the mosquito *An. gambiae *has 17 members of this family, one of which (AGAP002102) is expressed in the SGs [64]. The proteome of *Ae. aegypti *contains 24 such enzymes, at least two of which are expressed in their SGs [25] while *Culex quinquefasciatus *has 35 such enzymes, with two also expressed in their SGs [68]. Additional File 2 presents two truncated gene products coding for glycosidases (Sg-214 and Sg-296). Glycosidases of *S. guianense *have 79% sequence identity to other described black fly enzymes (blastp comparisons can be seen in Additional File 2). Phylogenetic analysis of the *S. guianense *protein sequences together with their closest BLAST matches against the NR database indicates that the two *S. guianense *proteins group into different clades with strong bootstrap support (Figure 3). Sg-296 groups to other *Simulium *enzymes, to a salivary sand fly enzyme, and to drosophilids, as indicated by clade I (Figure 3). Sg-214, on the other hand, groups with a second set of *Simulium *enzymes and, with 76% bootstrap support, to Culicine mosquitoes and salivary biting midge enzymes,[34,69] as shown by clade II (Figure 3). Notice that the mosquitoes, black flies, and *Culicoides *sequences each group within subclades having strong bootstrap support, as expected. A third clade of mosquito-only enzymes (including anophelines and culicines) is also obtained, which merges without strong bootstrap support to Clade II. Interestingly, the mosquito enzymes in both clades II and III have all been previously described in salivary transcriptomes, suggesting a common origin of these sugar-hydrolyzing enzymes in the ancestral fly originating mosquitoes, black flies, and biting midges. These results indicate that the two *S. guianense *sequences appear to be a product of ancient gene duplication, Sg-296 from Clade I being the most ancient, as it groups with enzymes of Brachycera, while the two salivary gene products from Culicine mosquitoes appear to derive from a gene duplication after the split of the Culicidae. The sequences of the glycosidases Sg-214 and Sg-296 found in the sialotranscriptome of *S. guianense *were confirmed by proteome analysis within the fractions 16 and 17, respectively, just above the 49-kDa standard (Figure 2 and Table 4).

**Table 4:** Putative secreted proteins deduced from the sialotranscriptome analysis and indication of expression by proteomic analysis

Description	Protein name | Fraction → number of peptides
Laminin-like	Sg-431|F14→ 2
Amylase/maltase	Sg-214|F16→116, Sg-296|F17→32
Apyrase	Sg-354|F16→58
	Sg-126|F16→12, Sg-129|F16→12, Sg-121|F16→12,
*Simulium *mucin	Sg-120|F16→11, Sg-127|F16→11, Sg-117|F16→10,
	Sg-128|F16→10, Sg-119|F16→9, Sg-125|F9→7
Hyaluronidase	Sg-414|F20→14
Diptera secreted protein from conserved insect	Sg-215|F21→134, Sg-216|F21→133, Sg-292|F17→49,
	Sg-347|F14→36, Sg-256|F13→28
*Simulium *collagen-like	Sg-152|F23→87, Sg-149|F23→84
Acid 28-kDa	Sg-320|F23→45, Sg-321|F23→41, Sg-319|F23→23
Long D7	Sg-261|F26→42, Sg-220|F23→24, Sg-218|F23→14
Serine proteases	Sg-244|F24→48, Sg-138|F25→28, Sg-416| F24→6
Antigen-5	Sg-457|F24→30
Aegyptin	Sg-276|F24→37
*Simulium *basic 28-kDa	Sg-136|F24→34
Deorphanized 19-kDa	Sg-303|F27→17, Sg-309|F27→9
Sv 7.8-kDa	Sg-356|F27→11, Sg-372|F30→9, Sg-227|F32→4, Sg-
	205|F31→3
D7 16-kDa	Sg-331|F29→14, Sg-350|F30→9
*Simulium *basic 13 kDa	Sg-446|F29→2
Deorphanized Sn 8-10 Cys W	Sg-340|F29→22, Sg-324|F29→12
Lysozyme	Sg-263|F30→7
Kunitz domain	Sg-395|F30→2
	Sg-1|F30→164, Sg-8|F30→149, Sg-92|F30→53, Sg-
	102|F30→43, Sg-94|F30→34, Sg-93|F30→33, Sg-
SVEP	100|F30→32, Sg-95|F30→26, Sg-90|F30→25, Sg-
	99|F30→25, Sg-103|F30→22, Sg-101|F30→20, Sg-
	344|F30→14
5-Cys *Simulium*	Sg-282|F30→3
Basic 7-13 *Simulium*	Sg-403|F30→8, Sg-420|F31→3
Cecropin	Sg-368|F31→2, Sg-369|F31→3
Ultra-short D7	Sg-383|F31→3
*Simulium *basic 7.4 kDa	Sg-422|F31→4
Deorphanized 8 kDa basic	Sg-258|F31→5
Similar to Kunitoxin	Sg-375|F31→4

##### Serine proteases

**Figure 4:**
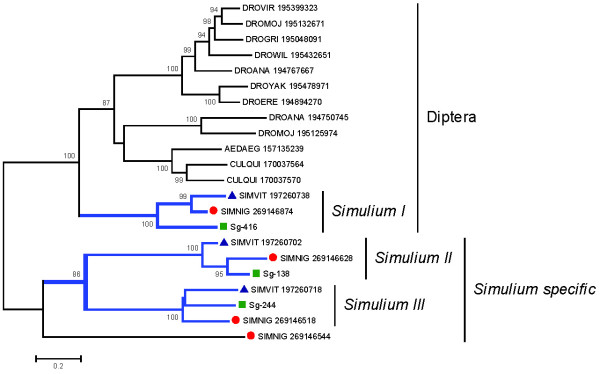
**Phylogeny of the salivary serine protease family of *S. Guianense***. Phylogram derived from the alignment of three *Simulium guianense *proteins and their best matches from the non-redundant protein database from the National Center for Biotechnology Information. *S. guianense *proteins are indicated by a square and start with Sg-. Serine proteases from *Simulium nigrimanum *(SIMNIG) are indicated by a circle and in *Simulium vittatum *(SIMVIT) by a triangle, with their respective accession numbers. The numbers on the tree bifurcations indicate the percentage bootstrap support above 75%. The bar at the bottom represents 20% amino acid substitution. Protein sequences were aligned by the Clustal program, and the dendogram was made with the Mega package after 10 000 bootstraps with the neighbor-joining algorithm. For other details, see Figure 3.

"Serine proteases are commonly found within hematophagous insect sialomes [70] except in sand flies, where it was only found in the *Phlebotomus ariasi *sialome [30]. This family has an important role in the immune system, acting as prophenoloxidase activators or in digesting skin matrix components such as in an elastase function, or hydrolyzing host blood-clotting components such as fibrinogen/fibrin, or activating plasminogen [71,72]. In *Ae. aegypti*, transcripts coding for serine protease with the CUB domain were reported, indicating specialized substrate recognition [73]." The sialotrancriptome of *S. guianense *allowed the identification of transcripts coding for three secreted serine proteases varying with predicted mol wt between 25.4 and 27 kDa, which may derive from three polymorphic genes. Alignment of representative members of these three gene products from *S. guianense *(Sg-416, Sg-138, and Sg-244) with their best BLAST matches (only Diptera sequences were included) produces a phylogram indicative of one clade that is related to mosquito and fruit fly sequences with strong bootstrap support (Figure 4) and one additional *Simulium*-specific clade, the latter containing two sub clades. This *Simulium*-specific clade is quite divergent, having only 27% or less identity to their best Diptera match, indicating fast evolution of this clade. The sub-clades (marked as *Simulium *I, II, and III in Figure 4) each contain one enzyme from each of the Simulidae thus far analyzed for their sialotranscriptome, indicating conservation of these threee salivary expressed genes in black flies. An additional *S. nigrimanum *sequence is also found in this *Simulium*-specific clade, indicating that a fourth gene may be expressed in this fly. The serine protease proteins found in the sialotranscriptome of *S. guianense *were confirmed by proteome analysis within the fractions F24 and F25, located near the 28-kDa marker, consistent with its predicted (25 to 27 kDa) mature weight of these proteins (Figure 2 and Table 4).

##### Hyaluronidases

Hyaluronidases are enzymes that cleave hyaluronic acid, which is a main component of the extracellular matrix in vertebrates. This enzyme was first described in saliva of New World *Lutzomyia longipalpis *[74] and thereafter in the SGs of several other Old and New sand fly species [75,76]. It was also reported in *S. vittatum *[77]. Hyaluronidases also have been described in the sialotranscriptome of *C. quinquesfaciatus *[68] and *Glossina morsitans morsitans *[51]. Interestingly, although *Phlebotomus papatasi *and *Phlebotomus dubosqui *SGHs displayed hyaluronidase activity, no such transcripts were found in their cDNA libraries [75]. Hyaluronidase transcripts were also absent from *S. vittatum *and *S. nigrimanum *sialotranscripomes [14,15]. Here, we found one full-length sequence (with five ESTs) coding for a protein with 37.8 mol wt and pI 9.2 matching the pfam01630 domain named "Glyco_hydro_56, Hyaluronidase" with an e value of 1e^-61^. The NR database of the NCBI revealed identities above 43% to hyaluronidases from *Lu. longipalpis *and *Phlebotomus arabicus *in addition to matching other insect enzymes from *Pediculus humanus *and some vespids; however, these non-dipteran sequences were only 34% identical at the AA sequence level. Fourteen tryptic peptides obtained by MS/MS had matches to hyaluronidase protein within fraction 20, just above the 38-kDa standard and consistent with the predicted 37-kDa mature mol wt of this protein (Figure 2 and Table 4).

##### Apyrase

**Figure 5:**
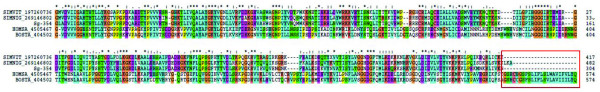
**Clustal alignment of the carboxytermini of black fly salivary 5' nucleotidases/apyrase compared with mammalian enzymes**. The *Simulium guianense *protein (Sg-354) and black fly homologs are compared with the human and bovine sequences. The box shows the deletion of the membrane anchor region in the salivary apyrases of black flies. The symbols above the alignment indicate (*) identical sites; (:) conserved sites, and (.) less-conserved sites. For other details, see Figure 3.

"This enzyme hydrolyzes ATP and ADP to AMP and orthophosphates and has been commonly found in blood feeding arthropods, where it has been suggested as a typical case of convergent evolution [67]. Because ADP and ATP are important activators of platelet and neutrophils, apyrase activity removes these agonists of hemostasis and inflammation [78]. Different genes have been described for this activity such as members of the 5'-nucleotidase family in mosquitoes and triatomines [79-82], the *Cimex*-type apyrase family in bed bugs and sand flies [83,84] and the type CD-39 protein family in fleas [52]. Expression of this enzyme in mosquitoes has helped to understand the feeding preference in *Anopheles, Aedes*, and *Culex *genus [85]. As *Culex *has birds as the primary source of blood and does not face the platelet barrier, members of this genus reveal little or absent expression of this enzyme [72]." In black flies, this enzyme activity was previously described in SGHs from several species with different degrees of anthropophy or zoophilic, gonotrophic cycle and vector or non-vector status, revealing dependence on Ca^+2 ^or Mg^+2 ^ions for activation and with positive association to species with confirmed vector status for *O. volvulus *[8,86]. While we do not know the origin of black fly salivary apyrases, transcripts coding for members of the 5'-nucleotidase family have been previously described in *S. vitattum *and *S. nigrimanum *[14,15]. Studies in black fly sialotrancriptomes also revealed an increase in the expression of putative apyrase transcripts in *S. nigrimanum *when compared with *S. vittatum*, with statistically significant difference (*p *= 0.00337) [15]. The 5'-nucleotidases are ubiquitous enzymes usually found bound to the extracellular face of biologic membranes through a glycophosphatidyl-inositol phosphate anchor [87,88]. However, salivary secreted enzymes of mosquitoes [80] and triatomine bugs [81] lack the carboxyterminal domain where the glycolypid is anchored, allowing their secretion. Here, we found 14 transcripts coding for the putative salivary apyrase of *S. guianense *(Additional File 1). Alignment of the putative apyrase of *S. guianense *with their simulid homologs plus vertebrate sequences known to be membrane anchored reveals the lack of the carboxyterminal site for the glycolipid anchor in *S. guianense *(Figure 5), as was also found for other *Simulium *putative apyrases [14,15], indicating the *S. guianense *enzyme to be secreted. Fifty-eight tryptic peptides were deducted by MS/MS with matches to apyrase protein (Sg-354) originated from fraction 16, located just below the 62-kDa standard (Figure 2 and Table 4).

##### Adenosine deaminase (ADA)

ADA transcripts, although previously found in sialotranscriptome of mosquitoes and sand flies,[16,26,29,73,74,89,90] here appear for the first time in black fly sialotranscriptomes. *Ae. aegypti *salivary homogenates hydrolyze adenosine (Ado) to inosine, and then to hypoxantine plus ribose, with enzymatic activities in saliva and SGHs [91,92]. Recombinant ADA from *P. dubosqi *was shown to have potent activity [93]. Here, we found a singleton EST producing one truncated sequence with 68% identity with *Ae. aegypti *ADA. Puzzlingly, Ado is a powerful antiplatelet and vasodilator, and the presence of a salivary ADA should be considered non-adaptative; however, Ado is also a potent inducer of mast cell degranulation, and for this reason it may be in the insect's interest to remove this product. Interestingly, *P. papatasi *does not codify transcripts to ADA but contains Ado and AMP in its saliva, which acts as the main salivary vasodilator [94].

##### Destabilase

"This enzyme is an endo-ε-(γ-Glu)-Lys isopeptidase, which cleaves isopeptide bonds formed by transglutaminase (Factor XIIIa) between Gln glutamine γ-carboxamide and ε-amino groups of lysine and was first described in the saliva of leeches. Its activity leads to dissolution of stabilized fibrin [67]. Destabilases are members of the lysozyme superfamily of proteins [95,96]." A 3' truncated singleton EST is 86% identical to *S. nigrimanum *destabilase (Additional File 1). It is still unknown whether salivary homogenates of black flies have destabilase activity.

#### Protease inhibitor domains

##### Serine protease inhibitors (serpins)

Serpins were previously reported in sialomes of *Ae. aegypti, Ae. albopictus, Ochlerotatus triseriatus *and *Lu. longipalpis*. The gene coding for the protein gi|3411116 in *Ae. aegypti *represents the main salivary anticlotting protein in this mosquito with specificity to fXa [97,98], having as homologue the protein gi|56417456 in *Ae. albopictus*. Targets of others serpins found in mosquito sialotranscriptomes are unknown [16]. Here, we found one singleton EST (Sg-500) coding for a 3' truncated serpin with 60% identity to a homologous serine protease inhibitor from *Ae. aegypti *and *An. gambiae*. Serpins have not been found in black fly sialotranscriptomes, possibly because these flies use Kunitz-domain proteins as anticlotting agents.

##### Kunitz-domain protease inhibitors

"Kunitz domain-containing proteins are associated with protease inhibitors and so far have been found in sialotranscriptomes of Nematocera black flies and biting midges but not in mosquitoes, sand flies, or bloodsucking Hemiptera. Kunitz domain-containing proteins, however, are abundant in tick sialotranscriptomes." Hematophagous arthropods secrete protease inhibitors that can act in specific points of the coagulation cascade, mainly against thrombin or factor Xa (or both). This activity has been previously described in SGHs of several black fly species such as *S. vittatum, S. ochraceum, S. argus*, and *S. metallicum *[5-7,99] and *Culicoides *midges [100]. The sialotranscriptome of *S. guianense *contains a typical single Kunitz protein deducted from three ESTs (Sg-395). This protein has its best blastp match to its homologous *S. nigrimanum *(72% of identity) and *S. vittatum *(58%) proteins. Previously, *S. vittatum *salivary homogenates were shown to have potent fXa inhibitory activity, but its molecular nature remains unknown [5]. Salivary homogenates of *S. guianense *also inhibited the same target of the coagulation cascade (data not published). It is possible that the fXa inhibitor of *Simulium *resides in a Kunitz domain-containing protein. Two tryptic peptides obtained by MS/MS matching Kunitz-domain protein (Sg-395) were found within fraction 30, located just below the 6-kDa standard (Figure 2 and Table 4).

#### Ubiquitous protein families

##### Immunity-related products

In this group, we found full coding sequences to two ubiquitous antimicrobial peptides: lysozyme and cepropin. These families are commonly found in hematophagous arthropods, and their presence was previously reported in black fly sialotrancriptomes [14,15].

The *S. guianense *sialotranscriptome revealed 25 ESTs coding for members of the lysozyme family (Additional File 1), where several possible alleles of the same gene were identified. *S. guianense *salivary lysozyme is 79% identical to *S. nigrimanum *salivary lysozyme and 52% identical to its closest mosquito relative (Additional File 2). Lysozyme activity in *S. guianense *was confirmed to exist in SGHs (unpublished, Chagas). This activity was described in both male and female mosquitoes [101,102]. Seven tryptic peptides deducted by MS/MS had matches to lysozyme protein (Sg-263) within fraction 30, located just below the 6-kDa standard (Figure 2 and Table 4).

Cecropins are small secreted basic proteins of 3 kDa mol wt, rich in aliphatic AAs, mainly Val, with highest conservation in its carboxy terminal region. Ten ESTs from the *S. guianense *sialotranscriptome code for two closely related, possibly allelic, cecropins. These cecropins, as expected, have their best matches to other *Simulium *and mosquito cecropins. The cecropin peptides (Sg-368 and Sg-369) found in the sialotranscriptome of *S. guianense *were confirmed by proteome analysis within the fraction 31, located just above the 3-kDa marker, consistent with the predicted 3-kDa mature mol wt of this protein (Figure 2 and Table 4).

##### Antigen-5 family

**Figure 6:**
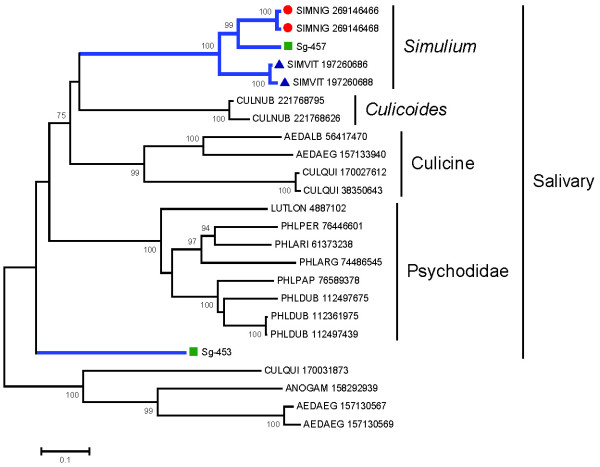
**Phylogram of two Antigen-5 proteins derived from *Simulium guianense *sialotranscriptome with their best matches from the non-redundant protein database from the National Center for Biotechnology Information**. The *S. guianense *proteins (Sg-457 and Sg-453) are indicates by a square, *Simulium vittatum *proteins by a triangle, and *S. nigrimanum *proteins by a circle. The numbers on the tree bifurcations indicate the percentage bootstrap support above 75%. The bar at the bottom represents 10% amino acid substitution. Protein sequences were aligned by the Clustal program, and the dendogram was made with the Mega package after 10 000 bootstraps with the neighbor-joining algorithm. For other details, see Figure 3.

**"**This ubiquitous family belongs to the wider CAP superfamily [103]. Most members have no known function, but a few have been related to pathogen defenses in plants, as toxins in snake and lizard venoms [104,105], as a platelet aggregation inhibitor in a tabanid fly [106], and as a possible inhibitor of the classical pathway of complement activation in the stable fly [107,108]." Members of this family are found in all nematoceram sialotranscriptomes [67]. The annotated *An. gambiae *proteome reveals 21 proteins for this family, 2 of which are expressed in the SGs [64]. Similarly, the *Ae. aegypti *and *C. quinquefasciatus *proteome have over 30 members of the family, of which at least 2 are expressed in their SGs [25,68]. The *S. guianense *sialotranscriptome reveals two clusters coding for CAP family members. The phylogram resulting of the alignment of the two *S. guianense *proteins with their 25 best blastp matches from the NR protein database (excluding drosophilid proteins) reveals one clade of bloodsucking Nematocera with relatively strong bootstrap support (70%), and strong bootstrap support for the sub-clades containing each of the four families. Interestingly, all members of this clade (marked as "Salivary" in Figure 6) were found in sialotrancriptomes, suggesting a common salivary ancestor for this particular CAP-coding gene within Nematocera. The *S. guianense *protein Sg-453 is outside this clade and may represent an additional *Simulium *gene member of the CAP family that has been recruited for a salivary function. Thirty tryptic peptides deducted by MS/MS had matches for an Antigen-5 protein (Sg-457) within fraction 24, located just above the 28-kDa marked, near the predicted 30-kDa mature mol wt of this protein (Figure 2 and Table 4).

##### Yellow family

This family is insect-specific in eukaryotes and received this name due to mutation of a gene that induces a yellow phenotype in Drosophila, resulting from the disruption of melanin formation. In Nematocera, this family is abundantly expressed in sand flies and has been suggested as important markers of vector exposure [109]. *S. guianense *reveals only one EST to Yellow protein with a match to Yellow of *C. quinquesfaciatus *(64% identity). Interestly, *S. vittatum *also reveals one transcript to the Yellow family [14]. The function of this protein in *Simulium *remains unknown.

##### ML domain family

*S. guianense *contains one transcript coding for a protein containing the ML domain, which was not previously reported in black fly sialomes. This domain is implicated in lipid recognition, particularly in the recognition of pathogen-related products, but could also have a lysosomal function [110] and thus have a housekeeping function. It has an immunoglobulin-like β-sandwich fold similar to that of E-set. The blast to NR database suggests similarity to Niemann-Pick Type C-2 putative from *An. aegypti *(also known as Epipidymal secretory protein E1) and similarities to MPA2 allergen from *Nasonia vitripennis *(Hymenoptera). The function of this protein in *S. guianense *remains unknown.

##### Lipocalins

Lipocalins are proteins widely distributed in animals and plants. This protein family is highly expressed in triatomines, such as *Rhodnius prolixus, Triatoma infestans *[47], *Triatoma brasiliensis *[111] and *Dipetalogaster maxima *[112]. In triatomines, lipocalins are reported as carriers of nitric oxide, kratagonists (binders of agonists) of biogenic amines, Ado nucleotides, and thromboxane A_2_, as well as inhibitors of collagen-induced platelet aggregation and thrombin and as allergens [70,113-116]. We found two ESTs coding for different lipocalins (Sg-671 and Sg-568). The deducted AA sequence of both transcripts matches human apolipoprotein in the Swissprot database. If secreted in saliva, these proteins are candidates for agonist lipid binding.

#### Insect-specific families

##### Aegyptin family

This protein family, commonly found in sialotranscriptomes of mosquitoes, was first named as 30-kDa *Aedes *allergen [117] and as GE-rich protein [89]. It has revealed high levels of expression in the sialotranscriptome of *Anopheles funestus *[18]. Functional analyses of an *Ae. aegypti *family member, named Aegyptin, as well as a member from *An. stephensi*, have demonstrated its function as an antagonist of collagen-induced platelet aggregation and as a useful tool for inhibiting platelet-collagen interaction *in vitro *and *in vivo *[18,72,118]. Previous black fly sialotranscriptomes have revealed proteins similar to Aegyptin, supporting the common origin of hematophagy in mosquitoes and black flies as proposed by Grimaldi and Engel [53]. Here, we found 23 ESTs of the S class (Additional File 1) coding for what appear to be alleles of a single gene similar to Aegyptin-like proteins, showing 60% identity to Aegyptins from sialotranscriptomes of black flies and 35% to mosquito homologs. The alignment revealed GE-rich regions mainly in the middle of the sequences (not shown). Thirty-seven tryptic peptides obtained by MS/MS had matches to Aegyptin protein (Sg-276) within fraction 24, located just above the 28-kDa marker, consistent with predicted (28 kDa) mature weight of this protein (Figure 2 and Table 4).

##### Diptera Secreted Protein from Conserved Insect Family

Five proteins found in sialotranscriptome of *S. guianense *were similar to secreted protein from insects. This family was previously described in *S. nigrimanum *sialotranscriptome. They vary between 37 and 57 kDa mol wt with pI 6.1 to 9.6. The best matches to the NR database showed similarities to several families of Diptera (Culicidae, Ceratopogonidae, Drosophilidae) and Hymenoptera (Pteromalidae and Formicidae) maintaining a low degree of conserved AA. The phylogram suggests at least four different genes to exist in *S. guianense*. Pattern-initiated PSI-BLAST (PHI-BLAST) using the initiation pattern G-x-[MLI]-x(6)-[WF]-x(7,12)-[KNE]-x-[IMFL]-x(37,40)-[VIL]-x-[YF]-x(3)-[QKR]-x(14)-[IL]-x(5,6)-[NDE]-x(5)-[ILV]-[AS] shows the diversity of this protein family within insects (Additional File 3).

##### Laminin-like Secreted Salivary Protein Found in *Simulium *and *Culicoides*

Laminin-like proteins have been suggested to be extracellular matrix proteins [34]. The *S. guianense *sialotranscriptome revealed one truncated protein (Sg-431) with four ESTs coding to a *S. vittatum *homolog (76% identity) and to a *Culicoides nubeculosus *protein with 32% identity. Two tryptic peptides obtained by MS/MS had matches for laminin-like protein within fraction 14, just above the 62-kDa standard (Figure 2 and Table 4).

##### D7/OBP superfamily

"The odorant-binding protein family is ubiquitous in insects. The D7 protein family, specific to bloodsucking Nematocera, is recognized as a member of the OBP superfamily [119,120] but it contains two additional α-helices [121,122]. Short and long forms of the D7 family exist in which one or two D7 domains exist in the same protein, producing proteins with mature mol wt of ~18 or ~28 kDa. In *Simulium*, an extra-short family with ~12 kDa is also found, reminiscent of sand fly salivary proteins, which also have an ultra-short form but bear no similarities to the black fly proteins at the AA level [67]. "The *S. guianense *OBP/D7 sequences were grouped in the three subfamilies described below.

Long D7 family: Two proteins with two OBP domains are recognized in the *S. guianense *sialotranscriptome. When searched against the NR database using blastp, these proteins only produce significant matches to other *Simulium *proteins, but all three produce two matches each to the PFAM PBP_GOBP domain when using the tool rpsblast, one in the first half and the other in the second half of the protein. A third truncated protein has only one OBP domain but matches long D7 proteins of *S. nigrimanum*. Long D7 proteins had tryptic peptides deducted by MS/MS within fractions F23 and F26 near the 28-kDa standard, consistent with the predicted (28 kDa and 24 kDa) mature weight of these proteins (Figure 2 and Table 4).

D7 16-kDa family: Two *S. guianense *proteins containing one OBP domain were found. Sg-331 produces significant similarities only to other *Simulium *proteins, but Sg-350 additionally retrieves OBP from *C. quinquefasciatus*. Tryptic peptides were deducted by MS/MS matches to D7 16-kDa proteins within the fractions 29 and 30, just above the 6-kDa marker (Figure 2 and Table 4).

Ultra-short D7 proteins (10-12 kDa mature weight): This was the most expressed family within the D7/OBP superfamily, encompassing 80 ESTs (Additional File 1). All clusters contain signal peptide, suggesting secreted proteins. These data suggest the existence of at least four genes coding for ultra-short D7 proteins (Sg-75, Sg-190, Sg-363, and Sg-383) and several possible alleles. Only Sg-383 was deducted by MS/MS within fraction 31, just below the 6-kDa marker (Figure 2 and Table 4).

#### *Simulium*-specific families

Of the 1,324 ESTs of the S class, 843 ESTs are specific to *Simulium*, encompassing 18 specific families. Some families from *S. nigrimanum *were deorphanized, and a new family coding to Kunitoxin-like proteins was first found in insect sialotranscriptomes. More details of these families are described below.

##### SVEP vasodilator family

**Figure 7:**
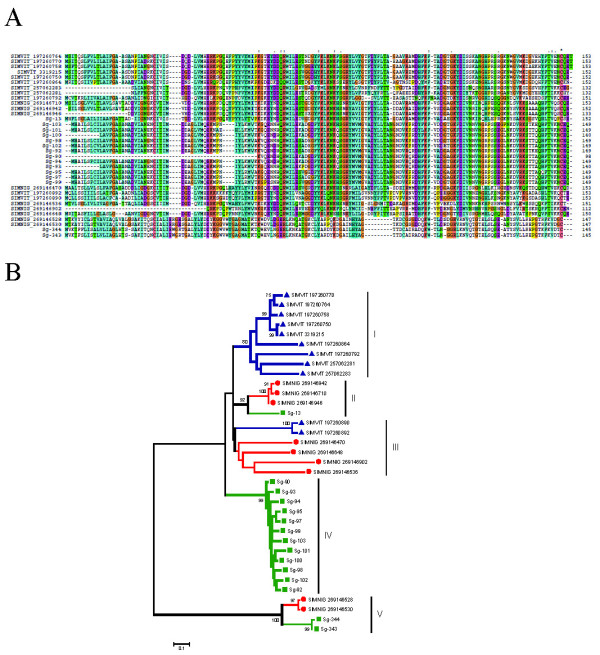
**The *Simulium vittatum *erythema protein (SVEP) superfamily of *Simulium***. (A) Clustal alignment of all SVEP proteins derived from black fly sialotranscriptomes. The symbols above the alignment indicate (*) identical sites, (:) conserved sites, and (.) less-conserved sites. (B) Bootstrapped phylogram derived from the alignment in (A). *Simulium guianense *proteins are indicated by a square, *S. vittatum *by a triangle, and *Simulium nigrimanum *by a circle. The numbers on the tree bifurcations indicate the percentage bootstrap support above 75%. The bar at the bottom represents 10% amino acid substitution. Protein sequences were aligned by the Clustal program, and the dendogram was made with the Mega package after 10 000 bootstraps with the neighbor-joining algorithm. For other details, see Figure 3.

**"**This family is specific to black flies and was originally described in SGHs of *S. vittatum*, when it was named *Simulium *vasodilator erythema protein (SVEP) because it produced a prolonged vasodilation when tested in rabbit skin [10]. A recombinant protein (rSVEP) was expressed and functionally characterized as a potent vasodilator, possibly activating ATP-dependent K+ channels [123]. This property has an important role during blood feeding and was suggested as one key compound of the competence vector of these flies in the transmission of *Onchocerca *parasites [124]." Sialotranscriptomes of two black fly species identified SVEP to belong to a diverse multigene family with at least five genes for each species [14,15]. The sialotranscriptome of *S. guianense *also revealed proteins homologous to SVEP, totaling 190 ESTs with identities to other *Simulium *SVEPs varying from 50 to 70%. Alignment of members of this family showed sequences with similar sizes but with few conserved AAs. Comparative phylogenetic analysis of all SVEP proteins, after 10 000 bootstraps grouped the majority of the members of *S. vittatum *in a specific clade (I) with 80% bootstrap support (Figure 7). The phylogram indicates at least three genes and several either recent gene duplications and/or alleles coding to members of this family (clade IV on Figure 7). The protein Sg-13 shares clade II, with 92% of bootstrap support, with its homologous *S. nigrimanum *proteins. ESTs coding for Sg-13 or very closely related proteins represent more than 50% of the sequences coding for SVEP members in this sialotranscriptome. Clade III groups only SVEPs from *S. vittatum *and *S. nigrimanum *without bootstrap support. Clade IV reveals a possible case of gene duplication or expression (or both) of a very polymorphic gene from *S. guianense*, and the last clade (V) groups two clusters (Sg-344 and Sg-343) of *S. guianense *with its homologous *S. nigrimanum *(with 100% bootstrap support), which appear completely distinct from other SVEP proteins. This scenario indicates that at least two genes (Sg-13 and Sg-344) have common ancestors with *S. nigrimanum *and a third gene could have given rise to the increased of expression of this protein family, shown in clade IV, possibly with many recent gene duplications. Interestingly, *S. guianense *has 190 ESTs coding for SVEP (14.4% of the transcripts of the S class), more than double those of *S. nigrimanum *(6.8% of its ESTs from the S class) and more than three times those of *S. vittatum *(which codes 4.5% of its ESTs from the S class to SVEP). These increases are highly significant, having scores of χ^2 ^= 16.72 (*p *= 2.3E^-8^) and χ^2 ^= 72.5 (*p *= 1.6E^-17^) to *S. nigrimanum *and *S. vittatum*, respectively. All sequences of SVEP proteins found in the sialotranscriptome of *S. guianense *were confirmed by proteome analysis within fraction 30, located just above the 14-kDa marker, consistent with the predicted (14 kDa) mature weight of SVEP protein (Figure 2 and Table 4).

##### *H-rich, acid proteins of *Simulium

**Figure 8:**
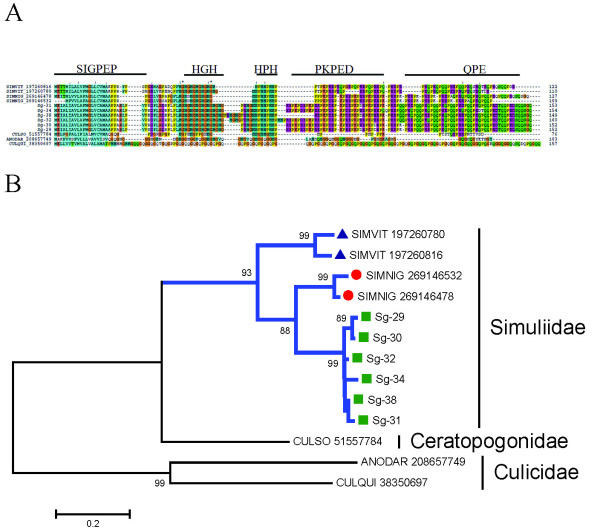
**The *Simulium *H-rich, acid protein family**. (A) Clustal alignment with their respective domains indicated as: (1) Signal peptide region, (2) HG repeat region, (3) HPH repeat region, (4) HKPED repeat region, and (5) QPE repeat region. The alignments of HP rich salivary proteins from *Culicoides sonorensis *(CulSO_), *Anopheles darlingi *(AD_) and *Culex quinquesfaciatus *(CQ_) were added with their accession numbers from the National Center for Biotechnology Information. The symbols above the alignment indicate (*) identical sites, (:) conserved sites, and (.) less-conserved sites. (B) Bootstrapped phylogram derived from the alignment in (A). *Simulium guianense *proteins are indicated by a square, *Simulium vittatum *by a triangle, and *Simulium nigrimanum *by a circle. The numbers on the tree bifurcations indicate the percentage bootstrap support above 75%. The bar at the bottom represents 20% amino acid substitution. Protein sequences were aligned by the Clustal program, and the dendogram was made with the Mega package after 10 000 bootstraps with the neighbor-joining algorithm. For other details, see Figure 3.

This protein family is known by its repeats of histidine, proline, glutamine, and glutamic acid residues. The repeat nature of these proteins had been suggested to interact with matrix proteins--possibly collagen--and function in a manner analogous to mosquito Aegyptins, which inhibit collagen-induced platelet aggregation [88]. It is also possible that the His repeats act as antimicrobials by chelating Zn or other trace element ions [125-127]. The black fly *S. vittatum *revealed this family to be the most abundant protein family expressed in its sialotranscriptome, with four repeat regions in its sequences (arginine-rich, HG repeat, HPH repeat, and QPE repeat) [14]. Similarly, mosquito and *Culicoides *sialotrancriptomes also contain proteins with Pro-His and Gly-His repeats, but no other sequence similarities. The *S. guianense *sialotranscriptome has 9.6% of all its secretory ESTs coding for members of this family, having above 70% identity to their homologous *S. nigrimanum *proteins. Alignment (Figure 8) shows that the *S. guianense *sequences, together with their homologous *S. nigrimanum *proteins, contain one repeat region coding for Pro-Lys-Pro residues, whereas in *S. vittatum*, the Lys residue is substituted by Gln. The phylogram of this protein family (Figure 8), when added to mosquito and *Culicoides *sequences, reveals that all *Simulium *sequences indicate (as expected) a common ancestor with 93% bootstrap support, with *S. guianense *sharing the same branch with *S. nigrimanum *(also as expected).

##### Mucins

**"**Mucins are low-complexity proteins rich in serine and threonine residues. They are frequently found in sialotranscriptomes of bloodsucking arthropods such as mosquitoes [72,73,128], biting midges [69], bed bugs [46] and black flies [14,15]. While these proteins' biologic function is not completely known, they have been suggested to provide protection to internal parts of the salivary ducts and also to have antimicrobial functions. They are commonly expressed in moist epithelia, where they offer protection [129,130] In addition, mucins are modified post-translationally, and their mature forms have N-acetyl-galactosamine residues [130]." Two types of *Simulium*-specific mucins are found in the sialotranscriptome of *S. guianense*, as follows.

##### Simulium *mucin family*

**Figure 9:**
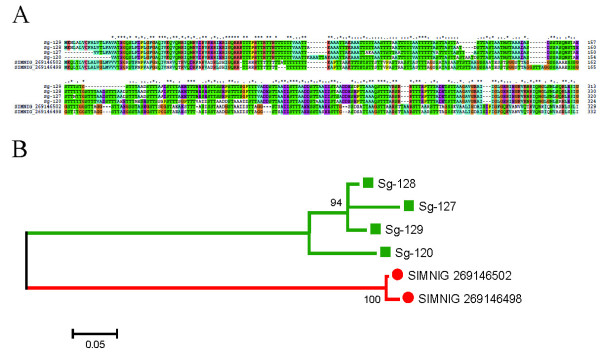
**The *Simulium *salivary mucin family**. (A) Clustal alignment. (B) Bootstrapped phylogram from the alignment in (A). The *Simulium guianense *proteins are indicated by a square and the *Simulium nigrimanum *homologs by a circle. The symbols above of the alignment indicate (*) identical sites, (:) conserved sites, and (.) less-conserved sites. The numbers on the tree bifurcations indicate the percentage bootstrap support above 75%. The bar at the bottom represents 5% amino acid substitution. Protein sequences were aligned by the Clustal program, and the dendogram was made with the Mega package after 10 000 bootstraps with the neighbor joining algorithm. For other details, see Figure 3.

Nine proteins (with 40 ESTs) in the *S. guianense *sialotranscriptome code for *Simulium *mucin. Their coding sequences have high amounts of Ser + Thr residues, varying from 34.6 to 42.6%, and from 40-144 galactosylation sites are predicted by the NetOglyc server [131]. Although similar sequences were found in the sialotranscriptome of *S. nigrimanum*, members of this family were absent in *S. vittatum*. Alignment of the *S. guianense *and *S. nigrimanum *sequences reveals extensive similarities and identities along the whole sequence, but the phylogram clearly distinguishes *S. guianense *and *S. nigrimanum *specific clades (Figure 9). The variation among the *S. guianense *sequences may result from splice variants, polymerase slippage on nucleotide repeats, or multiple genes. Several tryptic peptides were deduced by MS/MS with matches for *Simulium *mucin within fraction 16 (just below the 62-kDa marker) and to fraction 9 (just above the 96-kDa marker) (Figure 2 and Table 4).

##### *Acid mucins proteins similar to Basic 7-13 *Simulium *family*

**Figure 10:**
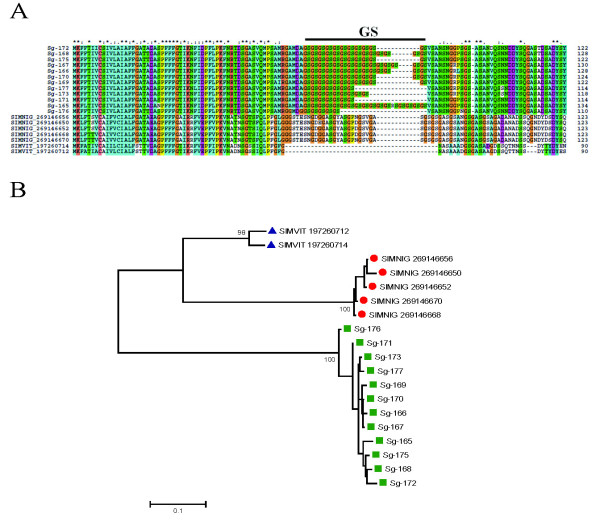
**Phylogenetic analysis of the acid mucin protein family**. (A) Clustal alignment. (B) Bootstrapped phylogram of the alignment in (A). The *Simulium guianense *sequences are indicate by a square, the *Simulium nigrimanum *proteins by a circle, and the *Simulium vittatum *sequences by a triangle. The symbols above of the alignment indicate (*) identical sites, (:) conserved sites, and (.) less-conserved sites. The numbers on the tree bifurcations indicate the percentage bootstrap support above 75%. The bar at the bottom represents 5% amino acid substitution. Protein sequences were aligned by the Clustal program, and the dendogram was made with the Mega package after 10 000 bootstraps with the neighbor-joining algorithm. For other details, see Figure 3.

The sialotranscriptome of *S. nigrimanum *reported small basic proteins (pI 8.1-10.6) with mature weight varying from 7 to 13 kDa and above 65% identity to orphan proteins found in the sialotranscriptome of *S. vittatum *[15]. The sialotranscriptome of *S. guianense *revealed 13 proteins (with 39 ESTs) with best matches to members of this family--from both *S. vittatum *and *S. nigrimanum*--at a 50% identity level; however, these sequences code for acid, not basic, proteins (pI 4.1-4.4) (Figure 10), with 0 to 19 potential galactosylation sites. Notably, the *S. guianense *proteins have an extended central domain containing Gly-Ser repeats that vary in size among the proteins, which may reflect polymerase slippage among closely related genes (Figure 10). The phylogenetic tree cluster produces monospecific branches indicative either of single polymorphic genes or, alternatively, of multiple genes that possibly interact, producing gene conversions (Figure 10).

##### Simulium *collagen-like family*

**Figure 11:**
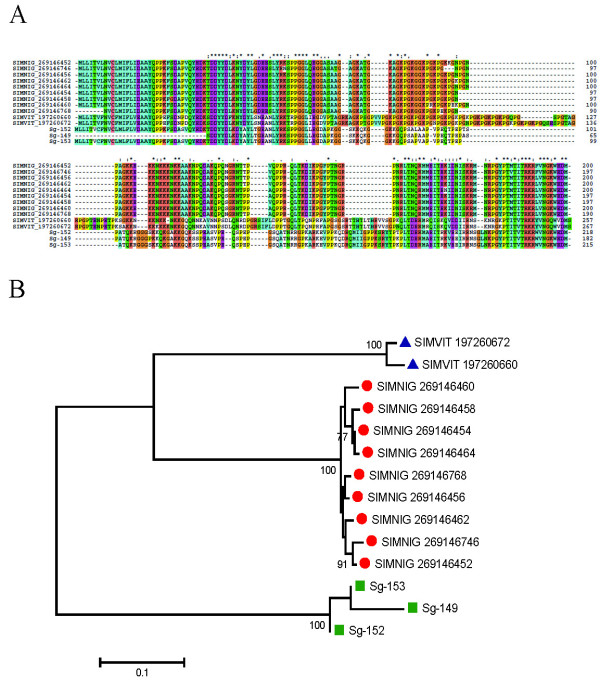
**Phylogenetic analysis of the *Simulium *collagen-like family**. (A) Clustal alignment. (B) Bootstrapped phylogram of the alignment in (A). The *Simulium guianense *proteins are indicated by a square, the *Simulium nigrimanum *proteins by a circle, and the *Simulium vittatum *proteins by a triangle. The symbols above of the alignment indicate (*) identical sites, (:) conserved sites, and (.) less-conserved sites. The numbers on the tree bifurcations indicate the percentage bootstrap support above 75%. The bar at the bottom represents 5% amino acid substitution. Protein sequences were aligned by the Clustal program, and the dendogram was made with the Mega package after 10 000 bootstraps with the neighbor-joining algorithm. For other details, see Figure 3.

Previous sialotranscriptomes of black flies reported specific proteins named as *Simulium *collagen-like that are rich in Pro-Gly residues [14,15]. Homologs to this family were found in the sialotranscriptome of *S. guianense*, with 17 clusters containing 48 ESTs (Table 2). These 17 clusters are variations of only three sequences, which were aligned with their *Simulium *homologs (having ~ 40% identity) to produce the phylogenetic tree shown in Figure 11. Alignment revealed relatively conserved AAs in the length of the sequence, with some gaps due to the longer sequences from *S. vittatum*. The phylogram maintains monospecific clades, as seen before for the mucin families (Figure 11). Several tryptic peptides were deducted by MS/MS within fraction 23, just above the 28-kDa standard, above the predicted (22 kDa) mature weight of these proteins (Figure 2 and Table 4).

##### Sv 7.8 kDa family

**Figure 12:**
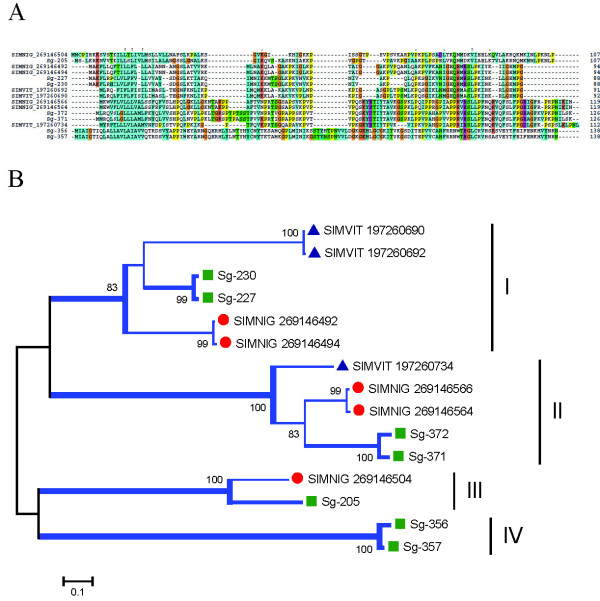
**The *Simulium *Sv 7.8-kDa protein family**. (A) Clustal alignment. (B) Bootstrapped phylogram of the alignment in (A). The *Simulium guianense *sequences are indicate by a square, *Simulium nigrimanum *proteins by a circle, and *Simulium vittatum *proteins by a triangle. The symbols above of the alignment indicate (*) identical sites, (:) conserved sites, and (.) less-conserved sites. The numbers on the tree bifurcations indicate the percentage bootstrap support above 75%. The bar at the bottom represents 5% amino acid substitution. Protein sequences were aligned by the Clustal program, and the dendogram was made with the Mega package after 10 000 bootstraps with the neighbor-joining algorithm. For other details, see Figure 3.

Members of this family were first found in the sialotranscriptome of *S. vittatum *coding for proteins with 7.8 kDa mol wt [14]. Later, sialotranscriptome of *S. nigrimanum *added six more transcripts to this family, suggesting it to be a divergent multifamily gene from *Simulium *[15]. Sequences from *S. guianense *maintain 60% identity to its homologous *S. nigrimanum *protein and 50% to the *S. vittatum *protein, coding for basic proteins (pI 9-10.4) with mature weight varying from 7.1 to 13.4 kDa. Alignment revealed low levels of conserved AAs and at least four genes to *S. guianense *proteins of this family, marked as clades I-IV on Figure 12. Several tryptic peptides were deducted by MS/MS in the fractions F27, F30, F32, and F31. These fractions are located in the gel just above the 14-kDa marker and just above the 3-kDa marker. These results are consistent with the predicted (7 to 13 kDa) mature weight of these proteins (Figure 2 and Table 4).

##### *Basic 7-13 *Simulium *family*

The *S. guianense *sialotranscriptome added two more proteins (Sg-420 and Sg-403) with six ESTs to this family coding to basic proteins (pI 11) and 8 kDa, with more than 59% similarities to their homologous *S. nigrimanum *and *S. vittatum *proteins. Tryptic peptides were found by MS/MS within fractions 30 and 31, just below the 6-kDa standard (Figure 2 and Table 4). Their function remains unknown.

##### Simulium *4.8-kDa family*

Five more transcripts were added to this family, which appears highly conserved in sialotranscriptomes of *Simulium*. Their sequences code to acidic proteins (pI 4.4) with 5 kDa of mature weight and are devoid of cysteines. These peptides have unknown function.

##### Simulium *Basic 7.4-kDa family*

The cluster Sg-422 (with four ESTs) codes to a basic peptide of 7 kDa mol wt and above 50% identity to their homologous proteins from *S. vittatum *and *S. nigrimanum*. This protein family also does not contain any Cys residues on the mature peptide. Four tryptic peptides originated from Sg-422 were deduced by MS/MS within fraction 31, just below the 6-kDa standard, consistent with the predicted (6.8 kDa) mature weight of this protein (Figure 2 and Table 4).

##### Simulium *Basic 13-kDa*

Sg-446 added three more ESTs to this family, and has ~ 40% identity to other *Simulium *proteins. They do not match other known proteins in any of the NR, GO, KOG, CDD, PFAM, or SMART databases. Two tryptic peptides were deduced by MS/MS within fraction 29, just below the 14-kDa standard and consistent with the predicted (13.7 kDa) mature weight of this protein (Figure 2 and Table 4).

##### *5-Cys *Simulium *family*

This family received this name because it contains five Cys in their sequences. One protein with nine ESTs is here reported in the *S. guianense *sialotranscriptome coding to an acid protein (pI 5.7) with 14-kDa mol wt and above 60% of identity to homologs found in *Simulium *sialotranscriptomes. Three tryptic peptides were deduced by MS/MS within fraction F30, just below the 6-kDa standard (Figure 2 and Table 4).

*Families deorphanized from *S. nigrimanum. Six additional protein families were characterized in common between *S. nigrimanum *and *S. guianense*, and no other known protein. They do not produce significant matches to others proteins in the NR database, and have thus deorphanized these *S. nigrimanum *proteins.

*Deorphanized *S. nigrimanum *8-10 Cys W family*. This family is so named because their members contain from 8 to 10 conserved Cys and Trp in their mature sequences. The sialotranscriptome of *S. nigrimanum *revealed two distinct subfamilies, one containing 10 Cys and 5 conserved Trp and other containing 8 Cys and 6 Trp. This last group was suggested as a candidate protein in the etiology of pemphigus foliaceus due its similarity to proteins annotated as junctional adhesion molecules [15]. The *S. guianense *sialotranscriptome added two more proteins to this family (11 ESTs), which contain 9 Cys and 5 or 6 Trp. These proteins were confirmed by MS/MS within fraction 29, just below the 14-kDa standard, near their predicted (16.9 kDa) mature weights (Figure 2 and Table 4). The function of this protein family remains unknown.

The sialotrancriptome of *S. guianense *added three more proteins (Sg-319, Sg-320, and Sg-321) with 12 ESTs to the *Acid 28-kDa family*. PAGE MS/MS results reported many tryptic peptides for these proteins within fraction F23, just above the 28-kDa marker (Figure 2 and Table 4), in accordance with the predicted (22 to 27 kDa) mature mol wt of these proteins. One protein (Sg-136) with nine ESTs was added to *Simulium Basic 28-kDa family*. This protein (Sg-136) had several tryptic peptides deducted by MS/MS within fraction 24, consistent with a mass near 28 kDa (Figure 2 and Table 4). The protein family named as *19-kDa family*, first seen in *S. nigrimanum*, was deorphanized with two proteins (Sg-303 and Sg-309) with 10 ESTs coding for basic proteins of 16.8 MW and signal peptide in their sequences. Tryptic peptides were found by MS/MS within the fraction 27, located just above the 14-kDa standard and consistent with the predicted (16.8 kDa) mature weight of this protein (Figure 2 and Table 4). Other putative secreted peptides were also deducted from the *S. guianense *sialotranscriptome, such as the cluster Sg-258 (22 ETS) coding to basic protein of 8-kDa mol wt that has 70% identity to the orphan protein of the *S. nigrimanum *sialotranscriptome previously named *8-kDa basic protein family*. Five tryptic peptides were deducted by MS/MS within fraction 31, just below the 6-kDa marker (Figure 2 and Table 4). The smaller peptide found in this cDNA library also represents one case of deorphanization with two ESTs in cluster Sg-438 matching members of the *Sn basic 4.4-kDa family*.

#### Proteins currently unique to *S. guianense*

##### Novel peptide similar to kunitoxin

The *S. guianense *has two clusters (Sg-375 and Sg-409) coding to novel peptide distantly similar (32% identity) to the snake peptide kunitoxin [132]. They are Cys-rich and were suggested as protease and serine protease inhibitors in snake venom glands [133]. Although the snake peptides have a typical Kunitz domain, this domain is not identified in the black fly protein. Kunitoxin inhibits plasmin and thrombin, blocks L-type calcium channels, and forms part of the neurotoxic complexes with PLA_2 _molecules [133]. No similar sequences have been found so far in any previously described sialotranscriptome. Together, the black fly family grouped eight ESTs coding for this secreted basic peptide with 8-9 kDa. The PAGE/MS/MS run reported four tryptic peptides for the Kunitoxin-like protein at fraction 31, coincident with a well-stained band between 3 and 6-kDa standards (Figure 2 and Table 4).

## Conclusions

Sialotranscriptomes of hematophagous insects have revealed a large number of putative novel proteins, helping to understand the role of saliva in blood feeding, sugar feeding, and transmission of distinct parasites. In the last 2 years, two black fly sialotrancriptomes were described. The sialome of *S. guianense *represented the first from a species with confirmed vectorial status for onchocerciasis. Black flies had their origin ~180 MYA (Middle Jurassic), based on the fossil record [58], and currently are among the best studied Diptera, with 2,025 species named, 12 of which are fossil [57]. Their blood feeding mode has been proposed as a plesiomorphic character in the Culicomorpha appearing during the Triassic ~250 MYA and diverging in the Late Jurassic. Based on tectonic plate movement, we believe that Neotropical black flies share a distant common origin with Neartic species, because union of the Americas only occurred during the Cenozoic, after the irradiation of mammals. Thus, it is probable that this common black fly ancestor originated before the irradiation and expansion of mammals 60 MYA and probably had birds or reptiles as their blood source, and this origin has indeed been maintained in some species; however, others could have diverged to feeding on mammals, including humans, conferring a level of plasticity (zoophilic or anthropophilic behavior) inside the Simulidae family. For example, *S. nigrimanum *was found to have both feeding behaviors in different places. Conversely, *S. guianense *has a high degree of anthropophily and was incriminated as the main vector of river blindness in the focus that includes Brazil and Venezuela (Yanomami Indians) [4]. This plasticity seen in the choice of host could be accompanied by gene duplications and fast evolution in several protein families.

Here, we performed a phylogenetic analysis of protein families found in the sialomes of three black flies from different subgenera: *S. vittatum *(Neartic, zoophilic, autogenous, and non-vector of onchocerciasis), *S. nigrimanum *(Neotropical, zoophilic and anthropophilic, anautogenous, and potential vector) and *S. guianense *(Neotropical, anthropophilic, anautogenous, and vector of onchocerciasis). Notice that the last two are more closely overlapping in their characteristics. It is also important here to clear the taxonomic status of these species, mainly because *S. nigrimanum *shares the same geographic distribution as *S. guianense*, except for *S. nigrimanum *absence within the Amazon region. Currently, some authors [134] group both species into the *Trichodagmia *subgenus of *Simulium*, while--based on phylogenetic analysis--others have determined [135] that *S. guianense *belong to a different subgenus, *Thyrsopelma*, and elevated the subgenus to genus (thus *Trichodagmia nigrimanum *and *Thyrsopelma guianense*, which are cited in this work as *S. nigrimanum *and *S. guianense*, respectively).

Independent of this taxonomic confusion, it is clear from the phylogenetic analysis containing the black fly species that, in the majority of cases, proteins from *S. nigrimanum *grouped with strong bootstrap support with those of *S. guianense *while excluding from the same sub clade the *S. vittatum *homologs, an expected result from the biogeography of the species. On the other hand, the number of families that were found exclusive of Neotropical flies is entirely shared except for the *S. guianense *Kunitoxin family, suggesting a relatively recent common ancestor between these South American flies.

It is important to note the increased expression in *S. guianense *of some proteins families such as D7, SVEP, and other protein families specific to *Simulium *(which contain 32% of all transcripts), suggesting it to be associated with the anthropophilic and vectorial status of *S. guianense *in the transmission of onchocerciasis. Indeed, the autogenous *S. vittatum *has the least expression of salivary secreted proteins and lacks many of the families found in the Neotropical flies. *S. nigrimanum *was recently suggested as a potential vector of onchocerciasis [136].

From a conservative perspective, we confirmed the presence of ubiquitous salivary protein families such as Antigen-5, Yellow, ML domain, lipocalin, lysozyme, cecropin, serpin, Kunitz domain, serine protease, hyaluronidase, apyrase, glycosidase, ADA, and destabilase within the *Simulium *genus; however, four of these protein families (ML domain, serpin, hyaluronidase, and ADA) were exclusive to the *S. guianense *sialotranscriptome. Kunitz-domain proteins were seen in all black fly sialotranscriptomes. Probably this family is responsible for the anticoagulant activity previously related to SGHs in *S. guianense *[137].

Insect-specific protein families such as Aegyptin, D7 family (which include D7 ultra-short, D7 16-kDa, and long D7), and Diptera secreted protein from conserved insect family and were found in all black fly sialotranscriptomes. The protein laminin-like was found only in *S. guianense *and *S. vittatum *sialotranscriptomes.

As expected, *S. guianense *contained several protein families previously found only in the sialotranscriptomes of *S. nigrimanum *and also *S. vittatum *such as the SVEP, H-rich acid proteins, acid mucin proteins similar to basic 7-13 *Simulium *family, *Simulium *collagen-like, Sv 7.8-kDa family, 5-Cys *Simulium *family, basic 7-13 *Simulium *family, *Simulium *4.8-kDa family, *Simulium *basic 7.4-kDa family, and *Simulium *basic 13-kDa family. Except for SVEP, a vasodilator, none of these proteins' function is known. It is possible that some of these families share the same function. Additionally, the *S. guianense *sialotranscriptome revealed protein families previously found exclusive to *S. nigrimanum *such as the *Simulium *mucin, 28-kDa basic *Simulium *family, acid 28-kDa family, 19-kDa family, Sn 8-10 Cys W family, 8-kDa basic protein, and Sn basic 4.4-kDa family, none of which have a known function. We also identified proteins currently unique to *S. guianense *such as a novel peptide similar to kunitoxin commonly found in venom of snakes. Transcripts associated with sugar feeding, such as glycosidases, show a common ancestor in the Diptera (fruit flies and mosquito); however, immune-related products such as trypsins appear phylogeneticaly more expanded relative to dipterous and non-dipterous insects such as lepidopterans. In mosquitoes, trypsin activity was suggested as the first line of defense against microorganisms during feeding [101]. *S. guianense *also has this activity confirmed by in-gel protein digestion assays from SGHs (data not published) and possibly could conserve the same function in black flies. Finally, our results contribute to understanding the role of *Simulium *saliva in the transmission of *O. volvulus *and in the evolution of the salivary proteins in black flies. It also consists of a platform for mining novel antihemostatic compounds, epidemiologic markers of vector exposure, and vaccine candidates against filariasis.

## Methods

### Chemicals

Standard laboratory chemicals were purchased from Sigma Chemicals (St. Louis, MO) if not specified otherwise.

### Black Flies

Female adult *S. guianense *were obtained from pupae collected in waterfalls with aquatic plants of the Podostemaceae family. The breeding sites are located in the Jauaperi River, Rorainopolis municipality, Roraima state, Brazil. Identification of the black fly species followed the standard keys from Shelley et al.[138].

Insects were kept with free access to 10% Karo^® ^honey diluted solution. SGs from recently emerged and 1- to-2-day-old adult female (25 of each age) were dissected in 150 mM sodium chloride pH 7.4, immediately transferred to 50-μL of RNAlater (Ambion, Inc., Austin, TX), and kept refrigerated until use.

### Library Construction

SG RNA, extracted from 75 intact glands, was isolated using the Micro-FastTrack mRNA isolation kit (Invitrogen, San Diego, CA). Other procedures were as described before [14,15] and are reproduced here for easiness of access to the reader: "The PCR-based cDNA library was made following the instructions for the SMART (switching mechanism at 5'end of RNA transcript) cDNA library construction kit (Clontech, Palo Alto, CA). This system uses oligoribonucleotide (SMART IV) to attach an identical sequence at the 5' end of each reverse-transcribed cDNA strand. This sequence is then utilized in subsequent PCR reactions and restriction digests.

First-strand synthesis was carried out using PowerScript reverse transcriptase at 42°C for 1 h in the presence of the SMART IV and CDS III (3') primers. Second-strand synthesis was performed by a long-distance PCR-based protocol using Advantage Taq polymerase (Clontech) mix in the presence of the 5' PCR primer and the CDS III (3') primer. The cDNA synthesis procedure resulted in creation of *SfiI A *and *B *restriction enzyme sites at the ends of the PCR products that are used for cloning into the phage vector (λ TriplEx2 vector; Clontech). PCR conditions were as follows: 95°C for 1 min; 26 cycles of 95°C for 15 sec, 68°C for 6 min. A small portion of the cDNA obtained by PCR was analyzed on an E-Gel^® ^1.2% with SYBR Safe (Invitrogen) to check quality and range of cDNA synthesized. Double-stranded cDNA was immediately treated with proteinase K (0.8 μg/mL) at 45°C for 20 min, and the enzyme was removed by ultrafiltration though a Microcon YM-100 centrifugal filter device (Amicon Inc., Beverly, CA). The cleaned, double-stranded cDNA was then digested with *SfiI *at 50°C for 2 h, followed by size fractionation on a ChromaSpin-400 column (Clontech) into small (S), medium (M), and large (L) transcripts based on their electrophoresis profile on an E-Gel^® ^1.2% with SYBR Safe. Selected fractions were pooled and concentrated using a Microcon YM-100.

The concentrated cDNA mixture was ligated into the λ TriplEx2 vector, and the resulting ligation mixture was packaged using the GigaPack^® ^III Plus packaging extract (Stratagene, La Jolla, CA) according to the manufacturer's instructions. The packaged library was plated by infecting log-phase XL1-Blue *Escherichia coli *cells (Clontech). The percentage of recombinant clones was determined by blue-white selection screening on LB/MgSO_4 _plates containing X-gal/IPTG. Recombinants were also determined by PCR, using vector primers PT2F1 (AAG TAC TCT AGC AAT TGT GAG C) and PT2R1 (CTC TTC GCT ATT ACG CCA GCT G) flanking the inserted cDNA, with subsequent visualization of the products on an E-Gel^® ^1.2% with SYBR Safe."

### cDNA Sequencing

This was done as described before [14,15] and is reproduced here for easiness of access to the reader:**"**Twenty 96-well plates were prepared for cyclo sequencing, each containing 94 clones and two DNA controls, as follows: The cDNA library was plated on LB/MgSO_4 _plates containing X-gal/IPTG to an average of 250 plaques per 150 mm Petri plate. Recombinant (white) plaques were randomly selected and transferred to 96-well microtiter plates (Nunc, Rochester, NY) containing 75 μL of ultrapure water (KD Medical, Columbia, MD) per well. The plates were covered and placed on a gyrating shaker for 30 min at room temperature. The phage suspension was either immediately used for PCR or stored at 4°C for future use.

To amplify the cDNA using a PCR reaction, 5 μL of the phage sample was used as a template. The primers were sequences from the λ TriplEx2 vector and named PT2F1 (AAG TAC TCT AGC AAT TGT GAG C) and PT2R1 (CTC TTC GCT ATT ACG CCA GCT G), positioned at the 5' end and the 3' end of the cDNA insert, respectively. The reaction was carried out in a 96-well PCR microtiter plate (Applied Biosystems, Inc., Foster City, CA) using FastStart Taq polymerase (Roche Diagnostics, Mannheim, Germany) on a GeneAmp PCR system 9700 (Perkin Elmer Corp., Foster City, CA). The PCR conditions were 1 hold of 75°C for 3 min; 1 hold of 94°C for 4 min, 30 cycles of 94°C for 1 min, 49°C for 1 min; 72°C for 4 min. The amplified products were analysed on an E-Gel^® ^1.2% with SYBR Safe. Clones were PCR amplified, and those showing a single band were selected for sequencing. Approximately 200-250 ng of each PCR product was transferred to a 96-well PCR microtiter plate (Applied Biosystems) and frozen at -20°C. Samples were shipped on dry ice to the Rocky Mountain Laboratories Genomics Unit (NIAID, NIH, Hamilton, MT) with primer (PT2F3: TCT CGG GAA GCG CGC CAT TGT) and template combined together in a 96-well optical reaction plate (P/N 4306737; Applied Biosystems) following the manufacturer's recommended concentrations. Sequencing reactions were set up as recommended by Applied Biosystems' BigDye^® ^Terminator v3.1 cycle sequencing kit by adding 1 μL ABI BigDye^® ^Terminator ready reaction mix v3.1 (P/N 4336921), 1.5 μL 5x ABI sequencing buffer (P/N 4336699), and 3.5 μL of water for a final volume of 10 μL. Cycle sequencing was performed at 96°C for 10 sec, 50°C for 5 sec, 60°C for 4 min for 27 cycles on either a Bio-Rad Tetrad 2 (Bio-Rad Laboratories, Hercules, CA) or ABI 9700 thermal cycler (Applied Biosystems). Fluorescently labeled extension products were purified following Applied Biosystems' BigDye^® ^XTerminator™ purification protocol and subsequently processed on an ABI 3730xL DNA Analyzer (Applied Biosystems)."

The EST sequences described in this article were deposited in NCBI's DBEST database under accessions HS415024 - HS416811. Coding sequences and their protein translations were submitted to GenBank under accessions JI626169-JI626342.

### Bioinformatic Tools and Procedures

This was done as described before [14,15] and is reproduced here for easiness of access to the reader: **"**Expressed sequence tags (EST) were trimmed of primer and vector sequences. The BLAST tool [59], CAP3 assembler [139] and ClustalW [140] software were used to compare, assemble, and align sequences, respectively. Phylogenetic analysis and statistical neighbor-joining bootstrap tests of the phylogenies were done with the Mega package [141]. For functional annotation of the transcripts, we used the tool blastx [59] to compare the nucleotide sequences to the NR protein database of the NCBI and to the Gene Ontology (GO) database [60]. The tool, reverse position-specific BLAST (rpsblast)[59] was used to search for conserved protein domains in the Pfam [142], SMART [143], Kog [144] and conserved domains databases (CDD) [61]. We also compared the transcripts with other subsets of mitochondrial and rRNA nucleotide sequences downloaded from NCBI. Segments of the three-frame translations of the ESTs (because the libraries were unidirectional, six-frame translations were not used), starting with a methionine found in the first 300 predicted amino acids (AAs), or the predicted protein translation in the case of complete coding sequences, were submitted to the SignalP server [62] to help identify translation products that could be secreted. O-glycosylation sites on the proteins were predicted with the program NetOGlyc [131]. Functional annotation of the transcripts was based on all the comparisons above. Following inspection of all these results, transcripts were classified as either secretory (S), housekeeping (H), or of unknown (U) function, with further subdivisions based on function and/or protein families. Putative sequences deriving from transposable elements (TE) were also found."

### Proteomic Characterization Using One-Dimensional Gel Electrophoresis and Tandem Mass Spectrometry (MS/MS)

The soluble protein fraction from SGHs from *S. guianense *corresponding to approximately 50 μg of protein was brought up in reducing Laemmli gel-loading buffer. The sample was boiled for 10 min and resolved on a NuPAGE 4-12% Bis-Tris precast gel. The separated proteins were visualized by staining with SimplyBlue (Invitrogen). The gel was sliced into 32 individual sections that were destained and digested overnight with trypsin at 37°C. Peptides were extracted and desalted using ZipTips (Millipore, Bedford, MA) and resuspended in 0.1% TFA prior to S analysis.

Nanoflow reversed-phase liquid chromatography tandem MS (RPLS-MS/MS) was performed using an Agilent 1100 nanoflow LC system (Agilent Technologies, Palo Alto, CA) coupled online with a linear ion-trap (LIT) mass spectrometer (LTQ, ThermoElectron, San José, CA). This was done as described before [14,15] and is reproduced here for easiness of access to the reader: "NanoRPLC columns were slurry-packed in-house with 5 μm, 300-Å pore size C-18 phage (Jupiter, Phenomenex, CA) in a 75-μm i.d. × 10-cm fused silica capillary (Polymicro Technologies, Phoenix, AZ) with a flame-pulled tip. After sample injection, the column was washed for 30 min with 98% mobile phase A (0.1% formic acid in water) at 0.5 μL/min, and peptides were eluted using a linear gradient of 2% mobile phase B (0.1% formic acid in acetonitrile) to 42% mobile phase B in 40 min at 0.25 μL/min, then to 98% B for an additional 10 min. The liner ion-trap mass spectrometer was operated in a data-dependent MS/MS mode in which each full MS scan was followed by seven MS/MS scans where the seven most abundant molecular ions were dynamically selected for collision-induced dissociation using a normalized collision energy of 35%. Dynamic exclusion was applied to minimize repeated selection of peptides previously selected for collision-induced dissociation.

Tandem mass spectra were searched using SEQUEST on a 20-node Beowulf cluster against an *S. guianense *proteome database with methionine oxidation included as dynamic modification. Only tryptic peptides with up to two missed cleavage sites meeting a specific SEQUEST scoring criteria [delta correlation (Δ*C*_n_) ≥ 0.08 and charge-state-dependent cross correlation (*X*_corr_) ≥ 1.9 for [M + H]^1+^, ≥ 2.2 for [M + 2H]^2+^, and ≥ 3.5 for [M + 3H]^3+^] were considered as legitimate identifications. The peptides identified by MS were converted to Prosite block format [145] by a program written in Visual Basic. This database was used to search matches in the Fasta-formatted database of salivary proteins, using the poorly documented program Seedtop, which is part of the BLAST package. The result of the Seedtop search is piped into the hyperlinked spreadsheet to produce a text file, such as the one shown for the apyrase proteins SV-2008. Notice that the ID lines indicate, for example, BF18_73, which means that one match was found for fragment number 73 from gel band 18. Because the same tryptic fragment can be found in many gel bands, another program was written to count the number of fragments for each gel band, displaying a summarized result in an Excel table. The summary in this form of BF11→18| BF12→18| BF13→2| indicates that 18 fragments were found in band 11, while 18 and 2 peptides were found in bands 12 and 13, respectively. Furthermore, this summary included protein identification only when two or more peptide matches to the protein were obtained from the same gel slice."

## Authors' contributions

All authors contributed to experimental design, data analysis and writing of the manuscript. ACC additionally contributed to insect collections, dissections, library construction and sequencing. EC supervised library construction. JMCR contributed to bioinformatic analysis. All authors read and approved the final manuscript.

## Supplementary Material

Additional file 1 **Hyperlinked Excel file with assembled contigs**. Can be downloaded from http://exon.niaid.nih.gov/transcriptome/S_guianense/S1/S_g-sup1-Web.xlsx. Click here for file

Additional file 2 Hyperlinked Excel file with coding sequence information, can be downloaded from http://exon.niaid.nih.gov/transcriptome/S_guianense/S2/S_g-S2-Web.xlsx. Click here for file

Additional file 3 **PDF file with Phi-blast results**. Click here for file
