# Supplementary material for: An insight into the sialome of Simulium guianense (DIPTERA:SIMulIIDAE), the main vector of River Blindness Disease in Brazil
Source: BMC Genomics. 2011 Dec 19;12:612. doi: 10.1186/1471-2164-12-612 (PMC3285218; doi:10.1186/1471-2164-12-612)
Supplement: Additional file 1 — Hyperlinked Excel file with assembled contigs. Can be downloaded from http://exon.niaid.nih.gov/transcriptome/S_guianense/S1/S_g-sup1-Web.xlsx. [file 1471-2164-12-612-S1.DOC]

**Additional file 1 - Supplemental file S1**

Hyperlinked Excel file with assembled contigs. Can be downloaded from

<http://exon.niaid.nih.gov/transcriptome/S_guianense/S1/S_g-sup1-Web.xlsx>
