# Supplementary material for: An insight into the sialome of Simulium guianense (DIPTERA:SIMulIIDAE), the main vector of River Blindness Disease in Brazil
Source: BMC Genomics. 2011 Dec 19;12:612. doi: 10.1186/1471-2164-12-612 (PMC3285218; doi:10.1186/1471-2164-12-612)
Supplement: Additional file 2 — Hyperlinked Excel file with coding sequence information, can be downloaded from http://exon.niaid.nih.gov/transcriptome/S_guianense/S2/S_g-S2-Web.xlsx. [file 1471-2164-12-612-S2.DOC]

**Additional file 2 - Supplemental file S2**

Hyperlinked Excel file with coding sequence information, can be downloaded from

<http://exon.niaid.nih.gov/transcriptome/S_guianense/S2/S_g-S2-Web.xlsx>
