# Supplementary material for: An insight into the sialome of Simulium guianense (DIPTERA:SIMulIIDAE), the main vector of River Blindness Disease in Brazil
Source: BMC Genomics. 2011 Dec 19;12:612. doi: 10.1186/1471-2164-12-612 (PMC3285218; doi:10.1186/1471-2164-12-612)
Supplement: Additional file 3 — PDF file with Phi-blast results. [file 1471-2164-12-612-S3.PDF]

BLAST

Basic Local Alignment Search Tool

NCBI/ BLAST/ blastp suite/ **Formatting Results - MSD9UF0J014**

PSI blast Iteration 5

Sg-216 hypothetical salivary simulium-specific...

Skip to the first new sequence

**Query ID**	lcl|29951	**Database Name**	nr
**Description**	Sg-216 hypothetical salivary simulium-specific secreted protein [Simulium guianensis]	**Description**	All non-redundant GenBank CDS translations+PDB+SwissProt+PIF excluding environmental samples from WGS projects
**Molecule type**	amino acid	**Program**	BLASTP 2.2.25+
**Query Length**	363		

Graphic Summary

Distribution of 146 Blast Hits on the Query Sequence

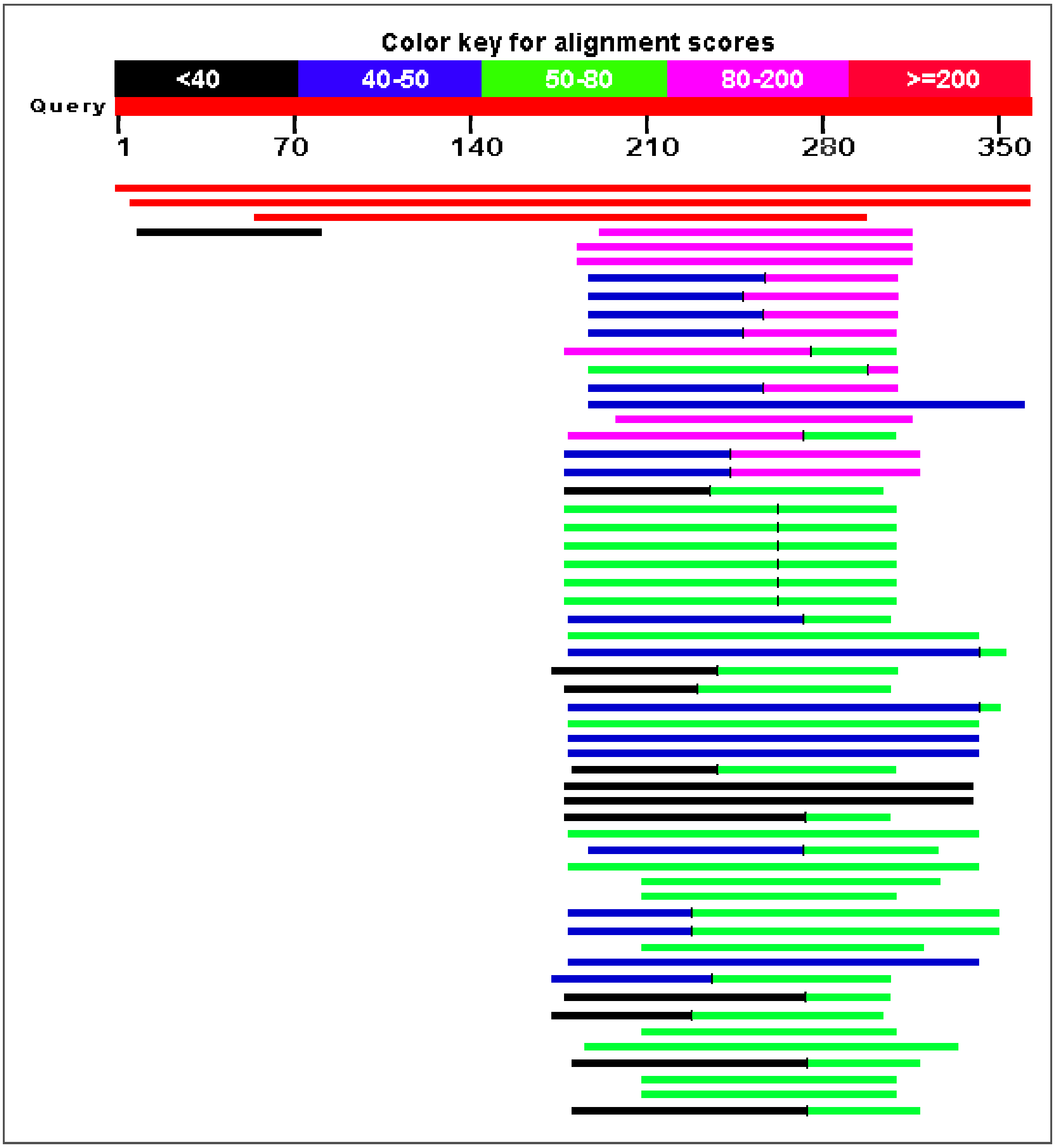

Descriptions

Legend for links to other resources: U UniGene E GEO G Gene S Structure M Map Viewer PubChem BioAssay

Images legend
NEW - alignment score below the threshold on the previous iteration
- alignment was checked on the previous iteration

Sequences producing significant alignments with pattern at position and E-value BETTER than threshold

Accession	Description	Max score	Total score	Query coverage	E value	Links
NEW ACZ28230.1	hypothetical secreted protein [Simulium nigrimanum]	605	605	99%	3e-171	
NEW ACZ28349.1	hypothetical conserved protein [Simulium nigrimanum]	589	589	98%	2e-166	
NEW ACZ28321.1	hypothetical protein [Simulium nigrimanum]	410	410	66%	2e-112	
NEW ACZ28152.1	hypothetical conserved protein [Simulium nigrimanum]	130	130	34%	2e-28	
NEW ACZ28153.1	hypothetical protein [Simulium nigrimanum]	129	129	36%	6e-28	
NEW ACZ28354.1	hypothetical protein [Simulium nigrimanum]	129	129	36%	8e-28	
NEW XP_001851110.1	conserved hypothetical protein [Culex quinquefasciatus] >gb|EDS33063.1| conserved hypothetical protein [Culex quinquefasciatus]	123	123	29%	4e-26	UG
NEW XP_001851111.1	conserved hypothetical protein [Culex quinquefasciatus] >gb|EDS33064.1| conserved hypothetical protein [Culex quinquefasciatus]	113	113	28%	5e-23	G
NEW XP_001851115.1	conserved hypothetical protein [Culex quinquefasciatus] >gb|EDS33068.1| conserved hypothetical protein [Culex quinquefasciatus]	107	107	28%	2e-21	UG
NEW XP_001851109.1	conserved hypothetical protein [Culex quinquefasciatus] >gb|EDS33062.1| conserved hypothetical protein [Culex quinquefasciatus]	107	107	27%	3e-21	UG
NEW XP_001968326.1	GG24571 [Drosophila erecta] >gb|EDV57385.1| GG24571 [Drosophila erecta]	101	101	26%	1e-19	G
NEW XP_001648416.1	hypothetical protein AaeL_AAEL014309 [Aedes aegypti] >gb|EAT33416.1| conserved hypothetical protein [Aedes aegypti]	90.2	90.2	28%	4e-16	UG
NEW XP_001648417.1	hypothetical protein AaeL_AAEL014309 [Aedes aegypti] >gb|EAT33417.1| conserved hypothetical protein [Aedes aegypti]	90.2	90.2	28%	4e-16	UG
NEW EFR25576.1	hypothetical protein AND_08977 [Anopheles darlingi]	89.8	89.8	28%	6e-16	
NEW ACH56909.1	hypothetical protein [Simulium vittatum]	89.0	89.0	32%	9e-16	
NEW XP_002087581.1	GE15356 [Drosophila yakuba] >gb|EDW87293.1| GE15356 [Drosophila yakuba]	81.7	81.7	25%	1e-13	G
NEW EFA08554.1	hypothetical protein TcasGA2_TC006209 [Tribolium castaneum]	80.9	80.9	30%	3e-13	
NEW XP_969643.2	PREDICTED: similar to GA15301-PA [Tribolium castaneum]	80.9	80.9	30%	3e-13	UGM
NEW XP_310721.4	AGAP000385-PA [Anopheles gambiae str. PEST] >gb|EAA06714.4| AGAP000385-PA [Anopheles gambiae str. PEST]	77.8	77.8	25%	2e-12	UG
NEW NP_722735.2	CG31664 [Drosophila melanogaster] >gb|AAF51336.3| CG31664 [Drosophila melanogaster]	77.4	77.4	23%	3e-12	
NEW ACH95281.1	FI07603p [Drosophila melanogaster]	77.4	77.4	23%	3e-12	
NEW AAY55318.1	IP12601p [Drosophila melanogaster]	77.4	77.4	23%	3e-12	
NEW AAY55307.1	IP12501p [Drosophila melanogaster]	77.4	77.4	23%	3e-12	
NEW XP_002077791.1	GD22890 [Drosophila simulans] >gb|EDX03376.1| GD22890 [Drosophila simulans]	76.3	76.3	23%	6e-12	UG
NEW XP_002077790.1	GD22891 [Drosophila simulans] >gb|EDX03375.1| GD22891 [Drosophila simulans]	76.3	76.3	23%	6e-12	UG
NEW XP_001121007.1	PREDICTED: similar to lethal (1) G0193 CG2206-PB, isoform B [Apis mellifera]	74.4	74.4	33%	2e-11	UGM
NEW XP_002067370.1	GK16218 [Drosophila willistoni] >gb|EDW78356.1| GK16218 [Drosophila willistoni]	73.6	73.6	27%	4e-11	G
NEW XP_002076900.1	GD24578 [Drosophila simulans] >gb|EDX16494.1| GD24578 [Drosophila simulans]	71.7	71.7	39%	2e-10	UG
NEW XP_002427759.1	conserved hypothetical protein [Pediculus humanus corporis] >gb|EEB15021.1| conserved hypothetical protein [Pediculus humanus corporis]	71.3	71.3	28%	2e-10	G
NEW EFN73534.1	hypothetical protein EAG_09552 [Camponotus floridanus]	70.9	70.9	27%	3e-10	
NEW XP_001978593.1	GG17594 [Drosophila erecta] >gb|EDV47520.1| GG17594 [Drosophila erecta]	70.9	70.9	39%	3e-10	G
NEW XP_001963941.1	GF20993 [Drosophila ananassae] >gb|EDV34390.1| GF20993 [Drosophila ananassae]	70.5	70.5	27%	3e-10	G
NEW NP_996366.1	lethal (1) G0193, isoform B [Drosophila melanogaster] >gb|AAS65275.1| lethal (1) G0193, isoform B [Drosophila melanogaster]	70.5	70.5	27%	3e-10	UG
NEW NP_572427.1	lethal (1) G0193, isoform A [Drosophila melanogaster] >gb|AAF46301.1| lethal (1) G0193, isoform A [Drosophila melanogaster] >gb|AAL13770.1| LD24308p [Drosophila melanogaster] >gb|ACL84971.1| l(1)G0193-PA [synthetic construct]	70.5	70.5	27%	3e-10	UG
NEW EFN89744.1	hypothetical protein EAI_01992 [Harpegnathos saltator]	70.1	70.1	33%	5e-10	
NEW EFA12872.1	hypothetical protein TcasGA2_TC010724 [Tribolium castaneum]	69.7	69.7	33%	6e-10	
NEW XP_967785.2	PREDICTED: similar to conserved hypothetical protein [Tribolium castaneum]	69.7	69.7	33%	6e-10	GM
NEW XP_001810077.1	PREDICTED: similar to GA15301-PA [Tribolium castaneum]	69.0	69.0	33%	1e-09	UGM
NEW XP_002101210.1	GE17494 [Drosophila yakuba] >gb|EDX02318.1| GE17494 [Drosophila yakuba]	68.6	68.6	27%	1e-09	G
NEW XP_001946173.1	PREDICTED: similar to GA15301-PA [Acyrthosiphon pisum]	68.6	68.6	32%	1e-09	UGM
NEW XP_002057094.1	GJ16894 [Drosophila virilis] >gb|EDW62580.1| GJ16894 [Drosophila virilis]	67.0	67.0	32%	4e-09	G
NEW XP_002010644.1	GI21656 [Drosophila mojavensis] >gb|EDW06299.1| GI21656 [Drosophila mojavensis]	67.0	67.0	32%	4e-09	G
NEW XP_002092594.1	GE14278 [Drosophila yakuba] >gb|EDW92306.1| GE14278 [Drosophila yakuba]	66.7	66.7	27%	5e-09	G
NEW XP_001355344.2	GA15301 [Drosophila pseudoobscura pseudoobscura] >gb|EAL32401.2| GA15301 [Drosophila pseudoobscura pseudoobscura]	66.3	66.3	39%	7e-09	G
NEW XP_002025529.1	GL15246 [Drosophila persimilis] >gb|EDW31051.1| GL15246 [Drosophila persimilis]	66.3	66.3	39%	7e-09	G
NEW XP_001606517.1	PREDICTED: similar to CG2206-PA [Nasonia vitripennis]	65.9	65.9	30%	9e-09	UGM
NEW XP_001992028.1	GH24541 [Drosophila grimshawi] >gb|EDV91735.1| GH24541 [Drosophila grimshawi]	65.5	65.5	32%	1e-08	G
NEW ADD20695.1	hypothetical conserved secreted protein [Glossina morsitans morsitans]	65.1	65.1	27%	1e-08	
NEW EFA12874.1	hypothetical protein TcasGA2_TC010726 [Tribolium castaneum]	64.3	64.3	34%	2e-08	
NEW EFA08551.1	hypothetical protein TcasGA2_TC006206 [Tribolium castaneum]	64.0	64.0	26%	3e-08	
NEW XP_001960321.1	GF11569 [Drosophila ananassae] >gb|EDV37143.1| GF11569 [Drosophila ananassae]	63.6	63.6	27%	4e-08	G
NEW XP_001603438.1	PREDICTED: similar to GA15301-PA [Nasonia vitripennis]	63.2	63.2	40%	6e-08	UGM
NEW EFA08552.1	hypothetical protein TcasGA2_TC006207 [Tribolium castaneum]	62.4	62.4	30%	9e-08	
NEW XP_001360382.2	GA15824 [Drosophila pseudoobscura pseudoobscura] >gb|EAL24957.2| GA15824 [Drosophila pseudoobscura pseudoobscura]	62.4	62.4	27%	9e-08	G
NEW XP_002015853.1	GL10792 [Drosophila persimilis] >gb|EDW31743.1| GL10792 [Drosophila persimilis]	62.4	62.4	27%	9e-08	G
NEW XP_969565.1	PREDICTED: similar to lethal (1) G0193 CG2206-PA [Tribolium castaneum]	62.4	62.4	30%	9e-08	UGM
NEW XP_001603409.1	PREDICTED: similar to CG2206-PA [Nasonia vitripennis]	61.6	61.6	35%	2e-07	UGM
NEW XP_001602724.1	PREDICTED: similar to CG2206-PA [Nasonia vitripennis]	59.7	59.7	30%	6e-07	GM
NEW XP_002049066.1	GJ21381 [Drosophila virilis] >gb|EDW60259.1| GJ21381 [Drosophila virilis]	59.3	59.3	27%	8e-07	G
NEW XP_002062683.1	GK19560 [Drosophila willistoni] >gb|EDW73669.1| GK19560 [Drosophila willistoni]	58.2	58.2	27%	2e-06	G
NEW XP_001600223.1	PREDICTED: similar to GA15301-PA [Nasonia vitripennis]	57.4	57.4	65%	3e-06	GM
NEW ACZ28336.1	hypothetical conserved protein [Simulium nigrimanum]	57.0	57.0	33%	4e-06	
NEW XP_001606530.1	PREDICTED: similar to CG2206-PA [Nasonia vitripennis]	57.0	57.0	34%	4e-06	GM
NEW XP_001603377.1	PREDICTED: similar to CG2206-PA [Nasonia vitripennis]	57.0	57.0	26%	4e-06	GM
NEW ACH56860.1	hypothetical protein [Simulium vittatum]	56.3	56.3	33%	7e-06	
NEW CBM69271.1	venom protein Ci-48a [Chelonus inanitus]	53.6	53.6	26%	4e-05	
NEW XP_001601835.1	PREDICTED: similar to ef hand protein [Nasonia vitripennis]	52.0	52.0	29%	1e-04	UGM
NEW ADJ67267.1	Cul n 3 allergen [Culicoides nubeculosus]	50.1	50.1	33%	5e-04	
NEW ADJ67269.1	Cul n 5 allergen [Culicoides nubeculosus]	47.8	47.8	32%	0.002	
NEW ABY19389.1	venom protein 2 [Microctonus hyperodae]	47.0	47.0	27%	0.004	
NEW XP_001605439.1	PREDICTED: similar to ENSANGP00000010019 [Nasonia vitripennis]	47.0	47.0	38%	0.004	GM

Sequences with pattern at position and E-value WORSE than threshold

Accession	Description	Max score	Total score	Query coverage	E value	Links
NEW ACM40903.1	secreted salivary protein [Culicoides nubeculosus]	45.9	45.9	32%	0.009	
NEW ACE75210.1	conserved hypothetical protein [Glyptapanteles flavicoxis]	45.9	45.9	28%	0.009	
NEW XP_001605051.1	PREDICTED: similar to GA15301-PA [Nasonia vitripennis]	45.5	45.5	26%	0.012	UGM
NEW XP_002044470.1	GM11959 [Drosophila sechellia] >gb|EDW53677.1| GM11959 [Drosophila sechellia]	43.9	43.9	44%	0.035	G
NEW ACM40897.1	putative cysteine endopeptidase [Culicoides nubeculosus]	43.5	43.5	32%	0.045	
NEW ADJ67274.1	Cul n 10 allergen [Culicoides nubeculosus]	42.8	42.8	31%	0.077	
NEW XP_001601809.1	PREDICTED: similar to GA15301-PA [Nasonia vitripennis]	42.4	42.4	15%	0.10	GM
NEW ACE75299.1	conserved hypothetical protein [Glyptapanteles indiensis]	42.0	42.0	28%	0.13	
NEW ACH56924.1	hypothetical protein [Simulium vittatum]	40.5	40.5	39%	0.38	
NEW NP_001138187.1	protein tyrosine phosphatase 10D, isoform E [Drosophila melanogaster] >gb|ACL82919.1| protein tyrosine phosphatase 10D, isoform E [Drosophila melanogaster]	38.2	38.2	28%	1.9	UG
NEW XP_001355649.2	GA14821 [Drosophila pseudoobscura pseudoobscura] >gb|EAL32708.2| GA14821 [Drosophila pseudoobscura pseudoobscura]	38.2	38.2	28%	1.9	G
NEW XP_002106698.1	Ptp10D [Drosophila simulans] >gb|EDX17660.1| Ptp10D [Drosophila simulans]	38.2	38.2	28%	1.9	UG
NEW XP_002101393.1	GE15658 [Drosophila yakuba] >gb|EDX02501.1| GE15658 [Drosophila yakuba]	38.2	38.2	28%	1.9	G
NEW XP_002044105.1	GM13099 [Drosophila sechellia] >gb|EDW51417.1| GM13099 [Drosophila sechellia]	38.2	38.2	28%	1.9	G
NEW XP_002022578.1	GL13109 [Drosophila persimilis] >gb|EDW26613.1| GL13109 [Drosophila persimilis]	38.2	38.2	28%	1.9	G
NEW XP_001946656.1	PREDICTED: similar to 120.7 kDa protein in NOF-FB transposable element, partial [Acyrthosiphon pisum]	38.2	38.2	27%	1.9	GM
NEW XP_001977094.1	GG18436 [Drosophila erecta] >gb|EDV46021.1| GG18436 [Drosophila erecta]	38.2	38.2	28%	1.9	G
NEW XP_001966042.1	GF19443 [Drosophila ananassae] >gb|EDV38451.1| GF19443 [Drosophila ananassae]	38.2	38.2	28%	1.9	G
NEW AAO42638.1	RE52018p [Drosophila melanogaster]	38.2	38.2	28%	1.9	
NEW AAA28952.1	receptor-linked protein tyrosine phosphatase [Drosophila melanogaster]	38.2	38.2	28%	1.9	
NEW AAA28484.1	protein tyrosine phosphatase [Drosophila melanogaster]	38.2	38.2	28%	1.9	
NEW NP_996413.1	protein tyrosine phosphatase 10D, isoform D [Drosophila melanogaster] >ref|NP_996414.1| protein tyrosine phosphatase 10D, isoform A [Drosophila melanogaster] >gb|AAS65319.1| protein tyrosine phosphatase 10D, isoform A [Drosophila melanogaster] >gb|AAS65320.1| protein tyrosine phosphatase 10D, isoform D [Drosophila melanogaster]	38.2	38.2	28%	1.9	UG
NEW NP_727544.2	protein tyrosine phosphatase 10D, isoform B [Drosophila melanogaster] >sp|P35992.3|PTP10_DROME RecName: Full=Tyrosine-protein phosphatase 10D; AltName: Full=Receptor-linked protein-tyrosine phosphatase 10D; Short=DPTP10D; Flags: Precursor >gb|AAF48072.3| protein tyrosine phosphatase 10D, isoform B [Drosophila melanogaster]	38.2	38.2	28%	1.9	G
NEW CBM69274.1	venom protein Ci-60 [Chelonus inanitus]	37.8	37.8	16%	2.5	
NEW ACH56925.1	hypothetical protein [Simulium vittatum]	37.8	37.8	26%	2.5	
NEW ACZ28340.1	hypothetical conserved protein [Simulium nigrimanum]	37.4	37.4	26%	3.2	
NEW BAE94118.1	S-layer protein [Clostridium difficile]	37.0	37.0	43%	4.2	
NEW YP_003292364.1	putative secreted protein [Lactobacillus johnsonii FI9785] >emb|CAX66097.1| putative secreted protein [Lactobacillus johnsonii FI9785]	36.6	36.6	20%	5.5	G
NEW XP_002040062.1	GM15999 [Drosophila sechellia] >gb|EDW56927.1| GM15999 [Drosophila sechellia]	36.6	36.6	15%	5.5	G
NEW XP_315969.4	AGAP005939-PA [Anopheles gambiae str. PEST] >gb|EAA11364.4| AGAP005939-PA [Anopheles gambiae str. PEST]	36.6	36.6	32%	5.5	UG
NEW EFA79656.1	DNA-dependent protein kinase subunit [Polysphondylium pallidum PN500]	36.2	36.2	31%	7.2	
NEW ZP_04797167.1	cell wall associated fibronectin-binding protein [Staphylococcus epidermidis W23144] >gb|EES36170.1| cell wall associated fibronectin-binding protein [Staphylococcus epidermidis W23144]	36.2	36.2	52%	7.2	
NEW ZP_04006822.1	conserved hypothetical protein [Lactobacillus johnsonii ATCC 33200] >gb|EEJ60332.1| conserved hypothetical protein [Lactobacillus johnsonii ATCC 33200]	35.8	35.8	20%	9.4	

Alignments

```
>gb|ACZ28230.1|  hypothetical secreted protein [Simulium nigrimanum]
Length=362

 Score =  605 bits (1560),  Expect = 3e-171
 Identities = 228/362 (63%), Positives = 281/362 (78%), Gaps = 1/362 (0%)

Query  1    MKSELSALIIASISAVANGYLSDLTAAEKMANVINKAAKDDAKKSIRDSKFLNSAMDQLN  60
            MKS L   L   S  A+ANGYLSDL+AAE+MA  I + ++ D K  +R+SKFL+  MD+LN
Sbjct  1    MKSCLVVLTFVSALALANGYLSDLSAAERMAETIYEVSQKDRKNGVRNSKFLSPGMDKLN  60

Query  61   LEFINPYNTTNNHVGMEEGLAKKLQSEPTTCDAARTISYTIRERSDYFGNQLANNRPDDR  120
             E INPYN T+NHVG+EE LAK L+ EP TCDAART+SY+I+ERSDYF NQL  NRP D
Sbjct  61   KELINPYNVTSNHVGLEETLAKGLKQEPATCDAARTLSYSIQERSDYFNQLNGNRPSDH  120

Query  121  ILCPVTIPKMQREHELVMGFLLNRLIRIFALRAKKCTDNWESKDRLARGLNRVAENRFGND  180
            ILCP+ IP MQREHELVMGYLLNRLR+   LR+KKC D+ E+KDKVAKALKRLAENRFANE
Sbjct  121  ILCPINIPTMQREHELVMGYLLNRLRMHVLRSKKCEDSVETKEKVAKALKRLAENRFANE  180

Query  181  GEVVRSCKQNNVAVGASCMYAGDLKAGANEVITGMRLTKVGNAIWPEIQVAKVTLFGLID  240
            GE ++SCKQ+N  + ++C+  G LKAG NEVITGMRL K+ N+I  E+QVAKVT+FGLID
Sbjct  181  GEAIKSCKQSNEDIKSTCINTGVLKAGPNEVITGLRLRKITNSIILEMQVAKVTVFGLID  240

Query  241  KSTRKWGVPGGKMKSLCAPIKSLVAAEPTRLPAGHVLTQVQLVGTTKKNVKVGGQKLNFY  300
            KST+KWVGPG   K+ C PIK+L+AA+PTR RLP G+VLTQVQLVG   K  V GQKL+FY
Sbjct  241  KSTQKWVGPGSAAKT-CLPIKNLIAAKPVRLPQGNVLTQVQLVGKAKNGLVAGQKLDFY  299

Query  301  TGKLDKPKTAPAKKVVKLNDLVSDIEPVYVSTNVAGAGLRYDVETKTITPVLVAQNMAT  360
            TGKL K      K VKLN  VS++ EPVYVST VAGAGL+YD E KTI+PVL+AQNMA
Sbjct  300  TGKLTKGAKGQVKDVFKLNGAVSINTEPVYVSTTVAGAGLKYDEERKTISPVLIAQNMAV  359

Query  361  LK  362
             K
Sbjct  360  YK  361

>gb|ACZ28349.1|  hypothetical conserved protein [Simulium nigrimanum]
Length=356

 Score =  589 bits (1519),  Expect = 2e-166
 Identities = 223/356 (63%), Positives = 274/356 (77%), Gaps = 1/356 (0%)

Query  7    ALIIASISAVANGYLSDLTAAEKMANVINKAAKDDAKKSIRDSKFLNSAMDQLNLEFINP  66
             L   S  A+ANGYLSDL+AAE+MA  I + ++ D K  +R+SKFL+  MD+LN E INP
Sbjct  1    VLTFVSALALANGYLSDLSAAERMAETIYEVSQKDRKNGVRNSKFLSPGMDKLNKELINP  60

Query  67   YNTTNNHVGMEEGLAKKLQSEPTTCDAARTISYTIRERSDYFGNQLANNRPDDRILCPVT  126
            YN T NHVG+EE L K L+ EP TCDAART+SY+I+ERSDYF NQL  NRP D ILCP+
Sbjct  61   YNVTGNHVGLEETLTKGLKQEPATCDAARTLSYSIQERSDYFNQLNGNRPSDHILCPI-  120

Query  127  IPKMQREHELVMGFLLNRLIRIFALRAKKCTDNWESKDRLARGLNRVAENRFGNDGEVVRS  186
            IP MQREHELVMG+LLNRLR+  LRAKKC D+ E+K++++A+ L RAENRF NE GE ++S
Sbjct  121  IPTMQREHELVMGYLLNRLRMHVLRAKKCEDSVETKEKVAKALKRLAENRFANEGEAIKS  180

Query  187  CKQNNVAVGASCMYAGDLKAGANEVITGMRLTKVGNAIWPEIQVAKVTLFGLIDKSTRKW  246
            CKQ+N   + ++C+    LKAG NEVITG+RL K+ N+I  E+QVAKVT+FGLIDKST+KW
Sbjct  181  CKQSNEDIKSTCINTAVLKAGPNEVITGLRLRKITNSIILEMQVAKVTVFGLIDKSTQKW  240

Query  247  VGPGGKMKSLCAPIKSLVAAEPTRLPAGHVLTQVQLVGTTKKNVKVGGQKLNFYTGKLDK  306
            VGPG   K+ C PIK+L+AA+PTR RLP G+VLTQVQLVG  K  V GQKL+FYTGKL K
Sbjct  241  VGPGSAAKT-CLPIKNLIAAKPVRLPQGNVLTQVQLVGKAKNGLVAGQKLDFYTGKLTK  299

Query  307  PKTAPAKKVVKLNDLVSDIEPVYVSTNVAGAGLRYDVETKTITPVLVAQNMATLK  362
                  K VKLN  VS++ EPVYVST VAGAGL+YD E KTI+PVL+AQNMA  K
Sbjct  300  GAKGQVKDVFKLNGAVSINTEPVYVSTTVAGAGLKYDEERKTISPVLIAQNMAVYK  355

>gb|ACZ28321.1|  hypothetical protein [Simulium nigrimanum]
Length=242

 Score =  410 bits (1053),  Expect = 2e-112
 Identities = 159/243 (66%), Positives = 198/243 (82%), Gaps = 1/243 (0%)

Query  56   MDQLNLEFINPYNTTNNHVGMEEGLAKKLQSEPTTCDAARTISYTIRERSDYFGNQLANN  115
            MD+LN E INPYN T+NHVG+EE LAK L+ EP TCDAART+SY+I+ERSDYF NQL N
Sbjct  1    MDKLNKELINPYNVTSNHVGLEETLAKGLKQEPATCDAARTLSYSIQERSDYFNQLNGN  60

Query  116  RPDDRILCPVTIPKMQREHELVMGFLLNRLIRIFALRAKKCTDNWESKDRLARGLNRVAEN  175
            RP D ILCP+ IP MQREHELVMG+LLNRLR+  LR+KKC D+ E+KDKVAKALKRLAEN
Sbjct  61   RPSDHILCPINIPTMQREHELVMGYLLNRLRMHVLRSKKCEDSVETKEKVAKALKRLAEN  120

Query  176  RFGNDGEVVRSCKQNNVAVGASCMYAGDLKAGANEVITGMRLTKVGNAIWPEIQVAKVT  235
            RFGN+GE ++SCKQ+N  + ++C+  G LKAG NEVITG+RL K+ N+I  E+QVAKVT
Sbjct  121  RFGNEGEAIKSCKQSNEDIKSTCINTGVLKAGPNEVITGLRLRKITNSIILEMQVAKVT  180

Query  236  FGLIDKSTRKWGVPGGKMKSLCAPIKSLVAAEPTRLPAGHVLTQVQLVGTTKKNVKVGGQ  295
            FGLIDKST+KWVGPG   K+ C PIK+L+AA+PTR RLP G+VLTQVQLVG  K  V GQ
Sbjct  181  FGLIDKSTQKWVGPGSAAKT-CLPIKNLIAAKPVRLPQGNVLTQVQLVGKAKDGLLVAGQ  239

Query  296  KLN  298
            KL+
Sbjct  240  KLD  242

>gb|ACZ28152.1|  hypothetical conserved protein [Simulium nigrimanum]
Length=293

 Score =  130 bits (328),  Expect = 2e-28
 Identities = 32/137 (24%), Positives = 57/137 (42%), Gaps = 13/137 (9%)

Query  193  AVGASCMYAGDLKA-GANEVITGMRLTKVGNAIWPEIQVAKVTLFGLIDKSTRKWVG-PG  250
                +  +Y G  K       + V+TG+RL          ++ ++Q  K   FG ID  T +W   P
Sbjct  60   DDEENYVYLGPSKVTDPDRVVTGVRLVLENGVLYWQLQTGKPRTFGFIDSETLEWQPLPK  119

Query  251  GKMKSLCAPIKSL-----VAAEPTRLPAGHVLTQVQLVGTTKK----NVKVGGQKLNFYT  301
               K    K +       V       +P   +VLT +Q V ++          ++  + G+++
Sbjct  120  VTDKKDARLFKKINRKQSVILRNLEVPVPYVLTGIQFVKSSNPPDSFDLNLFGRQIELME  179

Query  302  GKL--DKPKTAPAKKVV  316
            G+L D   P K +
Sbjct  180  GRLFNDTNRFGPEKALF  196

>gb|ACZ28153.1|  hypothetical protein [Simulium nigrimanum]
Length=296

 Score =  129 bits (324),  Expect = 6e-28
 Identities = 33/150 (22%), Positives = 61/150 (41%), Gaps = 17/150 (11%)

Query  184  VRSCKQNN----VAVGASCMYAGDLKA-GANEVITGMRLTKVGNAIWPEIQVAKVTLFGL  238
            +R+C                  +  +Y G  K    + V+TG+RL          ++ ++Q  K   FG
Sbjct  56   LRNCDFCSCICDKDDEENYVYLGPSKVTDPDRVVAGVRLVLENGVLYWQLQTGKPRTFGF  115

Query  239  IDKSTRKWVG-PGGKMKSLCAPIKSL-----VAAEPTRLPAGHVLTQVQLVGTTKK----  288
            ID  T +W  P      K    K +       V       +P   +VLT +Q V ++
Sbjct  116  IDSETLEWQPLPKVTDKKDARLFKKINRKQSVILRNLEVPVPYVLTGIQFVKSSNPPDSF  175

Query  289  NVKVGGQKLNFYTGKL--DKPKTAPAKKVV  316
            ++  + G+++  G+L  D   P K +
Sbjct  176  DLNLFGRQIELMEGRLFNDTNRFGPEKALF  205

>gb|ACZ28354.1|  hypothetical protein [Simulium nigrimanum]
Length=291

 Score =  129 bits (323),  Expect = 8e-28
 Identities = 34/150 (23%), Positives = 61/150 (41%), Gaps = 17/150 (11%)

Query  184  VRSCKQNN----VAVGASCMYAGDLKA-GANEVITGMRLTKVGNAIWPEIQVAKVTLFGL  238
            +R+C                  +  +Y G  K    + V+TG+RL          ++ ++Q  K   FG
Sbjct  33   LRNCDFCSCICDKDDEENYVYLGPSKVTDPDRVVTGVRLVLENGVLYWQLQTGKPRTFGF  92

Query  239  IDKSTRKWVG-PGGKMKSLCAPIKSL-----VAAEPTRLPAGHVLTQVQLVGTTKK----  288
            ID  T +W  P      K    K +       V       +P   +VLT +Q V ++
Sbjct  93   IDSETLEWQPLPKVSDKKDARLFKKINRKQSVILRNLEVPVPYVLTGIQFVKSSNPPDSF  152

Query  289  NVKVGGQKLNFYTGKL--DKPKTAPAKKVV  316
            ++  + G+++  G+L  D     P K +
Sbjct  153  DLNLFGRQIELMEGRLFNDTNRFNTPFGPEKALF  182

>ref|XP_001851110.1| UG conserved hypothetical protein [Culex quinquefasciatus]
 gb|EDS33063.1| G conserved hypothetical protein [Culex quinquefasciatus]
Length=642

 GENE ID: 6041920 CpipJ_CPIJ009483 | hypothetical protein
[Culex quinquefasciatus]

                                                   Sort alignments for this subject
                                                     E value  Score  Percent identi
                                                     Query start position  Subject

 Score =  123 bits (308),  Expect = 4e-26
 Identities = 35/121 (29%), Positives = 56/121 (47%), Gaps = 16/121 (13%)

Query  203  DLKAGANEVITGMRLTKVGNAIWPEIQVAKVTLFGLIDKSTRKWVGP-----------G  250
            DLK     N+V+TGMR  K     I    +Q   ++   G ID   T +WV P
Sbjct  417  DLK--NNKVVTGMRFVKKNRIIHLIVQQGRLLPRGQIDNGTLEWVEPHNYNLSTNVRVG  474

Query  251  GKMKSLCAPIKSLVAAEPTRLPAGHVLTQVQL-VGTTKKNVKVGGQKLNFYTGKLDKPKT  309
                +L     +SL       +P  G+VLT  V+     N+++    ++++F TG+L  P
Sbjct  475  RDYHTLNRNNRSL-DLDDLYVPQGYVLTGVRFRLLDNHLNLEIRMTEIDFATGQLIDPDK  533

Query  310  A  310
             +
Sbjct  534  S  534

 Score = 42.0 bits (97),  Expect = 0.13
 Identities = 14/72 (20%), Positives = 29/72 (41%), Gaps = 2/72 (2%)

Query  188  KQNNVAVGASCMYAGDLKAGANEVITGMRLTKVGNAIWPEIQVAKVTLFG--LIDKSTRK  245
                   +         DL       V+TG+R      +   +   EI++     LID
Sbjct  476  DYHTLNRNNRSLDLDDLYVPQGYVLTGVRFRLLDNHLNLEIRMTEIDFATGQLIDPDKS  535

Query  246  WVGPGGKMKSLC  257
            W+G
Sbjct  536  WIGNDNTERTVN  547

>ref|XP_001851111.1| G conserved hypothetical protein [Culex quinquefasciatus]
 gb|EDS33064.1| G conserved hypothetical protein [Culex quinquefasciatus]
Length=721

 GENE ID: 6041924 CpipJ_CPIJ009484 | hypothetical protein
[Culex quinquefasciatus]

                                                   Sort alignments for this subject
                                                     E value  Score  Percent identi
                                                     Query start position  Subject

 Score =  113 bits (282),  Expect = 5e-23
 Identities = 31/116 (27%), Positives = 53/116 (46%), Gaps = 14/116 (12%)

Query  208  ANEVITGMRLTKVGNAIWPEIQVAKVTLFGLIDKSTRKWVGP-----------GGKMKS  255
            N+V+TGMR   K     I    +Q   ++   G ID   T +WV P                      G    +
Sbjct  484  NNKVVTGMRFIKKSQIIHLIVQQGRLLPRGQIDNGTLEWVQPHNYTVMSQNVRNGRDYHT  543

Query  256  LCAPIKSLVAAEPTRLPAGHVLTQVQL-VGTTKKNVKVGGQKLNFYTGKLDKPKTA  310
            L     +SL       +P  G+V+T V+     N+++    +  +F TG+L  P +
Sbjct  544  LNRNNRSL-DLDDLYVPQGYVVTGVRFRLLGNHLNLEIRMTEKDFATGQLIDPEKS  598

 Score = 43.2 bits (100),  Expect = 0.059
 Identities = 15/63 (24%), Positives = 26/63 (42%), Gaps = 2/63 (3%)

Query  188  KQNNVAVGASCMYAGDLKAGANEVITGMRLTKVGNAIWPEIQVAKVTLFG--LIDKSTRK  245
                        +   DL       V+TG+R    +GN + + EI++     LID
Sbjct  540  DYHTLNRNNRSLDLDDLYVPQGYVVTGVRFRLLGNHLNLEIRMTEKDFATGQLIDPEKS  599

Query  246  WVG  248
            W+G
Sbjct  600  WIG  602

>ref|XP_001851115.1| UG conserved hypothetical protein [Culex quinquefasciatus]
 gb|EDS33068.1| G conserved hypothetical protein [Culex quinquefasciatus]
Length=548

 GENE ID: 6041925 CpipJ_CPIJ009488 | hypothetical protein
[Culex quinquefasciatus]

                                                   Sort alignments for this subject
                                                     E value  Score  Percent identi
                                                     Query start position  Subject

 Score =  107 bits (268),  Expect = 2e-21
 Identities = 29/115 (25%), Positives = 53/115 (47%), Gaps = 14/115 (12%)

Query  209  NEVITGMRLTKVGNAIWPEIQVAKVTLFGLIDKSTRKWVGP-----------GGKMKSL  256
            N+V+TGMR  K     I    +Q   ++   G ID   T +WV P                      G    +L
Sbjct  420  NKVVTGMRFIKKNRIIHLIVQQGRLLPRGQIDNATLEWVQPEDYNVLSPNVRNGRDYHTL  479

Query  257  CAPIKSLVAAEPTRLPAGHVLTQVQL-VGTTKKNVKVGGQKLNFYTGKLDKPKTA  310
                 +SL       +P  G+V+T V+     N+++    +  +F TG+L  P +
Sbjct  480  NRDNR-ILDLDDLYVPQGYVVTGVRFRLLGSHLNLEIRMTEMDFATGHLMDPDKS  533

 Score = 42.4 bits (98),  Expect = 0.10
 Identities = 14/71 (20%), Positives = 28/71 (40%), Gaps = 2/71 (2%)

Query  188  KQNNVAVGASCMYAGDLKAGANEVITGMRLTKVGNAIWPEIQVAKVTLFG--LIDKSTRK  245
                        +   DL       V+TG+R    +G+ + + EI++     LID
Sbjct  475  DYHTLNRDNRILDLDDLYVPQGYVVTGVRFRLLGSHLNLEIRMTEMDFATGHLMDPDKS  534

Query  246  WVGPGGKMKSL  256
            W+G     S
Sbjct  535  WIGSDKTEHSD  545

>ref|XP_001851109.1| UG conserved hypothetical protein [Culex quinquefasciatus]
 gb|EDS33062.1| G conserved hypothetical protein [Culex quinquefasciatus]
Length=582

 GENE ID: 6041957 CpipJ_CPIJ009482 | hypothetical protein
[Culex quinquefasciatus]

                                                   Sort alignments for this subject
                                                     E value  Score  Percent identi
                                                     Query start position  Subject

 Score =  107 bits (267),  Expect = 3e-21
 Identities = 31/114 (27%), Positives = 52/114 (45%), Gaps = 14/114 (12%)

Query  209  NEVITGMRLTKVGNAIWPEIQVAKVTLFGLIDKSTRKWVGP-----------GGKMKSL  256
            N+V+TGMR  K     I    +Q   ++   G ID   T +WV P                      G     L
Sbjct  361  NKVVTGMRFIKKNQIIHLIVQQGRLLPRGQIDNGTLEWVQPEDYNVLSPNVRNGRDYHNL  420

Query  257  CAPIKSLVAAEPTRLPAGHVLTQVQL-VGTTKKNVKVGGQKLNFYTGKLDKPKT  309
                 +SL       +P  G+V+T V+     N+++    +  +F TG+L  P
Sbjct  421  NRINRSL-DLDDLYVPQGYVMTGVRFRLLGNHLNLEIRMTEMNFATGQLIDSNK  473

 Score = 40.1 bits (92),  Expect = 0.50
 Identities = 15/63 (23%), Positives = 28/63 (45%), Gaps = 2/63 (3%)

Query  188  KQNNVAVGASCMYAGDLKAGANEVITGMRLTKVGNAIWPEIQVAKVTLFG--LIDKSTRK  245
                        +   DL       V+TG+R    +GN + + EI++     LID
Sbjct  416  EYHTLNRINRSLDLDDLYVPQGYVMTGVRFRLLGNHLNLEIRMTEMNFATGQLIDSNKI  475

Query  246  WVG  248
            W+G
Sbjct  476  WIG  478

>ref|XP_001968326.1| G GG24571 [Drosophila erecta]
 gb|EDV57385.1| G GG24571 [Drosophila erecta]
Length=488

 GENE ID: 6543280 Dere\GG24571 | GG24571 gene product from transcript GG24571-RA
[Drosophila erecta] (10 or fewer PubMed links)

                                                   Sort alignments for this subject
                                                     E value  Score  Percent identi
                                                     Query start position  Subject

 Score =  101 bits (252),  Expect = 1e-19
 Identities = 26/101 (26%), Positives = 44/101 (44%), Gaps = 4/101 (3%)

Query  179  NDGEVVRSCKQNNVAVGASCMYAGDLKAGANEVITGMRLTKVGNAIWPEIQVAKVTLFG-  237
            +D +V         + +     GDLKA  + V+TG+R    VG   +   E Q + +
Sbjct  305  SDKDVTEHADYHTLSYEKRALDLGDLKADNSAVVTGLRFRVVGAHLQLEAQFSALEFESG  364

Query  238  -LID-KSTRKWVGPGGKMKSLCAPIKSLVAAEPTRLPAGH  275
             LID K T +W    G         P+K   V+++++    L
Sbjct  365  KLIDPKRTSQWTSLGASQHREKLPLKDAQVSSKSPELSIPY  405

 Score = 66.3 bits (160),  Expect = 7e-09
 Identities = 23/115 (20%), Positives = 44/115 (39%), Gaps = 14/115 (12%)

Query  208  ANEVITGMRLTKVGNAIWPEIQVAKVTLFGLIDKSTRKWVGP-----------GGKMKS  255
             N V+TG+R  K         ++Q    G+I++++T +W
Sbjct  258  NNRVVTGLRFVKSRRIFHLQVQEGGLLPLGVINKPTLQWKPLDAYSISDKDVTEHADYHT  317

Query  256  LCAPIKSLVAAEPTRLPAGHVLTQVQL-VGTTKKNVKVGGQKLNFYTGKLDKPKT  309
            L      ++L              V+T ++      V           L   F +GKL  P
Sbjct  318  LSYEKRAL-DLGDLKADNSAVVTGLRFRVVGAHLQLEAQFSALEFESGKLIDPKR  371

>ref|XP_001648416.1| UG hypothetical protein AaeL_AAEL014309 [Aedes aegypti]
 gb|EAT33416.1| G conserved hypothetical protein [Aedes aegypti]
Length=663

 GENE ID: 5564091 AaeL_AAEL014309 | hypothetical protein [Aedes aegypti]
(10 or fewer PubMed links)

                                                   Sort alignments for this subject
                                                     E value  Score  Percent identi
                                                     Query start position  Subject

 Score = 90.2 bits (222),  Expect = 4e-16
 Identities = 27/116 (24%), Positives = 52/116 (45%), Gaps = 14/116 (12%)

Query  208  ANEVITGMRLTKVGNAIWPEIQVAKVTLFGLIDKSTRKWVGP-----------GGKMKS  255
             N+V+TG+R  K         I+Q    G+ID   T +WV P
Sbjct  426  NNKVVTGLRFIKKNRIIFLIVQEGQLLPRGQIDNTTLQWVEPEAYNNLSPIYRAKVDYNN  485

Query  256  LCAPIKSLVAAEPTRLPAGHVLTQVQL-VGTTKKNVKVGGQKLNFYTGKLDKPKTA  310
                  +++     L      +V+T V+    T   N++V    +++F TG+L  P  +
Sbjct  486  MNYENRAM-DLDDIILQPPYVVTGVRFRMLGTHMNLEVRMTEMDFGSGKLIDPDKS  540

 Score = 50.5 bits (119),  Expect = 4e-04
 Identities = 20/113 (18%), Positives = 45/113 (40%), Gaps = 7/113 (6%)

Query  188  KQNNVAVGASCMYAGDLKAGANEVITGMRLTKVGNAIWPEIQVAKVTLFG--LIDKSTRK  245
            N    +     M   D+       V+TG+R    +G  + + E+++     LID
Sbjct  482  DYNNMNYENRAMDLDDIILQPPYVVTGVRFRMLGTHMNLEVRMTEMDFGSGKLIDPDKS  541

Query  246  WVGPGGKMKSLCAPIKSLVAAEPTRLPAGHVLTQVQLVGTTKKNVKVGGQKLN  298
            WVG        S    ++ ++  +P     +L+   V    ++  N    ++ ++
Sbjct  542  WVGSDKTEHSEDK--RTEMKLDKPHIP---ILSPTKSVPDSEPNQFIKFRESD  589

>ref|XP_001648417.1| UG hypothetical protein AaeL_AAEL014309 [Aedes aegypti]
 gb|EAT33417.1| G conserved hypothetical protein [Aedes aegypti]
Length=684

 GENE ID: 5564091 AaeL_AAEL014309 | hypothetical protein [Aedes aegypti]
(10 or fewer PubMed links)

                                                   Sort alignments for this subject
                                                     E value  Score  Percent identi
                                                     Query start position  Subject

 Score = 90.2 bits (222),  Expect = 4e-16
 Identities = 27/116 (24%), Positives = 52/116 (45%), Gaps = 14/116 (12%)

Query  208  ANEVITGMRLTKVGNAIWPEIQVAKVTLFGLIDKSTRKWVGP-----------GGKMKS  255
             N+V+TG+R  K         I+Q    G+ID   T +WV P
Sbjct  426  NNKVVTGLRFIKKNRIIFLIVQEGQLLPRGQIDNTTLQWVEPEAYNNLSPIYRAKVDYNN  485

Query  256  LCAPIKSLVAAEPTRLPAGHVLTQVQL-VGTTKKNVKVGGQKLNFYTGKLDKPKTA  310
                  +++     L      +V+T V+    T   N++V    +++F TG+L  P  +
Sbjct  486  MNYENRAM-DLDDIILQPPYVVTGVRFRMLGTHMNLEVRMTEMDFGSGKLIDPDKS  540

 Score = 47.0 bits (110),  Expect = 0.004
 Identities = 16/71 (23%), Positives = 27/71 (39%), Gaps = 2/71 (2%)

Query  188  KQNNVAVGASCMYAGDLKAGANEVITGMRLTKVGNAIWPEIQVAKVTLFG--LIDKSTRK  245
            N    +     M   D+       V+TG+R    +G  + + E+++     LID
Sbjct  482  DYNNMNYENRAMDLDDIILQPPYVVTGVRFRMLGTHMNLEVRMTEMDFGSGKLIDPDKS  541

Query  246  WVGPGGKMKSL  256
            WVG     S
Sbjct  542  WVGSDKTEHSE  552

>gb|EFR25576.1|  hypothetical protein AND_08977 [Anopheles darlingi]
Length=686

                                                   Sort alignments for this subject
                                                     E value  Score  Percent identi
                                                     Query start position  Subject

 Score = 89.8 bits (221),  Expect = 6e-16
 Identities = 29/115 (26%), Positives = 51/115 (45%), Gaps = 14/115 (12%)

Query  209  NEVITGMRLTKVGNAIWPEIQVAKVTLFGLIDKSTRKWVGP-----------GGKMKSL  256
            N+V+TG+R  K         I+Q    G+ID   T +WV P                      G    +L
Sbjct  437  NKVVTGLRFLKQDRVIHLAVQQGTLMPGGEIDNRTLEWVPPRYFSVLSKGIRDGLDYHTL  496

Query  257  CAPIKSLVAAEPTRLPAGHVLTQVQL-VGTTKKNVKVGGQKLNFYTGKLDKPKTA  310
                 +S+    ++  G+VLT V+     T  N+    ++NF+TG+L  P
Sbjct  497  TYDNRSM-DLDDVQVPYGYVLTGVRFRLLGTHLNLRMIRMTEMNFTSGQLMDLDKS  550

 Score = 43.2 bits (100),  Expect = 0.059
 Identities = 31/182 (18%), Positives = 63/182 (35%), Gaps = 33/182 (18%)

Query  188  KQNNVAVGASCMYAGDLKAGANEVITGMRLTKVGNAIWPEIQVAKVTLFG--LIDKSTRK  245
            + +           M  D++       V+TG+R    +G  + +   I++++     L+D
Sbjct  492  DYHTLTYDNRSMDLDDVQVPYGYVLTGVRFRLLGTHLNLRMIRMTEMNFTSGQLMDLDKS  551

Query  246  WVGPGGKMKSLCAPIKSLVAAEPTRLPAGHVLTQV--QLVGTTKKNVKVGGQKLNFYTGK  303
            W+G           + +A     L   V    T  +  +  ++   +  +   V    +
Sbjct  552  WIG-----------NDNTIARTELVLQPDVVPTKALSKSVPDSTNGQYVRFTPTD-----  595

Query  304  LDKPKTAPAKKVVKLNDLVSDIEPVYVSTNV--AGAGLRYDVETKTITPVLVAQNM  358
                   + A    +D +PVY     V   +GAG++  +K+  P+    L+ Q +
Sbjct  596  -READAAQTTVPF-------LDAQPVYTHRPVPLSGAGIFHKGQKSKGGFLAPKLLTYDF  647

Query  359  AT  360
             T
Sbjct  648  TT  649

>gb|ACH56909.1|  hypothetical protein [Simulium vittatum]
Length=313

 Score = 89.0 bits (219),  Expect = 9e-16
 Identities = 32/132 (25%), Positives = 54/132 (41%), Gaps = 14/132 (10%)

Query  199  MYAGDLKAGA-NEVITGMRLTKVGNAIWPEIQVAKVTLFGLIDKSTRKWVG-PGGKMKSL  256
            +Y G ++       V+TG+RL          N ++ ++Q  K   FG ID  T  +W    P       K
Sbjct  78   VYLGAIQVANLGHVVTGVRLVMANNVLFWQLQTGKPRSFGFIDADTLAWQPLPKIDRKKE  137

Query  257  CAPI-----KSLVAAEPTRLPAGHVLTQVQLVGTTKK-----NVKVGGQKLNFYTGKL--  304
                  +       +PA +VLT VQ       +           +       +    +   +++  GKL
Sbjct  138  AKLLHRVNKQSFIMRNLEVPAPYVVTGVQFFARSLNQNAAYDMNLYGRPIDLMEGKLYN  197

Query  305  DKPKTAPAKKVV  316
                +  P K+
Sbjct  198  ESRQFTPNKEQF  209

>ref|XP_002087581.1| G GE15356 [Drosophila yakuba]
 gb|EDW87293.1| G GE15356 [Drosophila yakuba]
Length=489

 GENE ID: 6526462 Dyak\GE15356 | GE15356 gene product from transcript GE15356-RA
[Drosophila yakuba] (10 or fewer PubMed links)

                                                   Sort alignments for this subject
                                                     E value  Score  Percent identi
                                                     Query start position  Subject

 Score = 81.7 bits (200),  Expect = 1e-13
 Identities = 23/97 (24%), Positives = 42/97 (44%), Gaps = 4/97 (4%)

Query  180  DGEVVRSCKQNNVAVGASCMYAGDLKAGANEVITGMRLTKVGNAIWPEIQVAKVTLFG-  237
            D  V          +  +  +   GD+KA   N  V+TG+R    +G   +   E Q + +
Sbjct  306  DKNVTDTVDYHKLSYESRSLDLGDVKAENNSAVVTGLRFRVMGGHLQLQVQFSAVEFESG  365

Query  238  -LID-KSTRKWVGPGGKMKSLCAPIKSLVAAEPTRLP  272
             LID  T   W   G         P++  +  +P
Sbjct  366  KLIDPTKTSHWTSLGTDQKREKLPLRDAQVSSKSSVP  402

 Score = 65.1 bits (157),  Expect = 1e-08
 Identities = 24/115 (21%), Positives = 42/115 (37%), Gaps = 13/115 (11%)

Query  208  ANEVITGMRLTKVGNAIWPEIQVAKVTLFGLIDKSTRKWVGP-----------GGKMKS  255
             N V+TG+R  K         ++Q    G+I+++T +W
Sbjct  258  NNRVVTGLRFVKKSRRIFHLQVQEGELLPLGVINKPTLQWKPVDSYSIFDKNVTDTVDYH  317

Query  256  LCAPIKSLVAAEPTRLPAGHVLTQVQL-VGTTKKNVKVGGQKLNFYTGKLDKPKT  309
            L     +SL              V+T ++      ++           +   F +GKL  P
Sbjct  318  LSYESRSL-DLGDVKAENNSAVVTGLRFRVMGGHLQLQVQFSAVEFESGKLIDPTK  372

>gb|EFA08554.1|  hypothetical protein TcasGA2_TC006209 [Tribolium castaneum]
Length=640

                                                   Sort alignments for this subject
                                                     E value  Score  Percent identi
                                                     Query start position  Subject

 Score = 80.9 bits (198),  Expect = 3e-13
 Identities = 34/125 (28%), Positives = 51/125 (41%), Gaps = 17/125 (13%)

Query  208  ANEVITGMRLTKVGNAIWPEIQVAKVTLFGLIDKSTRKWVGP-----------GGKMKS  255
             N V+TG+R  K     I  +IQ  K+  G ID  T  +WV P
Sbjct  407  NNRVVTGLRFVKHNRIIHLQIQEGKLLPRGNIDVTTVHWVPVEEYKITDSNVANAQDYHT  466

Query  256  LCAPIKSLVAAEPTRLPAGHVLTQVQL-VGTTKKNVKVGGQKLNFYTGKLDKPKTAPAKK  314
            L     K    V        +        G+V+T V+      ++++     +L+   TG+L  P+T      K
Sbjct  467  LTWE-KRAVDLDDLVADEGYVVTGVRFKVIGSHLNFEIYTTPFDFETGQLIDPET---KS  522

Query  315  VVKLN  319
              + K N
Sbjct  523  MYKDN  527

 Score = 47.0 bits (110),  Expect = 0.004
 Identities = 16/67 (24%), Positives = 25/67 (38%), Gaps = 2/67 (2%)

Query  179  NDGEVVRSCKQNNVAVGASCMYAGDLKAGANEVITGMRLTKVGNAIWPEIQVAKVTLFG-  237
              D   V          +       DL A     V+TG+R    +G+ +    EI
Sbjct  454  TDSNVANAQDYHTLTWEKRAVDLDDLVADEGYVVTGVRFKVIGSHLNFEIYTTPFDFETG  513

Query  238  -LIDKST  243
             LID   T
Sbjct  514  QLIDPET  520

>ref|XP_969643.2| UGM PREDICTED: similar to GA15301-PA [Tribolium castaneum]
Length=660

 GENE ID: 658141 LOC658141 | similar to CG2206-PB, isoform B
[Tribolium castaneum]

                                                   Sort alignments for this subject
                                                     E value  Score  Percent identi
                                                     Query start position  Subject

 Score = 80.9 bits (198),  Expect = 3e-13
 Identities = 34/125 (28%), Positives = 51/125 (41%), Gaps = 17/125 (13%)

Query  208  ANEVITGMRLTKVGNAIWPEIQVAKVTLFGLIDKSTRKWVGP-----------GGKMKS  255
             N V+TG+R  K     I  +IQ  K+  G ID  T  +WV P
Sbjct  427  NNRVVTGLRFVKHNRIIHLQIQEGKLLPRGNIDVTTVHWVPVEEYKITDSNVANAQDYHT  486

Query  256  LCAPIKSLVAAEPTRLPAGHVLTQVQL-VGTTKKNVKVGGQKLNFYTGKLDKPKTAPAKK  314
            L     K    V        +        G+V+T V+      ++++     +L+   TG+L  P+T      K
Sbjct  487  LTWE-KRAVDLDDLVADEGYVVTGVRFKVIGSHLNFEIYTTPFDFETGQLIDPET---KS  542

Query  315  VVKLN  319
              + K N
Sbjct  543  MYKDN  547

 Score = 47.0 bits (110),  Expect = 0.004
 Identities = 16/67 (24%), Positives = 25/67 (38%), Gaps = 2/67 (2%)

Query  179  NDGEVVRSCKQNNVAVGASCMYAGDLKAGANEVITGMRLTKVGNAIWPEIQVAKVTLFG-  237
              D   V          +       DL A     V+TG+R    +G+ +    EI
Sbjct  474  TDSNVANAQDYHTLTWEKRAVDLDDLVADEGYVVTGVRFKVIGSHLNFEIYTTPFDFETG  533

Query  238  -LIDKST  243
             LID   T
Sbjct  534  QLIDPET  540

>ref|XP_310721.4| UG AGAP000385-PA [Anopheles gambiae str. PEST]
 gb|EAA06714.4| G AGAP000385-PA [Anopheles gambiae str. PEST]
Length=667

 GENE ID: 1271866 AgaP_AGAP000385 | AGAP000385-PA [Anopheles gambiae str. PEST]
(10 or fewer PubMed links)

                                                   Sort alignments for this subject
                                                     E value  Score  Percent identi
                                                     Query start position  Subject

 Score = 77.8 bits (190),  Expect = 2e-12
 Identities = 28/107 (27%), Positives = 46/107 (43%), Gaps = 14/107 (13%)

Query  211  VITGMRLTKVGNAIWPEIQVAKVTLFGLIDKSTRKWVGP-----------GGKMKSLCA  258
            V+TG+R  K     +Q    +  G  I  ST  +WV P                      G     +L
Sbjct  409  VVTGLRFVKRRQMVHLIVQQGLLLPGGEIANSTLEWVVPKPFTHTDKGVRDGRDYHTLSY  468

Query  259  PIKSLVAAEPTRLPAGHVLTQVQL-VGTTKKNVKVGGQKLNFYTGKL  304
               +   +   +P  G+VVT  V+     +  N+  ++  +NF  GKL
Sbjct  469  D-RRAIDLDDVHVPPGYVVTGVQFRVLGARLNLLLRMTEMNFTEGKL  514

 Score = 39.7 bits (91),  Expect = 0.65
 Identities = 7/57 (13%), Positives = 22/57 (39%), Gaps = 0/57 (0%)

Query  179  NDGEVVRSCKQNNVAVGASCMYAGDLKAGANEVITGMRLTKVGNAIWPEIQVAKVTL  235
               D    V          ++          D+       V+TG+++   +G    + +  + +++
Sbjct  453  TDKGVRDGRDYHTLSYDRRAIDLDDVHVPPGYVVTGVQFRVLGARLNLLLRMTEMNF  509

>ref|NP_722735.2|  CG31664 [Drosophila melanogaster]
 gb|AAF51336.3|  CG31664 [Drosophila melanogaster]
Length=528

                                                   Sort alignments for this subject
                                                     E value  Score  Percent identi
                                                     Query start position  Subject

 Score = 77.4 bits (189),  Expect = 3e-12
 Identities = 23/87 (27%), Positives = 34/87 (40%), Gaps = 3/87 (3%)

Query  179  NDGEVVRSCKQNNVAVGASCMYAGDLKAGANEVITGMRLTKVGNAIWPEIQVAKVTLFG-  237
            +D +V         +       +   GDL A   N   V+TG+R    G   +   E Q
Sbjct  353  SDKDVSENVDYHTLTYEKRSIDLGDLNAKINSVVTGLRFRVFGAHLQLEAQFTAVEFESG  412

Query  238  -LID-KSTRKWVGPGGKMKSLCAPIKS  262
             LID K T  W+   G             P+
Sbjct  413  KLIDPKKTSHWISMGTDQPRERLPLMD  439

 Score = 64.0 bits (154),  Expect = 3e-08
 Identities = 22/115 (19%), Positives = 41/115 (35%), Gaps = 14/115 (12%)

Query  208  ANEVITGMRLTKVGNAIWPEIQVAKVTLFGLIDKSTRKW-----------VGPGGKMKS  255
             N V+ G+R  K         ++Q    G+I+ ++T +W                              V
Sbjct  306  NNRVVVGLRFVKRNRIFHLQVQEGELLPLGDINGTTLRWKPVDSYSISDKDVSENVDYH  365

Query  256  LCAPIKSLVAAEPTRLPAGHVLTQVQL-VGTTKKNVKVGGQKLNFYTGKLDKPKT  309
            L      +S        V+T ++      ++           +   F +GKL  PK
Sbjct  366  LTYEKRSI-DLGDLNAKINSVVTGLRFRVFGAHLQLEAQFTAVEFESGKLIDPKK  419

>gb|ACH95281.1|  FI07603p [Drosophila melanogaster]
Length=571

                                                   Sort alignments for this subject
                                                     E value  Score  Percent identi
                                                     Query start position  Subject

 Score = 77.4 bits (189),  Expect = 3e-12
 Identities = 23/87 (27%), Positives = 34/87 (40%), Gaps = 3/87 (3%)

Query  179  NDGEVVRSCKQNNVAVGASCMYAGDLKAGANEVITGMRLTKVGNAIWPEIQVAKVTLFG-  237
            +D +V         +       +   GDL A   N   V+TG+R    G   +   E Q
Sbjct  396  SDKDVSENVDYHTLTYEKRSIDLGDLNAKINSVVTGLRFRVFGAHLQLEAQFTAVEFESG  455

Query  238  -LID-KSTRKWVGPGGKMKSLCAPIKS  262
             LID K T  W+   G             P+
Sbjct  456  KLIDPKKTSHWISMGTDQPRERLPLMD  482

 Score = 64.0 bits (154),  Expect = 3e-08
 Identities = 22/115 (19%), Positives = 41/115 (35%), Gaps = 14/115 (12%)

Query  208  ANEVITGMRLTKVGNAIWPEIQVAKVTLFGLIDKSTRKW-----------VGPGGKMKS  255
             N V+ G+R  K         ++Q    G+I+ ++T +W                              V
Sbjct  349  NNRVVVGLRFVKRNRIFHLQVQEGELLPLGDINGTTLRWKPVDSYSISDKDVSENVDYH  408

Query  256  LCAPIKSLVAAEPTRLPAGHVLTQVQL-VGTTKKNVKVGGQKLNFYTGKLDKPKT  309
            L      +S        V+T ++      ++           +   F +GKL  PK
Sbjct  409  LTYEKRSI-DLGDLNAKINSVVTGLRFRVFGAHLQLEAQFTAVEFESGKLIDPKK  462

>gb|AAY55318.1|  IP12601p [Drosophila melanogaster]
Length=571

                                                   Sort alignments for this subject
                                                     E value  Score  Percent identi
                                                     Query start position  Subject

 Score = 77.4 bits (189),  Expect = 3e-12
 Identities = 23/87 (27%), Positives = 34/87 (40%), Gaps = 3/87 (3%)

Query  179  NDGEVVRSCKQNNVAVGASCMYAGDLKAGANEVITGMRLTKVGNAIWPEIQVAKVTLFG-  237
            +D +V         +       +   GDL A   N   V+TG+R    G   +   E Q
Sbjct  396  SDKDVSENVDYHTLTYEKRSIDLGDLNAKINSVVTGLRFRVFGAHLQLEAQFTAVEFESG  455

Query  238  -LID-KSTRKWVGPGGKMKSLCAPIKS  262
             LID K T  W+   G             P+
Sbjct  456  KLIDPKKTSHWISMGTDQPRERLPLMD  482

 Score = 64.0 bits (154),  Expect = 3e-08
 Identities = 22/115 (19%), Positives = 41/115 (35%), Gaps = 14/115 (12%)

Query  208  ANEVITGMRLTKVGNAIWPEIQVAKVTLFGLIDKSTRKW-----------VGPGGKMKS  255
             N V+ G+R  K         ++Q    G+I+ ++T +W                              V
Sbjct  349  NNRVVVGLRFVKRNRIFHLQVQEGELLPLGDINGTTLRWKPVDSYSISDKDVSENVDYH  408

Query  256  LCAPIKSLVAAEPTRLPAGHVLTQVQL-VGTTKKNVKVGGQKLNFYTGKLDKPKT  309
            L      +S        V+T ++      ++           +   F +GKL  PK
Sbjct  409  LTYEKRSI-DLGDLNAKINSVVTGLRFRVFGAHLQLEAQFTAVEFESGKLIDPKK  462

>gb|AAY55307.1|  IP12501p [Drosophila melanogaster]
Length=579

                                                   Sort alignments for this subject
                                                     E value  Score  Percent identi
                                                     Query start position  Subject

 Score = 77.4 bits (189),  Expect = 3e-12
 Identities = 23/87 (27%), Positives = 34/87 (40%), Gaps = 3/87 (3%)

Query  179  NDGEVVRSCKQNNVAVGASCMYAGDLKAGANEVITGMRLTKVGNAIWPEIQVAKVTLFG-  237
            +D +V         +       +   GDL A   N   V+TG+R    G   +   E Q
Sbjct  404  SDKDVSENVDYHTLTYEKRSIDLGDLNAKINSVVTGLRFRVFGAHLQLEAQFTAVEFESG  463

Query  238  -LID-KSTRKWVGPGGKMKSLCAPIKS  262
             LID K T  W+   G             P+
Sbjct  464  KLIDPKKTSHWISMGTDQPRERLPLMD  490

 Score = 64.0 bits (154),  Expect = 3e-08
 Identities = 22/115 (19%), Positives = 41/115 (35%), Gaps = 14/115 (12%)

Query  208  ANEVITGMRLTKVGNAIWPEIQVAKVTLFGLIDKSTRKW-----------VGPGGKMKS  255
             N V+ G+R  K         ++Q    G+I+ ++T +W                              V
Sbjct  357  NNRVVVGLRFVKRNRIFHLQVQEGELLPLGDINGTTLRWKPVDSYSISDKDVSENVDYH  416

Query  256  LCAPIKSLVAAEPTRLPAGHVLTQVQL-VGTTKKNVKVGGQKLNFYTGKLDKPKT  309
            L      +S        V+T ++      ++           +   F +GKL  PK
Sbjct  417  LTYEKRSI-DLGDLNAKINSVVTGLRFRVFGAHLQLEAQFTAVEFESGKLIDPKK  470

>ref|XP_002077791.1| UG GD22890 [Drosophila simulans]
 gb|EDX03376.1| G GD22890 [Drosophila simulans]
Length=490

 GENE ID: 6730612 Dsim\GD22890 | GD22890 gene product from transcript GD22890-RA
[Drosophila simulans] (10 or fewer PubMed links)

                                                   Sort alignments for this subject
                                                     E value  Score  Percent identi
                                                     Query start position  Subject

 Score = 76.3 bits (186),  Expect = 6e-12
 Identities = 22/87 (26%), Positives = 34/87 (40%), Gaps = 3/87 (3%)

Query  179  NDGEVVRSCKQNNVAVGASCMYAGDLKAGANEVITGMRLTKVGNAIWPEIQVAKVTLFG-  237
            +D +V         +       +   GDL A   N   V+TG+R    G   +   E Q
Sbjct  307  SDKDVSENVDYHTLTYQKRSIDLGDLNAKINSVVTGLRFRVFGAHLQLEAQFTAVEFESG  366

Query  238  -LID-KSTRKWVGPGGKMKSLCAPIKS  262
             L+D K T  W+   G             P+
Sbjct  367  KLVDPKKTSHWISMGTDQPREKLPLMD  393

 Score = 63.6 bits (153),  Expect = 4e-08
 Identities = 23/116 (20%), Positives = 42/116 (37%), Gaps = 14/116 (12%)

Query  207  GANEVITGMRLTKVGNAIWPEIQVAKVTLFGLIDKSTRKW-----------VGPGGKMK  254
             N+V+ G+R  K         ++Q    G+I+ ++T +W                              V
Sbjct  259  DNNRVVVGLRFVKRNRIFHLQIQEGELLPLGAINETTLLWKPVDSYSISDKDVSENVDYH  318

Query  255  SLCAPIKSLVAAEPTRLPAGHVLTQVQL-VGTTKKNVKVGGQKLNFYTGKLDKPKT  309
            +L      +S        V+T ++      ++           +   F +GKL  PK
Sbjct  319  TLTYQKRSI-DLGDLNAKINSVVTGLRFRVFGAHLQLEAQFTAVEFESGKLVDPKK  373

>ref|XP_002077790.1| UG GD22891 [Drosophila simulans]
 gb|EDX03375.1| G GD22891 [Drosophila simulans]
Length=490

 GENE ID: 6730611 Dsim\GD22891 | GD22891 gene product from transcript GD22891-RA
[Drosophila simulans] (10 or fewer PubMed links)

                                                   Sort alignments for this subject
                                                     E value  Score  Percent identi
                                                     Query start position  Subject

 Score = 76.3 bits (186),  Expect = 6e-12
 Identities = 22/87 (26%), Positives = 34/87 (40%), Gaps = 3/87 (3%)

Query  179  NDGEVVRSCKQNNVAVGASCMYAGDLKAGANEVITGMRLTKVGNAIWPEIQVAKVTLFG-  237
            +D +V         +       +   GDL A   N   V+TG+R    G   +   E Q
Sbjct  307  SDKDVSENVDYHTLTYQKRSIDLGDLNAKINSVVTGLRFRVFGAHLQLEAQFTAVEFESG  366

Query  238  -LID-KSTRKWVGPGGKMKSLCAPIKS  262
             L+D K T  W+   G             P+
Sbjct  367  KLVDPKKTSHWISMGTDQPREKLPLMD  393

 Score = 64.0 bits (154),  Expect = 3e-08
 Identities = 23/116 (20%), Positives = 41/116 (35%), Gaps = 14/116 (12%)

Query  207  GANEVITGMRLTKVGNAIWPEIQVAKVTLFGLIDKSTRKW-----------VGPGGKMK  254
             N+V+ G+R  K         ++Q    G+I+ ++T +W                              V
Sbjct  259  DNNRVVVGLRFVKRNRIFHLQIQEGELLPLGAINETTLLWKPVDSYSISDKDVSENVDYH  318

Query  255  SLCAPIKSLVAAEPTRLPAGHVLTQVQL-VGTTKKNVKVGGQKLNFYTGKLDKPKT  309
            +L      +S        V+T ++      ++           +   F +GKL  PK
Sbjct  319  TLTYQKRSI-DLGDLNAKINSVVTGLRFRVFGAHLQLEAQFTAVEFESGKLVDPKK  373

>ref|XP_001121007.1| UGM PREDICTED: similar to lethal (1) G0193 CG2206-PB, isoform B [Apis
mellifera]
Length=827

 GENE ID: 725122 LOC725122 | similar to lethal (1) G0193 CG2206-PB, isoform B
[Apis mellifera] (10 or fewer PubMed links)

                                                   Sort alignments for this subject
                                                     E value  Score  Percent identi
                                                     Query start position  Subject

 Score = 74.4 bits (181),  Expect = 2e-11
 Identities = 33/135 (25%), Positives = 55/135 (41%), Gaps = 15/135 (11%)

Query  187  CKQNNVAVGAS-CMYAGDLKAGANEVITGMRLTKVGNAIWPEIQVAKVTLFGLIDKSTR  245
            C  NN+           N+V+TG+R  KV   I   +IQ  ++   G IDK+
Sbjct  569  CDDNNMNSDRYFSLRKVVSDVANNKVVTGVRFKKVNQIIHIQIQQGELMPRGNIDKASVH  628

Query  246  WVGP-----------GGKMKSLCAPIKSLVAAEPTRLPAGHVLTQVQL-VGTTKKNVKV  292
            W                  G+   +L      +L  +   LT  + V    +  N+++
Sbjct  629  WKSIEEFNVLDSNINKNGIDYHTLSWEKRGL-DLDDLTLDKNYLLTGFKFRMVGSRLNLEV  687

Query  293  GGQKLNFYTGKLDKP  307
               NF TGKL  +P
Sbjct  688  RMTPFNFTTGKLIEP  702

 Score = 40.5 bits (93),  Expect = 0.38
 Identities = 18/100 (18%), Positives = 36/100 (36%), Gaps = 14/100 (14%)

Query  180  DGEVVRSCKQNNVAVGASCMYAGDLKAGANEVITGMRLTKVGNAIWPEIQVAKVTLFGL  239
            D   +            +   +    DL       N ++TG+   K  +VG    + +E++      +T F
Sbjct  639  DSNINKNGIDYHTLSWEKRGLDLDDLTLDKNYLLTGFKFRMVGSRLNLEV---RMTPFNF  695

Query  240  DKSTRK------WVGPGGKMKSLCAPIKSLVAAEPTRLP  272
                   W       ++L           PTRL
Sbjct  696  TGKLIEPLNNSFWSHDRTDRTELKLENPNI----PTRLP  731

>ref|XP_002067370.1| G GK16218 [Drosophila willistoni]
 gb|EDW78356.1| G GK16218 [Drosophila willistoni]
Length=711

 GENE ID: 6644823 Dwil\GK16218 | GK16218 gene product from transcript GK16218-RA
[Drosophila willistoni] (10 or fewer PubMed links)

                                                   Sort alignments for this subject
                                                     E value  Score  Percent identi
                                                     Query start position  Subject

 Score = 73.6 bits (179),  Expect = 4e-11
 Identities = 28/113 (25%), Positives = 51/113 (46%), Gaps = 15/113 (13%)

Query  209  NEVITGMRLTKVGNAIWPEIQVAKVTLFGLIDKSTRKW-----------VGPGGKMKSL  256
            N V+TG+R  K+     +  A++    G ++++ST +W                              V   +     L
Sbjct  482  NRVVTGLRFVKLNRIFHLQIQEAELLPRGAVNESTLEWKRDISTTDNSYNFIDRDVKKGVDYHT  541

Query  257  CAPIKSLVAAEPTRL-PAGHVLTQVQL-VGTTKKNVKVGGQKLNFYTGKLDKP  307
            +      ++   +    +       +V+T V+    +++  +  G+L +P
Sbjct  542  SYESRS-LDLDIYTDDNSYVVTGLRFVKLNNHLNLEARLSELDFRTGQLIQP  593

 Score = 52.0 bits (123),  Expect = 1e-04
 Identities = 27/166 (17%), Positives = 57/166 (35%), Gaps = 17/166 (10%)

Query  180  DGEVVRSCKQNNVAVGASCMYAGDLKAGANE-VITGMRLTKVGNAIWPEIQVAKVTLFG-  237
            D +V +            +       +  DL     N + V+TG+R    VG    + E +++++
Sbjct  529  DRDVKKGVDYHTLSYESRSLDLDIYTDDNSYVVTGLRFVKLNNHLNLEARLSELDFRTG  588

Query  238  -LIDKSTRKWVGPGGKMKSLCAPIKSLVAAEPTRLPAGHVLTQVQLVGTTKKNVKVGGQK  296
             LI        +        +                LP    T+  + I   +         Q
Sbjct  589  QLIQPEVNSFWKSNDNTDVSGDDRRKKIPLSNADLPTRTITTSIPISRHN--------QY  640

Query  297  LNFYTGKLDKPKTAPAKKVVKLNDLVSDIEPVYVSTNVAGAGLRY  342
             +F   LDK          +   +  D+    P           +G  G+ Y
Sbjct  641  IDFTYTGLDKDAAQSTVPFIDIQDVTSKPPVP------LSGIGIY  680

>ref|XP_002076900.1| UG GD24578 [Drosophila simulans]
 gb|EDX16494.1| G GD24578 [Drosophila simulans]
Length=646

 GENE ID: 6740015 Dsim\GD24578 | GD24578 gene product from transcript GD24578-RA
[Drosophila simulans] (10 or fewer PubMed links)

                                                   Sort alignments for this subject
                                                     E value  Score  Percent identi
                                                     Query start position  Subject

 Score = 71.7 bits (174),  Expect = 2e-10
 Identities = 39/165 (24%), Positives = 64/165 (39%), Gaps = 35/165 (21%)

Query  209  NEVITGMRLTKVGNAIWPEIQVAKVTLFGLIDKSTRKWVGP-----------GGKMKSL  256
            N ++TG+R  K          +Q    G +++++T +W                              G    L
Sbjct  419  NRIVTGLRFVKQNRIFHLQIQEGELLPRGTVNQSTLEWKLPVEKYNVKPENFDRHVKNGVDYHTL  478

Query  257  CAPIKSLVAAEPTRLP-AGHVLTQVQL-VGTTKKNVKVGGQKLNFYTGKLDKPKTAPAKK  314
            ++    +   +     V     T   N++   +  TG+L  PE+
Sbjct  479  SYE-KRTIDLDDVTDENSFVVTGVRFRVVGTHLNLEAYYSEFDFRTGQLIQPEY---NS  534

Query  315  VVKLNDLVSDIEPVYVSTNVAGAGL------RYDVETKTITPVL  353
            K ND              +T+VAGA           DV  T+TI P L
Sbjct  535  YWKSND-----------NTDVSGARREKLRLSNADVSTRTIAPSL  568

 Score = 45.9 bits (107),  Expect = 0.009
 Identities = 28/164 (17%), Positives = 54/164 (32%), Gaps = 14/164 (8%)

Query  180  DGEVVRSCKQNNVAVGASCMYAGDLKA-GANEVITGMRLTKVGNAIWPEIQVAKVTLFGL  238
            D  V          +         +   D+       N  V+TG+R    VG    + E   +
Sbjct  466  DRHVKNGVDYHTLSYEKRTIDLDDVTDENSFVVTGVRFRVVGTHLNLEA------YYSE  519

Query  239  IDKSTRKWVGPGGKMKSLCAPIKSLVAAEPTRLPAGHVLTQVQLVGTTKKNVKVGGQKLN  298
             D  T +  P                      A   +  L   + + TT + ++  Q ++
Sbjct  520  FDFRTGQLIQPEYNSYWKSNDNTDVSGARREKLRLSNADVSTRTIAPSLP-LSRHNQYID  578

Query  299  FYTGKLDKPKTAPAKKVVKLNDLVSDIEPVYVSTNVAGAGLRY  342
            F   LDK          +   + D V S   P           +G  G+ Y
Sbjct  579  FTNTGLDKDAAQSTVPFMDIQDVVSNPPVP------LAGIGIY  616

>ref|XP_002427759.1| G conserved hypothetical protein [Pediculus humanus corporis]
 gb|EEB15021.1| G conserved hypothetical protein [Pediculus humanus corporis]
Length=635

 GENE ID: 8231845 Phum_PHUM343650 | hypothetical protein
[Pediculus humanus corporis]

                                                   Sort alignments for this subject
                                                     E value  Score  Percent identi
                                                     Query start position  Subject

 Score = 71.3 bits (173),  Expect = 2e-10
 Identities = 24/115 (21%), Positives = 47/115 (41%), Gaps = 14/115 (12%)

Query  209  NEVITGMRLTKVGNAIWPEIQVAKVTLFGLIDKSTRKWVGP-----------GGKMKSL  256
            N+V+TG+R  K     I   +IQ  ++   G I++  T  W
Sbjct  415  NKVVTGIRLVKHNRIIHIQIQQGSLIPRGIINNGTLSWKPVDVNYTIDDLNVENNVDYHTM  474

Query  257  CAPIKSLVAAEPTRLPAGHVLTQVQL-VGTTKKNVKVGGQKLNFYTGKLDKPKTA  310
                 +    +   +   G+  V+T V++     +  N+    TG+++  P+
Sbjct  475  TWAERT-IDMDDVFGPSEHAVTGVRFRKLGSHLNLEIRVTPFNFTTGQVSSTDKS  528

 Score = 38.5 bits (88),  Expect = 1.5
 Identities = 14/65 (22%), Positives = 28/65 (44%), Gaps = 3/65 (4%)

Query  174  ENRFGNDGEVVRSCKQNNVAVGASCMYAGDLKAGANEVITGMRLTKVGNAIWPEIQVAKV  233
            +N   +D V    +        +     M   D+   +  + V+TG+R    K+G+ +   EI   +V
Sbjct  456  DNYTIDDLNVENNVDYHTMTWAERTIDMDDVFGPSEHAVTGVRFRKLGSHLNLEI---RV  512

Query  234  TLFGL  238
            T F
Sbjct  513  TPFNF  517

>gb|EFN73534.1|  hypothetical protein EAG_09552 [Camponotus floridanus]
Length=668

                                                   Sort alignments for this subject
                                                     E value  Score  Percent identi
                                                     Query start position  Subject

 Score = 70.9 bits (172),  Expect = 3e-10
 Identities = 27/113 (24%), Positives = 49/113 (44%), Gaps = 14/113 (12%)

Query  208  ANEVITGMRLTKVGNAIWPEIQVAKVTLFGLIDKSTRKWVGPGG-----------KMKS  255
             N VITG+RL K+     I  +IQ  ++   G I+ ST  +W
Sbjct  433  NNRVITGIRLKKINKIHIQIQEGQLLPRGAINTSTTEWQPVDAFSISDFNVKSDIDYHR  492

Query  256  LCAPIKSLVAAEPTRLPAGHVLTQVQL-VGTTKKNVKVGGQKLNFYTGKLDKP  307
            L      ++L       +       H+LT ++  +       N++     NF    +G L  +P
Sbjct  493  LVWEKRAL-DLDDLQSSQDHLLTGIRFRMVGSRLNLEIMVTPFNFTSGLLIQP  544

 Score = 39.3 bits (90),  Expect = 0.85
 Identities = 12/52 (24%), Positives = 24/52 (47%), Gaps = 0/52 (0%)

Query  179  NDGEVVRSCKQNNVAVGASCMYAGDLKAGANEVITGMRLTKVGNAIWPEIQV  230
            +D  V           +         DL++  + +++TG+R    VG+ +  EI V
Sbjct  480  SDFNVKSDIDYHRLVWEKRALDLDDLQSSQDHLLTGIRFRMVGSRLNLEIMV  531

>ref|XP_001978593.1| G GG17594 [Drosophila erecta]
 gb|EDV47520.1| G GG17594 [Drosophila erecta]
Length=844

 GENE ID: 6551613 Dere\GG17594 | GG17594 gene product from transcript GG17594-RA
[Drosophila erecta] (10 or fewer PubMed links)

                                                   Sort alignments for this subject
                                                     E value  Score  Percent identi
                                                     Query start position  Subject

 Score = 70.9 bits (172),  Expect = 3e-10
 Identities = 37/158 (24%), Positives = 61/158 (39%), Gaps = 25/158 (15%)

Query  209  NEVITGMRLTKVGNAIWPEIQVAKVTLFGLIDKSTRKWVGP-----------GGKMKSL  256
            N V+TG+R  K          +Q    G +++ST +W                              G     L
Sbjct  617  NRVVTGLRFVKQNRIFHLQIQEGELLPRGTVNQSTLEWKPVEKYNVKPENFDRHVKNGVDYHTL  676

Query  257  CAPIKSLVAAEPTRLP-AGHVLTQVQL-VGTTKKNVKVGGQKLNFYTGKLDKPKTAPAKK  314
            ++    +   +     V     T   N++   +  TG+L  P+
Sbjct  677  SYENRT-IDLDDVTDENSFVVTGVRFRVIGTHLNLEAYYSEFDFRTGQLIQP---EGNS  732

Query  315  VVKLNDLVSVD-IEPVYVSTNVAGAGLRYDVETKTITP  351
             K ND      V       ++ N A       DV  T+TI P
Sbjct  733  YWKSNDNTDVSGTRRQKLNLNNA------DVSTRTIAP  764

 Score = 46.2 bits (108),  Expect = 0.007
 Identities = 25/164 (15%), Positives = 54/164 (32%), Gaps = 14/164 (8%)

Query  180  DGEVVRSCKQNNVAVGASCMYAGDLKA-GANEVITGMRLTKVGNAIWPEIQVAKVTLFGL  238
            D  V          +         +   D+       N  V+TG+R    +G    + E   +
Sbjct  664  DRHVKNGVDYHTLSYENRTIDLDDVTDENSFVVTGVRFRVIGTHLNLEA------YYSE  717

Query  239  IDKSTRKWVGPGGKMKSLCAPIKSLVAAEPTRLPAGHVLTQVQLVGTTKKNVKVGGQKLN  298
             D  T +  P G                    +   +  +    +       +      Q ++
Sbjct  718  FDFRTGQLIQPEGNSYWKSNDNTDVSGTRRQKLNLNNADVSTRTIAPSIP-LSRHNQYID  776

Query  299  FYTGKLDKPKTAPAKKVVKLNDLVSDIEPVYVSTNVAGAGLRY  342
            F    +DK          +   + D ++S   P           +G  G+ Y
Sbjct  777  FTNTGMDKDAAQSTVPFIDIQDVISNPPVP------LAGIGIY  814

>ref|XP_001963941.1| G GF20993 [Drosophila ananassae]
 gb|EDV34390.1| G GF20993 [Drosophila ananassae]
Length=851

 GENE ID: 6503682 Dana\GF20993 | GF20993 gene product from transcript GF20993-RA
[Drosophila ananassae] (10 or fewer PubMed links)

                                                   Sort alignments for this subject
                                                     E value  Score  Percent identi
                                                     Query start position  Subject

 Score = 70.5 bits (171),  Expect = 3e-10
 Identities = 27/113 (24%), Positives = 48/113 (43%), Gaps = 15/113 (13%)

Query  209  NEVITGMRLTKVGNAIWPEIQVAKVTLFGLIDKSTRKWVG-----------PGGKMKSL  256
            N V+TG+R  K          +Q    G I+++ST +W                              G    L
Sbjct  623  NRVVTGLRLVKSNRIFHLQIQEGELMPRGVINQSTLEWRPVEKYNIFDRDVKKGVDYHTL  682

Query  257  CAPIKSLVAAEPTRL-PAGHVLTQVQL-VGTTKKNVKVGGQKLNFYTGKLDKP  307
            ++    +      +     V+T V+      ++++     +L+   TG+L  P
Sbjct  683  SYESRS-IDLDDVNTDDNSFVVTGVRFRLVGAHLNLEAYFTEFDFKTGKLIQP  734

 Score = 51.6 bits (122),  Expect = 2e-04
 Identities = 36/172 (21%), Positives = 59/172 (35%), Gaps = 29/172 (16%)

Query  180  DGEVVRSCKQNNVAVGASCMYAGDLKAGANE-VITGMRLTKVGNAIWPEIQVAKVTLFGL  238
            D +V +            +       +   D+     N + V+TG+R    VG    + E
Sbjct  670  DRDVKKGVDYHTLSYESRSIDLDDVNTDDNSFVVTGVRFRLVGAHLNLEAYFTEFDFKTG  729

Query  239  IDKSTRKWVGPGGKMKSLCAPIKSL--------VAAEPTRLPAGHVLTQVQLVGTTKKNV  290
             D  T K  P                            T LP       + T  + Q           LV
Sbjct  730  KLIQPEINSYWKSNDNTDVSGGARREKLRLANTDLPTRTIAT--SLVQSRHN--  779

Query  291  KVGGQKLNFYTGKLDKPKTAPAKKVVKLNDLVSDIEPVYVSTNVAGAGLRY  342
                Q ++F    +DK          +   + D VS  P  +S    G+ Y
Sbjct  780  ----QYIDFTNTGLDKDAAQSTVPFIDIQDVVS--NPPVPLS--GIGIY  821

>ref|NP_996366.1| UG lethal (1) G0193, isoform B [Drosophila melanogaster]
 gb|AAS65275.1| G lethal (1) G0193, isoform B [Drosophila melanogaster]
Length=646

 GENE ID: 31712 l(1)G0193 | lethal (1) G0193 [Drosophila melanogaster]
(Over 10 PubMed links)

                                                   Sort alignments for this subject
                                                     E value  Score  Percent identi
                                                     Query start position  Subject

 Score = 70.5 bits (171),  Expect = 3e-10
 Identities = 26/113 (24%), Positives = 46/113 (41%), Gaps = 15/113 (13%)

Query  209  NEVITGMRLTKVGNAIWPEIQVAKVTLFGLIDKSTRKWVGP-----------GGKMKSL  256
            N V+TG+R  K          +Q    G++++ST +W                              G    L
Sbjct  419  NRVVTGLRFVKQNRIFHLQIQEGELLPRGVINQSTLEWKPVEKYNVKPENFDRHVKNGVDYHTL  478

Query  257  CAPIKSLVAAEPTRL-PAGHVLTQVQL-VGTTKKNVKVGGQKLNFYTGKLDKP  307
            ++    +      V         V+T V+     ++++     +L+   TG+L  P
Sbjct  479  SYE-KRTIDLDDVTDENSFVVTGVRFRVVGTHLNLEAYYSEFDFRTGQLIRP  530

 Score = 48.2 bits (113),  Expect = 0.002
 Identities = 29/164 (18%), Positives = 54/164 (33%), Gaps = 14/164 (8%)

Query  180  DGEVVRSCKQNNVAVGASCMYAGDLKA-GANEVITGMRLTKVGNAIWPEIQVAKVTLFGL  238
            D  V          +         +   D+       N  V+TG+R    VG    + E   +
Sbjct  466  DRHVKNGVDYHTLSYEKRTIDLDDVTDENSFVVTGVRFRVVGTHLNLEA------YYSE  519

Query  239  IDKSTRKWVGPGGKMKSLCAPIKSLVAAEPTRLPAGHVLTQVQLVGTTKKNVKVGGQKLN  298
             D  T +  P                      A   +  L   + + TT + ++  Q ++
Sbjct  520  FDFRTGQLIRPEYNSYWKSNDNTDVSGARREKLRLSNADVSTRTIAHSIP-LSRHNQYID  578

Query  299  FYTGKLDKPKTAPAKKVVKLNDLVSDIEPVYVSTNVAGAGLRY  342
            F    LDK          +   + D VVS   P           +G  G+ Y
Sbjct  579  FTNTGLDKDAAQSTVPFMDIQDVVSNPPVP------LAGIGIY  616

>ref|NP_572427.1| UG lethal (1) G0193, isoform A [Drosophila melanogaster]
 gb|AAF46301.1| G lethal (1) G0193, isoform A [Drosophila melanogaster]
 gb|AAL13770.1| G LD24308p [Drosophila melanogaster]
 gb|ACL84971.1|  l(1)G0193-PA [synthetic construct]
Length=664

 GENE ID: 31712 l(1)G0193 | lethal (1) G0193 [Drosophila melanogaster]
(Over 10 PubMed links)

                                                   Sort alignments for this subject
                                                     E value  Score  Percent identi
                                                     Query start position  Subject

 Score = 70.5 bits (171),  Expect = 3e-10
 Identities = 26/113 (24%), Positives = 46/113 (41%), Gaps = 15/113 (13%)

Query  209  NEVITGMRLTKVGNAIWPEIQVAKVTLFGLIDKSTRKWVGP-----------GGKMKSL  256
            N V+TG+R  K          +Q    G++++ST +W                              G    L
Sbjct  437  NRVVTGLRFVKQNRIFHLQIQEGELLPRGVINQSTLEWKPVEKYNVKPENFDRHVKNGVDYHTL  496

Query  257  CAPIKSLVAAEPTRL-PAGHVLTQVQL-VGTTKKNVKVGGQKLNFYTGKLDKP  307
            ++    +      V         V+T V+     ++++     +L+   TG+L  P
Sbjct  497  SYE-KRTIDLDDVTDENSFVVTGVRFRVVGTHLNLEAYYSEFDFRTGQLIRP  548

 Score = 48.2 bits (113),  Expect = 0.002
 Identities = 29/164 (18%), Positives = 54/164 (33%), Gaps = 14/164 (8%)

Query  180  DGEVVRSCKQNNVAVGASCMYAGDLKA-GANEVITGMRLTKVGNAIWPEIQVAKVTLFGL  238
            D  V          +         +   D+       N  V+TG+R    VG    + E   +
Sbjct  484  DRHVKNGVDYHTLSYEKRTIDLDDVTDENSFVVTGVRFRVVGTHLNLEA------YYSE  537

Query  239  IDKSTRKWVGPGGKMKSLCAPIKSLVAAEPTRLPAGHVLTQVQLVGTTKKNVKVGGQKLN  298
             D  T +  P                      A   +  L   + + TT + ++  Q ++
Sbjct  538  FDFRTGQLIRPEYNSYWKSNDNTDVSGARREKLRLSNADVSTRTIAHSIP-LSRHNQYID  596

Query  299  FYTGKLDKPKTAPAKKVVKLNDLVSDIEPVYVSTNVAGAGLRY  342
            F    LDK          +   + D VVS   P           +G  G+ Y
Sbjct  597  FTNTGLDKDAAQSTVPFMDIQDVVSNPPVP------LAGIGIY  634

>gb|EFN89744.1|  hypothetical protein EAI_01992 [Harpegnathos saltator]
Length=641

                                                   Sort alignments for this subject
                                                     E value  Score  Percent identi
                                                     Query start position  Subject

 Score = 70.1 bits (170),  Expect = 5e-10
 Identities = 32/137 (24%), Positives = 58/137 (43%), Gaps = 15/137 (10%)

Query  187  CKQNNVAVGAS-CMYAGDLKAGANEVITGMRLTKVGNAIWPEIQVAKVTLFGLIDKSTR  245
            C  +N +           +  A   N++ITG RL KV    I  +IQ  ++   G I+ S+
Sbjct  386  CDDHNSSSDRYFSLKAVKSDAANNKIITGIRLRKVNQIIHIQIQEGKLLPRGGINASSIH  445

Query  246  WVGP-----------GGKMKSLCAPIKSLVAAEPTRLPAGHVLTQVQL-VGTTKKNVKV  292
            W          G  +     K  +  + P    H+LT ++  +  ++  N+++
Sbjct  446  WKPIDEFSIMSSDVRNGVEYHKLVWE-KRAMDLDDLLPPEDHLLTGIRFRMVGSRLNLEI  504

Query  293  GGQKLNFYTGKLDKPKT  309
                NF  +G L+  P+
Sbjct  505  RATPFNFTSGLLNEPEK  521

 Score = 35.8 bits (81),  Expect = 9.4
 Identities = 13/57 (23%), Positives = 24/57 (43%), Gaps = 3/57 (5%)

Query  182  EVVRSCKQNNVAVGASCMYAGDLKAGANEVITGMRLTKVGNAIWPEIQVAKVTLFGL  238
            +V          M   DL       +++TG+R    VG+ +  EI    + T F
Sbjct  458  DVRNGVEYHKLVWEKRAMDLDDLLPPEDHLLTGIRFRMVGSRLNLEI---RATPFNF  511

>gb|EFA12872.1|  hypothetical protein TcasGA2_TC010724 [Tribolium castaneum]
Length=1530

                                                   Sort alignments for this subject
                                                     E value  Score  Percent identi
                                                     Query start position  Subject

 Score = 69.7 bits (169),  Expect = 6e-10
 Identities = 33/138 (24%), Positives = 51/138 (37%), Gaps = 16/138 (11%)

Query  187  CKQNNVAVGASCMYAGDLKAG--ANEVITGMRLTKVGNAIWPEIQVAKVTLFGLIDKSTR  244
            C++  V+     +   KA    N V+TG+R  K      +  IQ  K+   G +D  T
Sbjct  178  CEEQGVSYSERYVNLRKITKANISDNRVVTGLRFIEKIKHNQIVHLIHIQEGKLLEKGYVDPKTV  237

Query  245  KWVGP-----------GGKMKSLCAPIKSLVAAEPTRLPAGHVLTQVQLVGT-TKKNV  291
             +WV P                        +L              V+ I  V       +  GG   K
Sbjct  238  QWVPPDNYKIDDRNIYEEEDYHTLSWEERSL-ELTEIVAEEGSVVIGVRFKKTLGGVKLE  296

Query  292  VGGQKLNFYTGKLDKPKT  309
              V       +F  TGKL+
Sbjct  297  VLTTTFDFDTGKLEDNTT  314

 Score = 35.8 bits (81),  Expect = 9.4
 Identities = 21/164 (13%), Positives = 46/164 (29%), Gaps = 21/164 (12%)

Query  179  NDGEVVRSCKQNNVAVGASCMYAGDLKAGANEVITGMRLTKVGNAIWPEIQVAKVTLFGL  238
            +D  +        + ++          ++  A        V+ G+R  K    E+
```

```
Sbjct  248  DDRNIYEEEDYHTLSWEERSLELTEIVAEEGSVVIGVRFKKTLGGVKLEVLTTTFDFDTG  307

Query  239  IDKSTRKWVGPGGKMKSLCAPIKSLVAAEPTRLPAGHVLTQVQLVGTTKKNVKVGGQKLN  298
              +     W          I    +  +    +P     L  +     +        +
Sbjct  308  KLEDNTTWNIFKNTITPN--LINQEIKLDSPDIPIHKNLASIDF--SENTYINFTNTDYD  363

Query  299  FYTGKLDKPKTAPAKKVVKLNDLVSVDIEPVY--VSTNVAGAGL  340
              G+   P                   D +PVY     T ++G GL
Sbjct  364  KDAGQTTVPVFF---------------DAQPVYSTAPTPLSGIGL  392
```

>**ref|XP_967785.2|** GM PREDICTED: similar to conserved hypothetical protein [Tribolium castaneum]
Length=519

GENE ID: 656143 LOC656143 | similar to CG2206-PA, isoform A [Tribolium castaneum]

Sort alignments for this subject
E value **Score** **Percent identi**
Query start position **Subject**

Score = 69.7 bits (169), Expect = 6e-10
Identities = 33/138 (24%), Positives = 51/138 (37%), Gaps = 16/138 (11%)

```
Query  187  CKQNNVAVGASCMYAGDLKAG--ANEVITGMRLTKVGNAIWPEIQVAKVTLFGLIDKSTR  244
            C++  V+      +      KA     N V+TG+R  K     +    IQ  K+    G +D  T
Sbjct  102  CEEGQVSVSYERYVNLRITKANISDNRVVTGLRFIKHNQIVHLHIQEGKLLEKGYVDPKTV  161

Query  245  KWVGP-----------GGKMKSLCAPIKSLVAAEPTRLPAGHVLTQVQLVGT-TKKNVK  291
            +WV P                    +L         +SL         G V+   V+   T    ++
Sbjct  162  QWVPPDNYKIDDRNIYEEEDYHTLSWEERSL-ELTEIVAEEGSVVIGVRFKKTLGGVKLE  220

Query  292  VGGQKLNFYTGKLDKPKT  309
            V      +F TGKL+    T
Sbjct  221  VLTTTFDFDTGKLEDNTT  238
```

Score = 35.8 bits (81), Expect = 9.4
Identities = 21/164 (13%), Positives = 46/164 (29%), Gaps = 21/164 (12%)

```
Query  179  NDGEVVRSCKQNNVAVGASCMYAGDLKAGANEVITGMRLTKVGNAIWPEIQVAKVTLFGL  238
            +D  +          + ++       +       ++ A      V+ G+R   K          +  E+
Sbjct  172  DDRNIYEEEDYHTLSWEERSLELTEIVAEEGSVVIGVRFKKTLGGVKLEVLTTTFDFDTG  231

Query  239  IDKSTRKWVGPGGKMKSLCAPIKSLVAAEPTRLPAGHVLTQVQLVGTTKKNVKVGGQKLN  298
              +     W          I    +  +    +P     L  +     +        +
Sbjct  232  KLEDNTTWNIFKNTITPN--LINQEIKLDSPDIPIHKNLASIDF--SENTYINFTNTDYD  287

Query  299  FYTGKLDKPKTAPAKKVVKLNDLVSVDIEPVY--VSTNVAGAGL  340
              G+   P                   D +PVY     T ++G GL
Sbjct  288  KDAGQTTVPVFF---------------DAQPVYSTAPTPLSGIGL  316
```

>**ref|XP_001810077.1|** UGM PREDICTED: similar to GA15301-PA [Tribolium castaneum]
Length=426

GENE ID: 100142514 LOC100142514 | similar to GA15301-PA [Tribolium castaneum]

Sort alignments for this subject
E value **Score** **Percent identi**
Query start position **Subject**

Score = 69.0 bits (167), Expect = 1e-09
Identities = 30/136 (23%), Positives = 54/136 (40%), Gaps = 16/136 (11%)

```
Query  187  CKQNNVAVGASCMYAGDLKAG--ANEVITGMRLTKVGNAIWPEIQVAKVTLFGLIDKSTR  244
            C +     +     +          N V+TG+R  K     +    ++Q  K+    G I+ +T
Sbjct  176  CDEGQGSYSDRYINLRISMSNISDNRVVTGLRFIKHNRIVHLQVQEGKLLEKGNINPTTV  235

Query  245  KWVGP-----------GGKMKSLCAPIKSLVAAEPTRLPAGHVLTQVQLVGT-TKKNVK  291
            +WV P                    G    ++       ++ +             AG V+T V+     T       N++
Sbjct  236  QWVPPEDYKITDRDIYKGQDYHTISWEERT-IELTKVIAKAGSVVTGVRFKRTFGSLNLQ  294

Query  292  VGGQKLNFYTGKLDKP  307
            V      +F TGKL+K
Sbjct  295  VLTTAFDFNTGKLEKN  310
```

Score = 36.2 bits (82), Expect = 7.2
Identities = 13/98 (14%), Positives = 34/98 (35%), Gaps = 3/98 (3%)

```
Query  179  NDGEVVRSCKQNNVAVGASCMYAGDLKAGANEVITGMRLTKVGNAIWPEIQVAKVTLF--  236
             D ++ +          + ++       +      A A    V+TG+R     +    ++
Sbjct  246  TDRDIYKGQDYHTISWEERTIELTKVIAKAGSVVTGVRFKRTFGSLNLQVLTTAFDFNTG  305

Query  237  GLIDKSTRKW-VGPGGKMKSLCAPIKSLVAAEPTRLPA  273
             L    S     W +          ++       +     V      P+ +P
Sbjct  306  KLEKNSDSDWGIFESAPYFNIRHKFRLKVNLNPSDVPV  343
```

>**ref|XP_002101210.1|** G GE17494 [Drosophila yakuba]
gb|EDX02318.1| G GE17494 [Drosophila yakuba]
Length=844

GENE ID: 6525374 Dyak\GE17494 | GE17494 gene product from transcript GE17494-RA [Drosophila yakuba] (10 or fewer PubMed links)

Sort alignments for this subject
E value **Score** **Percent identi**
Query start position **Subject**

Score = 68.6 bits (166), Expect = 1e-09
Identities = 26/113 (24%), Positives = 45/113 (40%), Gaps = 15/113 (13%)

```
Query  209  NEVITGMRLTKVGNAIWPEIQVAKVTLFGLIDKSTRKWVGP------------GGKMKSL  256
            N ++TG+R  K         +IQ  ++    G +++ST +W                          G       L
Sbjct  617  NRIVTGLRFVKQNRIFHLQIQEGELLPRGTVNQSTLEWKPVEKYNIFDRHVKNGIDYHKL  676

Query  257  CAPIKSLVAAEPTRLP-AGHVLTQVQL-VGTTKKNVKVGGQKLNFYTGKLDKP  307
                K    +                   V+T V+  V    T  N++        + +F TGKL +P
Sbjct  677  SYE-KRTIDLDDVDTEDNSFVITGVRFRVVGTHLNLEAYYSEFDFRTGKLIQP  728
```

Score = 51.2 bits (121), Expect = 2e-04
Identities = 31/164 (19%), Positives = 54/164 (33%), Gaps = 14/164 (8%)

```
Query  180  DGEVVRSCKQNNVAVGASCMYAGDLKA-GANEVITGMRLTKVGNAIWPEIQVAKVTLFGL  238
            D  V          + ++       D+          + VITG+R    VG    +    E              +
Sbjct  664  DRHVKNGIDYHKLSYEKRTIDLDDVDTEDNSFVITGVRFRVVGTHLNLEA------YYSE  717

Query  239  IDKSTRKWVGPGGKMKSLCAPIKSLVAAEPTRLPAGHVLTQVQLVGTTKKNVKVGGQKLN  298
             D  T K + P                 +     A     +L      +             +    V +           Q ++
Sbjct  718  FDFRTGKLIQPEANSYWKSNDNTDVSGARREKLSLNNADVSTRTVAHSIP-LSRHNQYID  776

Query  299  FYTGKLDKPKTAPAKKVVKLNDLVSVDIEPVYVSTNVAGAGLRY  342
            F     LDK            + + D+VS       P         +AG G+ Y
Sbjct  777  FTNTGLDKDAAQSTVPFIDIQDVVSNPPVP------LAGIGIYY  814
```

>**ref|XP_001946173.1|** UGM PREDICTED: similar to GA15301-PA [Acyrthosiphon pisum]
Length=677

GENE ID: 100162921 LOC100162921 | similar to GA15301-PA [Acyrthosiphon pisum]

Sort alignments for this subject
E value **Score** **Percent identi**
Query start position **Subject**

Score = 68.6 bits (166), Expect = 1e-09
Identities = 30/138 (22%), Positives = 56/138 (41%), Gaps = 19/138 (13%)

```
Query  208  ANEVITGMRLTKVGNAIWPEIQVAKVTLFGLIDKSTRKWVG-PGGKMKSLCAPIKS----  262
             N VITG+R  KV          + Q  ++    G I +S+R+W+  PG +          +K
Sbjct  445  NNMVITGVRFIKVKQMFHLQAQEGELGPRGGISESSRQWLEVPGSEFSYKNKTLKDGVDY  504

Query  263  --------LVAAEPTRLPAGHVLTQVQLVG-TTKKNVKVGGQKLNFYTGKLDKPK-----  308
                    ++  +      P     V+T ++         + N++V         +F TG+L +P+
Sbjct  505  HTLTYESRIIDIDNVIAPENTVVTGLKFRKIGSHLNIEVRITPFDFMTGELIEPELKSYW  564

Query  309  TAPAKKVVKLNDLVSVDI  326
                       + +N      +DI
Sbjct  565  IGNENTELSINPRTEIDI  582
```

Score = 43.5 bits (101), Expect = 0.045
Identities = 15/91 (17%), Positives = 38/91 (42%), Gaps = 11/91 (12%)

```
Query  188  KQNNVAVGASCMYAGDLKAGANEVITGMRLTKVGNAIWPEIQVAKVTLFGLIDKSTRK--  245
              +  +    +         ++ A   N V+TG++   K+G+     +  E+        ++T  F         +
Sbjct  503  DYHTLTYESRIIDIDNVIAPENTVVTGLKFRKIGSHLNIEV---RITPFDFMTGELIEPE  559

Query  246  ----WVGPGGKMKSLCAPIKSLVAAEPTRLP  272
                W+    G +      L         ++  +         + LP
Sbjct  560  LKSYWI--GNENTELSINPRTEIDINNSDLP  588
```

>**ref|XP_002057094.1|** G GJ16894 [Drosophila virilis]
gb|EDW62580.1| G GJ16894 [Drosophila virilis]
Length=824

GENE ID: 6633805 Dvir\GJ16894 | GJ16894 gene product from transcript GJ16894-RA [Drosophila virilis] (10 or fewer PubMed links)

Sort alignments for this subject
E value **Score** **Percent identi**
Query start position **Subject**

Score = 67.0 bits (162), Expect = 4e-09
Identities = 32/134 (24%), Positives = 53/134 (40%), Gaps = 19/134 (14%)

```
Query  209  NEVITGMRLTKVGNAIWPEIQVAKVTLFGLIDKSTRKW------------VGPGGKMKSL  256
            N+VITG+R  K         +IQ  ++    G I++ST  W                   V  G      +L
Sbjct  596  NKVITGLRFLKKNRIFHLQIQEGQLLPRGAIESTLSWKPVDEYKIFDRNVAKGIDYHTL  655

Query  257  CAPIKSLVAAEPTRLPAGH--VLTQVQL-VGTTKKNVKVGGQKLNFYTGKLDKPKTAPAK  313
              +S  +   +             V+T V+  V    T  N++        + +F  G+L +P
Sbjct  656  SYESRS-IDLDDIMKTDDNSFVVTGVRFRVLGAHLNLEARLSEFDFKKGELIQP---EVN  711

Query  314  KVVKLNDLVSVDIE  327
               V + ND    V  E
Sbjct  712  SVWQSNDNTDVSGE  725
```

Score = 53.2 bits (126), Expect = 6e-05
Identities = 31/168 (19%), Positives = 58/168 (35%), Gaps = 21/168 (12%)

```
Query  180  DGEVVRSCKQNNVAVGASCMYAGDLKA--GANEVITGMRLTKVGNAIWPEIQVAKVTLFG  237
            D  V +          + ++    +       D+      + V+TG+R     +G    +    E ++++
Sbjct  643  DRNVAKGIDYHTLSYESRSIDLDDIMKTDDNSFVVTGVRFRVLGAHLNLEARLSEFDFKK  702

Query  238  --LIDKSTRK-WVGPGGKMKSLCAPIKSLVAAEPTRLPAGHVLTQVQLVGTTKKNVKVGG  294
               LI          W             S         K    L                +P       V+T  L +
Sbjct  703  GELIQPEVNSVWQSNDNTDVSGERRQKLL--LYNADVPTRTVVTSLPLSKHN--------  752

Query  295  QKLNFYTGKLDKPKTAPAKKVVKLNDLVSVDIEPVYVSTNVAGAGLRY  342
            Q ++F         +DK                   + + D+VS       P            +AG G+ Y
Sbjct  753  QYIDFVNSGMDKDAAQSTVPFLDIQDVVSQPPVP------LAGIGIYY  794
```

>**ref|XP_002010644.1|** G GI21656 [Drosophila mojavensis]
gb|EDW06299.1| G GI21656 [Drosophila mojavensis]
Length=727

GENE ID: 6585004 Dmoj\GI21656 | GI21656 gene product from transcript GI21656-RA [Drosophila mojavensis] (10 or fewer PubMed links)

Score = 67.0 bits (162), Expect = 4e-09
Identities = 30/134 (23%), Positives = 52/134 (39%), Gaps = 19/134 (14%)

```
Query  209  NEVITGMRLTKVGNAIWPEIQVAKVTLFGLIDKSTRKW------------VGPGGKMKSL  256
            N V+TG+R  K         +IQ  ++    G I++ST  W                   V  G      +L
Sbjct  499  NNVVTGLRFVKRNRFFHLQIQEGQLLPRGAINESTLSWKPVDEYKIYDRNVAKGIDYHTL  558

Query  257  CAPIKSLVAAEPTRLPAGH--VLTQVQL-VGTTKKNVKVGGQKLNFYTGKLDKPKTAPAK  313
              +S  +   +             V+T V+  V    T  N++        + +F  G+L +P
Sbjct  559  SYESRS-IDLDDIMKTDDYSFVVTGVRFRVLGAHLNLEARLTEFDFTKGELIQP---EVN  614

Query  314  KVVKLNDLVSVDIE  327
               V + ND    V  E
Sbjct  615  SVWQSNDNTDVSGE  628
```

>**ref|XP_002092594.1|** G GE14278 [Drosophila yakuba]
gb|EDW92306.1| G GE14278 [Drosophila yakuba]
Length=678

GENE ID: 6531807 Dyak\GE14278 | GE14278 gene product from transcript GE14278-RA [Drosophila yakuba] (10 or fewer PubMed links)

Score = 66.7 bits (161), Expect = 5e-09
Identities = 25/115 (22%), Positives = 50/115 (44%), Gaps = 15/115 (13%)

```
Query  209  NEVITGMRLTKVGNAIWPEIQVAKVTLFGLIDKSTRKWVG------------PGGKMKSL  256
            N+V+TG+R  K+         ++Q  ++    G I  S+ +W+                          G      +L
Sbjct  434  NKVVTGVRFVKIKRVFHLQLQQGELLPGGVIKNSSLEWLPVEAYNVSDVDIRDGYDYHTL  493

Query  257  CAPIKSLVAAEPTRL-PAGHVLTQVQLVGTTKKNVKVGGQKLNFYTGKLDKPKT  309
              +S  +   +                V+T V+  V     N++        + NF TG+ L +P
Sbjct  494  SVDSRAM-DLDEISVNTTDQVVTGFRFRVFKKHLNLEVRFTSFNFTSGQLIEPQT  547
```

>**ref|XP_001355344.2|** G GA15301 [Drosophila pseudoobscura pseudoobscura]
gb|EAL32401.2| GA15301 [Drosophila pseudoobscura pseudoobscura]
Length=806

GENE ID: 4816053 Dpse\GA15301 | GA15301 gene product from transcript GA15301-RA [Drosophila pseudoobscura pseudoobscura] (10 or fewer PubMed links)

Sort alignments for this subject
E value **Score** **Percent identi**
Query start position **Subject**

Score = 66.3 bits (160), Expect = 7e-09
Identities = 40/158 (26%), Positives = 63/158 (40%), Gaps = 24/158 (15%)

```
Query  209  NEVITGMRLTKVGNAIWPEIQVAKVTLFGLIDKSTRKWVG------------PGGKMKSL  256
            N V+TG+R  K         +IQ  ++    G I+ + T  W+                          G       L
Sbjct  576  NRVVTGLRFVKRNRIFHLQIQEGELLPRGGINHTTLSWKPVENYNIFDRDVKKGTDYHTL  635

Query  257  CAPIKSLVAAEPTRL-PAGHVLTQVQLVGTTKKNVKVGGQKLNFYTGKLDKPKTAPAKK  314
              +S  V               V+T V+  V     N++        + +F TG+L +P    A
Sbjct  636  SYESRS-VDLDDVNTDDNSFVVTGVRFRVVGAHLNLEACYSEFDFKTGQLLQP---EANS  691

Query  315  VVKLNDL--VSVDIEPVYVSTNVAGAGLRYDVETKTIT  350
              K ND    VS  D  +          +++ A         DV T+TIT
Sbjct  692  YWKSNDNTDVSGDSKTRREKVSLSNA----DVSTRTIT  725
```

Score = 46.2 bits (108), Expect = 0.007
Identities = 11/50 (22%), Positives = 22/50 (44%), Gaps = 1/50 (2%)

```
Query  180  DGEVVRSCKQNNVAVGASCMYAGDLKAGANE-VITGMRLTKVGNAIWPEI  228
            D +V +         + ++      +       D+       N V+TG+R    VG    +
Sbjct  623  DRDVKKGTDYHTLSYESRSVDLDDVNTDDNSFVVTGVRFRVVGAHLNLEA  672
```

>**ref|XP_002025529.1|** G GL15246 [Drosophila persimilis]
gb|EDW31051.1| G GL15246 [Drosophila persimilis]
Length=806

GENE ID: 6600424 Dper\GL15246 | GL15246 gene product from transcript GL15246-RA [Drosophila persimilis] (10 or fewer PubMed links)

Sort alignments for this subject
E value **Score** **Percent identi**
Query start position **Subject**

Score = 66.3 bits (160), Expect = 7e-09
Identities = 40/158 (26%), Positives = 63/158 (40%), Gaps = 24/158 (15%)

```
Query  209  NEVITGMRLTKVGNAIWPEIQVAKVTLFGLIDKSTRKWVG------------PGGKMKSL  256
            N V+TG+R  K         +IQ  ++    G I+ + T  W+                          G       L
Sbjct  576  NRVVTGLRFVKRNRIFHLQIQEGELLPRGGINHTTLSWKPVENYNIFDRDVKKGTDYHTL  635

Query  257  CAPIKSLVAAEPTRL-PAGHVLTQVQLVGTTKKNVKVGGQKLNFYTGKLDKPKTAPAKK  314
              +S  V               V+T V+  V     N++        + +F TG+L +P    A
Sbjct  636  SYESRS-VDLDDVNTDDNSFVVTGVRFRVVGAHLNLEACYSEFDFKTGQLLQP---EANS  691

Query  315  VVKLNDL--VSVDIEPVYVSTNVAGAGLRYDVETKTIT  350
              K ND    VS  D  +          +++ A         DV T+TIT
Sbjct  692  YWKSNDNTDVSGDSKTRREKVSLSNA----DVSTRTIT  725
```

Score = 46.2 bits (108), Expect = 0.007
Identities = 11/50 (22%), Positives = 22/50 (44%), Gaps = 1/50 (2%)

```
Query  180  DGEVVRSCKQNNVAVGASCMYAGDLKAGANE-VITGMRLTKVGNAIWPEI  228
            D +V +         + ++      +       D+       N V+TG+R    VG    +
Sbjct  623  DRDVKKGTDYHTLSYESRSVDLDDVNTDDNSFVVTGVRFRVVGAHLNLEA  672
```

>**ref|XP_001606517.1|** UGM PREDICTED: similar to CG2206-PA [Nasonia vitripennis]
Length=662

GENE ID: 100122910 LOC100122910 | similar to CG2206-PA [Nasonia vitripennis] (10 or fewer PubMed links)

Score = 65.9 bits (159), Expect = 9e-09
Identities = 29/128 (23%), Positives = 48/128 (38%), Gaps = 16/128 (12%)

```
Query  209  NEVITGMRLTKVGNAIWPEIQVAKVTLFGLIDKSTRKWVGPG-----GKMKSLCAPIKSL  263
            N V+TG R  K         +IQ  K+    G I+ ST +W+
Sbjct  444  NYVVTGARFVKKNRVVHIQIQEGKLLPNGMINSSTTRWAPINENPTTLDYHTLAWENGRE  503

Query  264  VAAEPTRLPAGHVLTQVQLVGTTKKN------VKVGGQKLNFYTGKLDKPKT-----APA  312
            V      P     VLT +            N             +K+       +++  TG+L                   PA
Sbjct  504  VNLDDLHAPKKSVLTGLAFSTESTSNFQRRLHLKIFSAPVSYETGRLWINDQKKEVNEPA  563

Query  313  KKVVKLND  320
            K  + L D
Sbjct  564  KDEITLKD  571
```

>**ref|XP_001992028.1|** G GH24541 [Drosophila grimshawi]
gb|EDV91735.1| G GH24541 [Drosophila grimshawi]
Length=827

GENE ID: 6566309 Dgri\GH24541 | GH24541 gene product from transcript GH24541-RA [Drosophila grimshawi] (10 or fewer PubMed links)

Sort alignments for this subject
E value **Score** **Percent identi**
Query start position **Subject**

Score = 65.5 bits (158), Expect = 1e-08
Identities = 30/133 (23%), Positives = 53/133 (40%), Gaps = 17/133 (12%)

```
Query  209  NEVITGMRLTKVGNAIWPEIQVAKVTLFGLIDKSTRKW------------VGPGGKMKSL  256
            N+V+TG+R  K         +IQ  ++    G I+ ST  W                   V  G       ++
Sbjct  599  NKVVTGLRFVKRNRIFHLQIQEGQLLPRGSINASTLAWKPVDDYKTFDRNVAKGRDYHTM  658

Query  257  CAPIKSLVAAEPTRLPAGH--VLTQVQL-VGTTKKNVKVGGQKLNFYTGKLDKPKTAPAK  314
              +S  +   +             V+T V+  V    T  N++        + +F  G+L +P
Sbjct  659  SYESRS-IDLDDIMKMDDNSFVVTGVRFRVLGAHLNLEARLSEFDFKRGELVQP---EVN  715

Query  315  VVKLNDLVSVDIE  327
            V + ND    V  E
Sbjct  716  VWQSNDNTDVSGE  728
```

Score = 46.2 bits (108), Expect = 0.007
Identities = 28/168 (17%), Positives = 59/168 (36%), Gaps = 21/168 (12%)

```
Query  180  DGEVVRSCKQNNVAVGASCMYAGDLKA--GANEVITGMRLTKVGNAIWPEIQVAKVTL-F  236
            D  V +          + ++    +       D+      + V+TG+R     +G    +    E             +
Sbjct  646  DRNVAKGRDYHTMSYESRSIDLDDIMKMDDNSFVVTGVRFRVLGAHLNLEARLSEFDFKR  705

Query  237  GLI-DKSTRKWVGPGGKMKSLCAPIKSLVAAEPTRLPAGHVLTQVQLVGTTKKNVKVGG  294
            G + +        W              S         K    +                +P       V+T  ++
Sbjct  706  GELVQPEVNVWQSNDNTDVSGERRQK--LKIHNVDVPTRTVVS--SLVASKHN-------  755

Query  295  QKLNFYTGKLDKPKTAPAKKVVKLNDLVSVDIEPVYVSTNVAGAGLRY  342
            + +F         +DK                   + + D+VS       P            +AG G+ Y
Sbjct  756  EYIEFVNSGMDKDAAQSTVPYLDIQDVVSSPPVP------LAGIGIYY  797
```

>**gb|ADD20695.1|** hypothetical conserved secreted protein [Glossina morsitans morsitans]
Length=635

Sort alignments for this subject
E value **Score** **Percent identi**
Query start position **Subject**

Score = 65.1 bits (157), Expect = 1e-08
Identities = 27/113 (24%), Positives = 48/113 (43%), Gaps = 13/113 (11%)

```
Query  208  ANEVITGMRLTKVGNAIWPEIQVAKVTLFGLIDKSTRKWVGP-----------GGKMKS  255
            AN+V+TG+R TK             +IQ  ++    G+I++++  W                          +
Sbjct  408  ANKVVTGLRFTKQNRVFHLQIQEGELLPRGVINRTSLAWKPVENYTLYEKGVHNNRDYYT  467

Query  256  LCAPIKSLVAAEPTRLPAGHVLTQVQLVGTTKKNVKVGGQKLNFYTGKLDKP  307
            L    +S+  +                   V+T V+  V          N++        + NF TGKL +P
Sbjct  468  LSYGARSIDLDDIVTDDNTFEVVTGVRFRVVGAHLNLEARLTEFNFETGKLVRP  520
```

Score = 41.2 bits (95), Expect = 0.22
Identities = 13/64 (21%), Positives = 26/64 (41%), Gaps = 1/64 (1%)

```
Query  174  ENRFGNDGEVVRSCKQNNVAVGASCMYAGDLKAGANE-VITGMRLTKVGNAIWPEIQVAK  232
            EN    +   V         +     ++  GA    + D+     N V+TG+R    VG    +    E  ++ +
Sbjct  450  ENYTLYEKGVHNNRDYYTLSYGARSIDLDDIVTDDNTFEVVTGVRFRVVGAHLNLEARLTE  509

Query  233  VTLF  236

Sbjct  510  FNFE  513
```

>**gb|EFA12874.1|** hypothetical protein TcasGA2_TC010726 [Tribolium castaneum]
Length=304

Sort alignments for this subject
E value **Score** **Percent identi**
Query start position **Subject**

Score = 64.3 bits (155), Expect = 2e-08
Identities = 33/147 (23%), Positives = 60/147 (41%), Gaps = 23/147 (15%)

```
Query  183  VVRSCKQNNVAVGA------SCMYAGDLKAGANEV---ITGMRLTKVGNAIWPEIQVAKV  233
            ++ S     NN   + +               S +         N V         +TG+R  K     +    +IQ  K+
Sbjct  7    IIESDDINNRDLSSIPPFCTSSVTNNNINWCPNHVFTVVTGLRFIKHNRIVHLQVQEGKL  66

Query  234  TLFGLIDKSTRKWVGP-----------GGKMKSLCAPIKSLVAAEPTRLPAGHVLTQVQ  281
                G I+ +T +WV P                    G    ++       ++ +             AG V+T V+
Sbjct  67   LEKGNINPTTVQWVPPEDYKITDRDIYKGQDYHTISWEERT-IELTKVIAKAGSVVTGVR  125

Query  282  LVGT-TKKNVKVGGQKLNFYTGKLDKP  307
              T       N++V         +F TGKL+K
Sbjct  126  FKRTFGSLNLQVLTTAFDFNTGKLEKN  152
```

Score = 36.2 bits (82), Expect = 7.2
Identities = 13/98 (14%), Positives = 34/98 (35%), Gaps = 3/98 (3%)

```
Query  179  NDGEVVRSCKQNNVAVGASCMYAGDLKAGANEVITGMRLTKVGNAIWPEIQVAKVTLF--  236
             D ++ +          + ++       +      A A    V+TG+R     +    ++
Sbjct  88   TDRDIYKGQDYHTISWEERTIELTKVIAKAGSVVTGVRFKRTFGSLNLQVLTTAFDFNTG  147

Query  237  GLIDKSTRKW-VGPGGKMKSLCAPIKSLVAAEPTRLPA  273
             L    S     W +          ++       +     V      P+ +P
Sbjct  148  KLEKNSDSDWGIFESAPYFNIRHKFRLKVNLNPSDVPV  185
```

>**gb|EFA08551.1|** hypothetical protein TcasGA2_TC006206 [Tribolium castaneum]
Length=447

Sort alignments for this subject
E value **Score** **Percent identi**
Query start position **Subject**

Score = 64.0 bits (154), Expect = 3e-08
Identities = 28/109 (26%), Positives = 44/109 (41%), Gaps = 14/109 (12%)

```
Query  209  NEVITGMRLTKVGNAIWPEIQVAKVTLFGLIDKSTRKWVGPGGKMKSLCAPI--------  260
            N+V+TG+R  K      I  ++Q  K+    G ID  +WV P         K    I
Sbjct  228  NKVVTGLRFVKHNQIIHLQVQEGKLLEHGTIDPDSVQWVPP-ENYKRTDRKIHEGQDYQQV  286

Query  261  ----KSLVAAEPTRLPAGHVLTQVQLVG-TTKKNVKVGGQKLNFYTGKL  304
                  K+             G V+T V+         ++       +++F GKL
Sbjct  287  ITWEKRSIELTEIMARNGSVVTGVRFKKIGSRLELEIMITPFNFTTGKL  335
```

Score = 37.0 bits (84), Expect = 4.2
Identities = 12/55 (22%), Positives = 23/55 (42%), Gaps = 0/55 (0%)

```
Query  174  ENRFGNDGEVVRSCKQNNVAVGASCMYAGDLKAGANEVITGMRLTKVGNAIWPEI  228
            EN      D ++         +    ++       A    V+TG+R  K+G+   EI
Sbjct  269  ENYKRTDRKIHEGQDYQQVITWEKRSIELTEIMARNGSVVTGVRFKKIGSRLELEI  323
```

>**ref|XP_001960321.1|** G GF11569 [Drosophila ananassae]
gb|EDV37143.1| G GF11569 [Drosophila ananassae]
Length=662

GENE ID: 6494433 Dana\GF11569 | GF11569 gene product from transcript GF11569-RA [Drosophila ananassae] (10 or fewer PubMed links)

Score = 63.6 bits (153), Expect = 4e-08
Identities = 28/114 (25%), Positives = 48/114 (43%), Gaps = 13/114 (11%)

```
Query  209  NEVITGMRLTKVGNAIWPEIQVAKVTLFGLIDKSTRKWVGP-----------GGKMKSL  256
            N ++TG+R  K         +Q A++    G I+ ST +W+                          +L
Sbjct  438  NRIVTGVRFVKRSRVFHLQLQQAQLVPWGAINTSTLEWKPLLDDYNVSDADIRNDYDYHTL  497

Query  257  CAPIKSLVAAEPTRLPAGHVLTQVQLVGTTKKNVKVGGQKLNFYTGKLDKPKT  309
            +SL                   V+T V+  V          N++        + NF TG L +P+T
Sbjct  498  GPDSRSLDLDEIVANATDQVVTGVRFRIINKHLNLEVRFTSFNFTSGTLLVEPQT  551
```

>**ref|XP_001603438.1|** UGM PREDICTED: similar to GA15301-PA [Nasonia vitripennis]
Length=669

GENE ID: 100119712 LOC100119712 | venom protein E [Nasonia vitripennis] (10 or fewer PubMed links)

Score = 63.2 bits (152), Expect = 6e-08
Identities = 27/163 (17%), Positives = 67/163 (42%), Gaps = 17/163 (10%)

```
Query  187  CKQNNVAVGASCMYAGDLKAGANE--VITGMRLTKVGNAIWPEIQVAKVTLFGLIDKSTR  244
            C  +N  +           ++ AN+  V+TG+R  K  N I   +I+    +  G +++ST
Sbjct  430  CDDSNTEID-RYFSLRNVTSDANKNYVVTGIRFAKKNNIIHIQIQEGKLLKNGNVNETS  488

Query  245  KWVG------------PGGKMKSLCAPIKSLVAAEPTRLPAGHVLTQVQLVG-TTKKNVK  291
              W                          ++  +S V           +P    +V+T  ++           ++
Sbjct  489  HWKAVDNFTIYDKMRKLIDRDYHNITWSQRS-VDLDTVDSPLNNVITGLRFQNVDGHLKLE  547

Query  292  VGGQKLNFYTGKLDKPKTAPAKKVVKLNDLVSVDIEPVYVSTN  334
                +++F+Y G+L     +     ++   ++  +    T +
Sbjct  548  IQVTPIDFYRGELKIDESTWISNSKSVHGRIELVNENLDLGTS  590
```

>**gb|EFA08552.1|** hypothetical protein TcasGA2_TC006207 [Tribolium castaneum]
Length=607

Sort alignments for this subject
E value **Score** **Percent identi**
Query start position **Subject**

Score = 62.4 bits (150), Expect = 9e-08
Identities = 30/121 (25%), Positives = 43/121 (36%), Gaps = 11/121 (9%)

```
Query  209  NEVITGMRLTKVGNAIWPEIQVAKVTLFGLIDKSTRKWVGPGGKMKSLCAPIKSL-----  263
            N V+TG+R  K         +IQ  K+    G ID    +WV
Sbjct  377  NRVVTGLRFVKHNEIFHLQIQEGKLLKRGKIDPSDVRWVPVDNYKHFDTIEGKDFHKITW  436

Query  264  ----VAAEPTRLPAGHVLTQVQLVGTTKKNVKVGGQKLNFYTGKLDKPKTAPAKKVVKL  318
                                 G V+T V+         +++V         NF TGKL      P    K     L
Sbjct  437  EKRSFELTSIMAQNGSVVTGVRFRLIRNSLDLEVLTTPFNFTTGKLL-PSLSTFKGTRNL  495

Query  319  N  319
            N
Sbjct  496  N  496
```

Score = 39.3 bits (90), Expect = 0.85
Identities = 14/95 (15%), Positives = 29/95 (31%), Gaps = 5/95 (5%)

```
Query  182  EVVRSCKQNNVAVGASCMYAGDLKAGANEVITGMRLTKVGNAIWPEIQVAKVTLFGLIDK  241
              + +           + +                + A     V+TG+R   + N+   E+           T F
Sbjct  424  DTIEGKDFHKITWEKRSFELTSIMAQNGSVVTGVRFRLIRNSLDLEVLT---TPFNFTTG  480

Query  242  STRKWVGPGGKMKSLCA--PIKSLVAAEPTRLPAG  274
                              ++L          K      V              +P G
Sbjct  481  KLLPSLSTFKGTRNLNRHKFKQQVKLSKPDVPTG  515
```

>**ref|XP_001360382.2|** G GA15824 [Drosophila pseudoobscura pseudoobscura]
gb|EAL24957.2| GA15824 [Drosophila pseudoobscura pseudoobscura]
Length=670

GENE ID: 4803708 Dpse\GA15824 | GA15824 gene product from transcript GA15824-RA [Drosophila pseudoobscura pseudoobscura] (10 or fewer PubMed links)

Score = 62.4 bits (150), Expect = 9e-08
Identities = 23/116 (20%), Positives = 47/116 (41%), Gaps = 16/116 (13%)

```
Query  209  NEVITGMRLTKVGNAIWPEIQVAKVTLFGLIDKSTRKWVGPG------------KMKSL  256
            N V+TG+R  K         ++Q  ++    G I+ S+ +W+                                 +L
Sbjct  435  NKVVTGVRFVKSNRVFHLQLQQGELLPRGAINVSSLEWLPLDSYHLNDVDVRNDYDYHTL  494

Query  257  CAPIKSLVAAEPTRLPAGH--VLTQVQLVGTTKKNVKVGGQKLNFYTGKLDKPKT  309
                +++             +          V+T V    V     N++        + NF TG+L +PK
Sbjct  495  TVDSRAM-DLDEITSGSNRSLVVTGARFRVWHKHLNLEVRFSYFDFPTGRLVEPKR  549
```

>**ref|XP_002015853.1|** G GL10792 [Drosophila persimilis]
gb|EDW31743.1| G GL10792 [Drosophila persimilis]
Length=670

GENE ID: 6590462 Dper\GL10792 | GL10792 gene product from transcript GL10792-RA [Drosophila persimilis] (10 or fewer PubMed links)

Score = 62.4 bits (150), Expect = 9e-08
Identities = 23/116 (20%), Positives = 47/116 (41%), Gaps = 16/116 (13%)

```
Query  209  NEVITGMRLTKVGNAIWPEIQVAKVTLFGLIDKSTRKWVGPG------------KMKSL  256
            N V+TG+R  K         ++Q  ++    G I+ S+ +W+                                 +L
Sbjct  435  NKVVTGVRFVKSNRVFHLQLQQGELLPRGAINVSSLEWLPLDSYHLNDVDVRNDYDYHTL  494

Query  257  CAPIKSLVAAEPTRLPAGH--VLTQVQLVGTTKKNVKVGGQKLNFYTGKLDKPKT  309
                +++             +          V+T V    V     N++        + NF TG+L +PK
Sbjct  495  TVDSRAM-DLDEITSGSNRSLVVTGARFRVWHKHLNLEVRFSYFDFPTGRLVEPKR  549
```

>**ref|XP_969565.1|** UGM PREDICTED: similar to lethal (1) G0193 CG2206-PA [Tribolium cast
Length=396

GENE ID: 658059 LOC658059 | similar to CG2206-PA, isoform A [Tribolium castaneum]

Sort alignments for this subject
E value **Score** **Percent identi**
Query start position **Subject**

Score = 62.4 bits (150), Expect = 9e-08
Identities = 30/121 (25%), Positives = 43/121 (36%), Gaps = 11/121 (9%)

```
Query  209  NEVITGMRLTKVGNAIWPEIQVAKVTLFGLIDKSTRKWVGPGGKMKSLCAPIKSL-----  263
            N V+TG+R  K         +IQ  K+    G ID    +WV
Sbjct  166  NRVVTGLRFVKHNEIFHLQIQEGKLLKRGKIDPSDVRWVPVDNYKHFDTIEGKDFHKITW  225

Query  264  ----VAAEPTRLPAGHVLTQVQLVGTTKKNVKVGGQKLNFYTGKLDKPKTAPAKKVVKL  318
                                 G V+T V+         +++V         NF TGKL      P    K     L
Sbjct  226  EKRSFELTSIMAQNGSVVTGVRFRLIRNSLDLEVLTTPFNFTTGKLL-PSLSTFKGTRNL  284

Query  319  N  319
            N
Sbjct  285  N  285
```

Score = 39.3 bits (90), Expect = 0.85
Identities = 14/95 (15%), Positives = 29/95 (31%), Gaps = 5/95 (5%)

```
Query  182  EVVRSCKQNNVAVGASCMYAGDLKAGANEVITGMRLTKVGNAIWPEIQVAKVTLFGLIDK  241
              + +           + +                + A     V+TG+R   + N+   E+           T F
Sbjct  213  DTIEGKDFHKITWEKRSFELTSIMAQNGSVVTGVRFRLIRNSLDLEVLT---TPFNFTTG  269

Query  242  STRKWVGPGGKMKSLCA--PIKSLVAAEPTRLPAG  274
                              ++L          K      V              +P G
Sbjct  270  KLLPSLSTFKGTRNLNRHKFKQQVKLSKPDVPTG  304
```

>**ref|XP_001603409.1|** UGM PREDICTED: similar to CG2206-PA [Nasonia vitripennis]
Length=675

GENE ID: 100119678 LOC100119678 | similar to CG2206-PA [Nasonia vitripennis] (10 or fewer PubMed links)

Sort alignments for this subject
E value **Score** **Percent identi**
Query start position **Subject**

Score = 61.6 bits (148), Expect = 2e-07
Identities = 30/141 (22%), Positives = 53/141 (38%), Gaps = 14/141 (9%)

```
Query  207  GANEVITGMRLTKVGNAIWPEIQVAKVTLFGLIDKSTRKWVG------------PGGKM  254
              N V+TG+R  K         +IQ  K+    G I   S T  W
Sbjct  452  DKNMVVTGVRFAKENHIFHIQIQQGKLVENGNIKASTVEWKPVENFTIHDDSVVLNRDYH  511

Query  255  SLCAPIKSLVAAEPTRLPAGHVLTQVQLVGT-TKKNVKVGGQKLNFYTGKLDKPKTAPAK  313
            ++   + LV           P    HVLT ++                 NFYT +L   +
Sbjct  512  NITWANR-LVYLDTFDSPLHHVLTGLRFQLQNGDLKLEIQVTPFNFYTAELKTNESTWMS  570

Query  314  KVVKLNDLVSVDIEPVYVSTN  334
                       +D+E + TN
Sbjct  571  NSIGAYGRGELDLENLDLGTN  591
```

Score = 36.2 bits (82), Expect = 7.2
Identities = 15/63 (23%), Positives = 26/63 (42%), Gaps = 0/63 (0%)

```
Query  174  ENRFGNDGEVVRSCKQNNVAVGASCMYAGDLKAGANEVITGMRLTKVGNAIWPEIQVAKV  233
            EN      +D  VV +      +N+            +Y             +  V+TG+R       EIQV
Sbjct  495  ENFTIHDDSVVLNRDYHNITWANRLVYLDTFDSPLHHVLTGLRFQLQNGDLKLEIQVTPF  554

Query  234  TLF  236
               +
Sbjct  555  NFY  557
```

>**ref|XP_001602724.1|** GM PREDICTED: similar to CG2206-PA [Nasonia vitripennis]
Length=894

GENE ID: 100118844 LOC100118844 | venom protein W [Nasonia vitripennis] (10 or fewer PubMed links)

Score = 59.7 bits (143), Expect = 6e-07
Identities = 34/134 (26%), Positives = 53/134 (40%), Gaps = 23/134 (17%)

```
Query  182  EVVRSCKQNNVAVGASCMYAGDLKAGA-----------------NEVITGMRLTKVGNAI  224
            E+ R     +N+A   SCM    D   A                          N V+TG+R    K   +
Sbjct  628  ELDRHYSANNMACDSCMCLDDDAHPKTERYFSLISSIAAVKENYVLTGARLVKRETH  687

Query  225  WPEIQVAKVTLFGLIDKSTRKWVGPGGK--MKSLCAPIKSL-----VAAEPTRLPAGHVLT  277
               +IQ    ++   +G +++ST  W          +       K    L            V    +P  +V
Sbjct  688  HVQIQQGQLLPYGHVNESTVEWVPIKADVLAKDEVYKIKWLGSRTVFLDDIEVPRNNVV  747

Query  279  QVQLVGTTKKNVKV  292
                 +        N+KV
Sbjct  748  GLGFKAPEQNNIKV  761
```

>**ref|XP_002049066.1|** G GJ21381 [Drosophila virilis]
gb|EDW60259.1| G GJ21381 [Drosophila virilis]
Length=647

GENE ID: 6625370 Dvir\GJ21381 | GJ21381 gene product from transcript GJ21381-RA [Drosophila virilis] (10 or fewer PubMed links)

Score = 59.3 bits (142), Expect = 8e-07
Identities = 28/112 (25%), Positives = 44/112 (40%), Gaps = 13/112 (11%)

```
Query  209  NEVITGMRLTKVGNAIWPEIQVAKVTLFGLIDKSTRKWVGP-----------GGKMKSL  256
            N+++TG R  K         +Q  ++    GLI++S+  W+                          G        L
Sbjct  426  NKIVTGARFIKDQRIFHLQLQQGELLPGGLINGSSLDWIPLESFDVNHVDIRNGFDYHEL  485

Query  257  CAPIKSLVAAEPTRLPAGHVLTQVQLVGTTKKNVKVGGQKLNFYTGKLDKP  307
                +SL               E   T           V+T  V       N++V     +  NF TG+L  P
Sbjct  486  TFESRSLGLDEITAKNDSLVVTGARFRVIDKHLNLEVRFASLNFTTGRLNP  537
```

>**ref|XP_002062683.1|** G GK19560 [Drosophila willistoni]
gb|EDW73669.1| G GK19560 [Drosophila willistoni]
Length=672

GENE ID: 6639766 Dwil\GK19560 | GK19560 gene product from transcript GK19560-RA [Drosophila willistoni] (10 or fewer PubMed links)

Score = 58.2 bits (139), Expect = 2e-06
Identities = 26/117 (22%), Positives = 46/117 (40%), Gaps = 17/117 (14%)

```
Query  208  ANEVITGMRLTKVGNAIWPEIQVAKVTLFGLIDKSTRKWVGP------------GGKMKS  255
             V TG+R  K         +Q  ++    G I+ S+ +W+                                   G
Sbjct  443  NGRVATGIRFVKSQRVFHLQLQQGELLPNGAINGSSLDWLPIDSYDPDNVDVRNGYDYHD  502

Query  256  LCAPIKSLVAAEPT---RLPAGHVLTQVQLVGTTKKNVKVGGQKLNFYTGKLDKP  308
            L     +S+  L         +  HV+T V+ L    +  N++V         +  NF TG+L  K
Sbjct  503  LSFDSRTL-DLDEVSESHSSDHVVTGVRLRMLGKHLNLEVRFSEYNFTTGRLLDAK  558
```

>**ref|XP_001600223.1|** GM PREDICTED: similar to GA15301-PA [Nasonia vitripennis]
Length=497

GENE ID: 100115520 LOC100115520 | similar to GA15301-PA [Nasonia vitripennis] (10 or fewer PubMed links)

Score = 57.4 bits (137), Expect = 3e-06
Identities = 53/256 (21%), Positives = 91/256 (35%), Gaps = 25/256 (9%)

```
Query  67   YNTTNNHVGMEEGLAKKLQS-EPTTCDAARTISYTIRERSDYF--GNQLANNRPDDRILC  123
            Y  T  N       +E     +E   + CD  R          +  + DY  F       N+L          +C
Sbjct  141  YATVTNCANKQEYYDTEVQYYEYSICDQQRKCNGALFDCEDYFPDYNELW--------IC  192

Query  124  PVTIPKMQREHELVMGFLLNRLRIFALRAKKCTDNWESKDRLARGLNRVAENRFGNDGEV  183
            P T+    +R            G +             +R           +    DR           R           F
Sbjct  193  PSTLNSSRRYEFMEHGSVDPVIFKHDVRQYGKGDKKKCDRHITYDYREFGQSFKDAWRY  252

Query  184  VRSCKQNNVAVGASC----MYAGDLKAGANEVITGMRLTKVGNAIWPEIQVAKVTLFGLI  239
                  C                      +              +   A    V+TG  RL  K  +         +I+           +  G I
Sbjct  253  CEVCLCTCHDFKSPTSEQQINVRPVVADNDYVVTGARLVKKNHILHIHVQIEQGKLLADGRI  312

Query  240  DKSTRKW---VGPGGKM--KSLCAPIKSLVAAEPTRLPAGHVLTQVQLV-GTTKKNVKVG  293
            D ST W           P           +S                         +  E   T  +P  HVL  V+L+    +  N++
Sbjct  313  DNSTLHWKTIAEPRNVYDLRSFSWNGFRTIYLEVEVPKNHVLVGLKLTNLGNGDGQMMS  372

Query  294  GQKL----NFYTGKLD  305
              + L         ++  TG+L
Sbjct  373  FKTLSVPYDYSTGELT  388
```

>**gb|ACZ28336.1|** hypothetical conserved protein [Simulium nigrimanum]
Length=408

Score = 57.0 bits (136), Expect = 4e-06
Identities = 27/139 (20%), Positives = 51/139 (37%), Gaps = 19/139 (13%)

```
Query  207  GANEVITGMRLTKVGNAIWPEIQVAKVTLFGLIDKSTRKW--------VGPGGKMKSLCA  258
                  NEVI G++  K           ++ +    A   +   F    +D                       W        V       P
Sbjct  184  DENEVIVGVKFVKHNRVLYLQTMKAPLLPFASVDTDQAVWNELDELGLVPP--DYHNFSG  241

Query  259  PI----KSLVAAEPTRLPAGHVLTQVQLVGTTKKNVKVGGQKLNFYTGKLDKPKT----  309
                    +          +PAG VLT V+           ++           +++ TGL+   +
Sbjct  242  LYTFDHKHREIMKELEVPAGTVVTGVKFIVKDGVPDLSIRYTPVDWTTTGELNPAASLWI  301

Query  310  APAKKVVKLNDLVSVDIEP  328
                        A           ND++    P
Sbjct  302  TEAHDAYTDNDMLYQNRLP  320
```

>**ref|XP_001606530.1|** GM PREDICTED: similar to CG2206-PA [Nasonia vitripennis]
Length=880

GENE ID: 100122920 LOC100122920 | similar to CG2206-PA [Nasonia vitripennis] (10 or fewer PubMed links)

Score = 57.0 bits (136), Expect = 4e-06
Identities = 29/137 (21%), Positives = 52/137 (37%), Gaps = 12/137 (8%)

```
Query  209  NEVITGMRLTKVGNAIWPEIQVAKVTLFGLIDKSTRKWV---GPGGKMKSLCAPIKSLVA  265
            N V+TG RL K         I  +IQ  ++    G I+ ST +W                         +L       K
Sbjct  443  NYVLTGARLVKRNRVMHIQIQEGQLLQNGRINASTLRWSKINENPISYHTLQWEQKREIH  502

Query  266  AEPTRLPAGHVLTQVQLVG-------TTKKNVKVGGQKLNFYTGKLDKPKTAP-AKKV  316
            +    P    HVLT +                 T    +++          L F+G+G+            P         K
Sbjct  503  LDDLMAPKNHVLTGLSFTTIQVDSAYTKRLKLQAYAAPLYFHSGEVIDKHQMPLYPKDFS  562

Query  317  KLNDLVSVDIEPVYVST  333
               N       + ++ + V T
Sbjct  563  PTNATNEIKLDNLDVPT  579
```

>**ref|XP_001603377.1|** GM PREDICTED: similar to CG2206-PA [Nasonia vitripennis]
Length=671

GENE ID: 100119643 LOC100119643 | similar to CG2206-PA [Nasonia vitripennis] (10 or fewer PubMed links)

Sort alignments for this subject
E value **Score** **Percent identi**
Query start position **Subject**

Score = 57.0 bits (136), Expect = 4e-06
Identities = 25/108 (24%), Positives = 41/108 (38%), Gaps = 12/108 (11%)

```
Query  211  VITGMRLTKVGNAIWPEIQVAKVTLFGLIDKSTRKWVGPGG--------KMKSLCAP---  259
            V+TG+R  K  N      +IQ  K+ + G IDKS+ W
Sbjct  457  VVTGVRFAKKNNIIHIQIQRGKLLVNGNIDKSSVDWKPVDNFTVSDKHFVDRHYDYKISW  516

Query  260  IKSLVAAEPTRLPAGHVLTQVQLVGTTKKNVKVGGQKLNFYTGKLDK  306
                +           +    P    +VL  ++            +        N+    +       +G+L    K
Sbjct  517  NQRTVYLDTLDAPLNNVLIGLRFQVDNGSLNLKFEIQMTPFNFSSGELK  564
```

Score = 37.0 bits (84), Expect = 4.2
Identities = 17/85 (20%), Positives = 32/85 (38%), Gaps = 2/85 (2%)

```
Query  174  ENRFGNDGEVVRSCKQNNVAVGASCMYAGDLKAGANEVITGMRLTKVGNAIWPEIQVAKV  233
            +N      +D        V           ++          +Y        L     A     N  V+G+R           G+  EIQ+
Sbjct  496  DNFTVSDKHFVDRHYDYKISWNQRTVYLDTLDAPLNNVLIGLRFQVDNGSLNLKFEIQMTPF  555

Query  234  TLFG--LIDKSTRKWVGPGGKMKSL  256
                     L     K+     +W+
Sbjct  556  NFSSGELKKKNETRWISNSSSSERTE  580
```

>**gb|ACH56860.1|** hypothetical protein [Simulium vittatum]
Length=351

Score = 56.3 bits (134), Expect = 7e-06
Identities = 22/138 (16%), Positives = 54/138 (39%), Gaps = 17/138 (12%)

```
Query  207  GANEVITGMRLTKVGNAIWPEIQVAKVTLFGLIDKSTRKW--------VGPG---GKMK  254
            N++I G+R  K           ++  I   A    +    +D    +  W                     G           K++
Sbjct  127  DENQIIVGVRFVKRNRVLYLQIMQAPLLPYAAVDMDSAEWRDDIGELGMLPPEHTNLSGYH  186

Query  255  SLCAPIKSLVAAEPTRLPAGHVLTQVQLVGTTKKNVKVGGQKLNFYTGKLDKPKT---A  310
                    +           +        +P G +LT V+    +          +P         +        +   TG+LD
Sbjct  187  AFDHKNREMY-MKELEVPGGSLLTGVKFIIKDGVPDLSIRYTTFVNWTTGELNQSSSEWMT  245

Query  311  PAKKVVKLNDLVSVDIEP  328
                         ND++    P
Sbjct  246  EKHDTYDTNDMLYQNQLP  263
```

>**emb|CBM69271.1|** venom protein Ci-48a [Chelonus inanitus]
Length=383

Score = 53.6 bits (127), Expect = 4e-05
Identities = 19/104 (19%), Positives = 37/104 (36%), Gaps = 13/104 (12%)

```
Query  187  CKQNNVAVGASCMYAGDLKAGANE--VITGMRLTKVGNAIWPEIQVAKVTLFGLIDKSTR  244
            C   +          + +        + +                 +V+TG R           +  +IQ   K+      LID+ T
Sbjct  152  CDNSEKPSATAAVCLDKISSDVERGYVVTGARFRIKNRVLRMQIQQGKLIDNQLIDQDTL  211

Query  245  KWVGPGGKMKSLCAPIKSL------VAAEPTRLPAGHVLTQVQL  282
            +W    G                C    K+                   +    +    LP  +  T  V+
Sbjct  212  EWQELG-----DCKSKKNMSYDYRALQTDDITLPNEYFVTGLSF  250
```

>**ref|XP_001601835.1|** UGM PREDICTED: similar to ef hand protein [Nasonia vitripennis]
Length=796

GENE ID: 100117668 LOC100117668 | similar to ef hand protein [Nasonia vitripennis] (10 or fewer PubMed links)

Score = 52.0 bits (123), Expect = 1e-04
Identities = 24/117 (21%), Positives = 43/117 (37%), Gaps = 11/117 (9%)

```
Query  198  CMYAGDLKAGANEVITGMRLTKVGNAIWPEIQVAKVTLFGLIDKSTRKWVGPGGKMKSL  257
            C+              +  N+VITG RL K GN  ++  +IQ      G    I   +W                     S
Sbjct  279  CLRNVVSDSDNNKVITGARLVKKGNVLYIQIQQGTLGANGSI-VDVPQWQPVKEESPRNL  337

Query  258  APIKSL---------VAAEPTRLPAGHVLTQVQLVGTTKKNVKVGGQKLNFYTGKL  304
                K++                             V    +    LT ++                                   N+   TG L
Sbjct  338  HNNKNVYQVNYDQNTVFLDVLKSQPAYALTGLRFQKAENSLRLEAQITPFNYETGEL  394
```

>**gb|ADJ67267.1|** Cul n 3 allergen [Culicoides nubeculosus]
Length=390

Score = 50.1 bits (118), Expect = 5e-04
Identities = 21/133 (16%), Positives = 48/133 (37%), Gaps = 11/133 (8%)

```
Query  187  CKQNNVAVGASCMYAGDLKAGA--NEVITGMRLTKVGNAIWPEIQVAKVTLFGLIDKSTR  244
            C    +           +          LK+          N+V+TG++++   +     +  +I+    +   +    G   K+
Sbjct  166  CDEPKDSQSVRSFSLNSLKSDTRENKVVTGVQIVKNNVVFYLKIHQGRLLPYGKVKKTGG  225

Query  245  KWVGPGGKMKSLCAPIKS-------LVAAEPTRLPAGHVLTQVQLVGTTKKNVKVGGQK  296
                 W                                                 +P         +V+T V+                     +
Sbjct  226  SWKTDNPSNNTFNLAFNKHNIHMGTSTVVKPKK-KLEYVVTGVKFADIEGNLALGVKFTQ  284

Query  297  LNFYTGKLDKPKT  309
                +   TGKL
Sbjct  285  FDIKTGKLLPDQK  297
```

>**gb|ADJ67269.1|** Cul n 5 allergen [Culicoides nubeculosus]
Length=405

Score = 47.8 bits (112), Expect = 0.002
Identities = 25/126 (20%), Positives = 48/126 (39%), Gaps = 10/126 (7%)

```
Query  193  AVGASCMYAGDLKAGANEVITGMRLTKVGNAIWPEIQVAKVTLFGLIDKSTRKWV--GPG  250
            +V  +           G           ++V+TG+++                                    +           ++
Sbjct  177  SVRSFSLNTVTADKGEHKVVTGIQIGMKDKIFFLKIQQSPLLPFGRINSASGSKKADDPS  236

Query  251  GKMKSL------CAPIKSLVAAEPTRLPAGHVLTQVQLVGTTKKNVKVGGQKLNFYTGK  303
            G+K                                                        L              A           +L          +V+T V+                     +           V          K         + TGK
Sbjct  237  SGTKLFKLQYNKRGIHMGAAEANVKLSE-YVVTAVKFVEHKGNMALGVELTQFDISTGK  295

Query  304  LDKPKT  309
            L  +
Sbjct  296  LLENSK  301
```

>**gb|ABY19389.1|** venom protein 2 [Microctonus hyperodae]
Length=679

Score = 47.0 bits (110), Expect = 0.004
Identities = 26/139 (19%), Positives = 44/139 (32%), Gaps = 40/139 (28%)

```
Query  208  ANEVITGMRLTKVGNAIWPEIQVAKVTLFGLIDKSTRKWVGPGGKMKSLCAPIKS----  263
            AN VITG++          I     I+   +     G   I                T   W+        P
Sbjct  433  ANMVITGVKFISSDKIHIQIQEGKLMSGGRIKPETEHWIEPENFEYDPKGILKSRKVGS  492

Query  264  --------------------------VAAEPTRLPAGHVLTQVQLVGTTKK--------  288
                                                          +     +     P+G+V+T V+              +
Sbjct  493  FFLSTGNKMTLMKHGMDFTFVTFFQREINLDDVKAPSGYVVTGVKFVREGNDTDLSRRR  552

Query  289  -NVKVGGQKLNFYTGKLDK  306
                    +  K+F   G L   K
Sbjct  553  LQLAIYVTKFDFLNGVLMK  571
```

>**ref|XP_001605439.1|** GM PREDICTED: similar to ENSANGP00000010019 [Nasonia vitripennis]
Length=585

GENE ID: 100121830 LOC100121830 | similar to ENSANGP00000010019 [Nasonia vitripennis] (10 or fewer PubMed links)

Sort alignments for this subject
E value **Score** **Percent identi**
Query start position **Subject**

Score = 47.0 bits (110), Expect = 0.004
Identities = 30/167 (18%), Positives = 60/167 (36%), Gaps = 28/167 (16%)

```
Query  209  NEVITGMRLTKVGNAIWPEIQVAKVTLFGLIDKSTRKWVGP------------GGKMKSL  256
            N+V+TG+R+  +           +  ++Q  ++                 G  ++                  W             +
Sbjct  369  NKVLTGVRIVLMNRTVQFQVQQGQLLVENGNKAQRSVNWGRLQPLDPLDRSFVAGKDYHRI  428

Query  257  CAPIKSLVAAEPTRLPAGHVLTQVQLVGTTKKNVKVGGQKLNFYTGKLDKPKTAPAKK  315
                +     V              A        LT ++                         ++F       K            + +
Sbjct  429  TYDART-VNIDSLEAPADCALTGLKFEKEGSSLKLAMQYTPIDFANGKLIASGSTWISSP  487

Query  316  V----------KLNDLV--SVDIEPVYVSTNVV--AGAGLRYDVETKT  348
            V                                       L D        + +          + V                                 G+GL     Y  K
Sbjct  488  VGSSRDSSLEDLGGSVVPFIDSQPITSTPAVLLSGAGLVYKGGRKS  534
```

Score = 38.2 bits (87), Expect = 1.9
Identities = 19/112 (17%), Positives = 40/112 (36%), Gaps = 8/112 (7%)

```
Query  180  DGEVVRSCKQNNVAVGASCMYAGDLKAGANEVITGMRLTKVGNAIWPEIQVAKVTL-FGL  238
            D               V               L                 A           L+A  A+           +TG+++         K     +            +Q           +               P           +
Sbjct  416  DRSFVAGKDYHRITYDARTVNIDSLEAPADCALTGLKFEKEGSSLKLAMQYTPIDFANGK  475

Query  239  IDKSTRKWVGPGGKMKSLCAPIKSL-------VAAEPTRLPAGHVLTQVQL  283
            +    S     W+                                                            +                        L                                                                 +             ++P                                    +L+          LV
Sbjct  476  LIASGSTWISSPVGSSRDSSLEDLGGSVVPFIDSQPITSTPAVLLSGAGLV  527
```

>**gb|ACM40903.1|** secreted salivary protein [Culicoides nubeculosus]
Length=417

Score = 45.9 bits (107), Expect = 0.009
Identities = 21/128 (17%), Positives = 48/128 (38%), Gaps = 10/128 (7%)

```
Query  187  CKQN--NVAVGASCMYAGDLKAGANEVITGMRLTKVGNAIWPEIQVAKVTLFGLIDKSTR  244
            C  ++        +     ++       +    ++ +       A   +         N+V+      G++L               +     ++I   +    +      G    D+
Sbjct  187  CDESKDSESIRSFSLNAVESDTKDNKVVIGVQLVKDKIFYFKIRQSPLLPLGKADQKRKL  246

Query  245  KW-----VGPGGKMKSLCAPIKSLVAAEPTRLPAGHVLTQVQLVGTTKKNVKVGGQK  296
                  W                                                                                                  +                       L                                                    +S+                                        T                                                           +               V+T                           V+                                                                                                   +                  V                        K
Sbjct  247  SWKSDDPSLSSASKDIFKLDVNKRSIHIGSATSNLSDFVVTGVKFAEIDGNLALGVKLTK  306

Query  297  LNFYTGKL  304
                    +                       GKL
Sbjct  307  FDVMSGKL  314
```

>**gb|ACE75210.1|** conserved hypothetical protein [Glyptapanteles flavicoxis]
Length=645

Score = 45.9 bits (107), Expect = 0.009
Identities = 23/133 (18%), Positives = 44/133 (34%), Gaps = 28/133 (21%)

```
Query  209  NEVITGMRLTKVGNAIWPEIQVAKVTLFGLIDKSTRKWVGPG-----------------  250
            N V+TG+R                                       +I+    +++   FG       I                      T              W          G
Sbjct  380  NMVMTGVRFAQKDRIFHLQIEQSELGPFGEIVPGTVEWKELGDFQYDSTGEGSFSIKKGD  439

Query  251  -------GKMKSLCAPIKSLVAAEPTRLPAGHVLTQVQLVGTTKKN---VKVGGQKLNFY  300
                                                                                               S                                 A                 +                             +                                                               G                                     VL                                                V                                                          +                                                             +                                                              +V              V
Sbjct  440  EFVKLTEFVDFSFVALDQRTINLDELFANDGQVLIGVRFIYNGMDDAFELQVKSWPVDIR  499

Query  301  TGKLDKPKTAPAK  313
            TGKL                                                                                                       +                                                    +
Sbjct  500  TGKLADGNPSDVE  512
```

>**ref|XP_001605051.1|** UGM PREDICTED: similar to GA15301-PA [Nasonia vitripennis]
Length=592

GENE ID: 100121438 LOC100121438 | similar to GA15301-PA [Nasonia vitripennis] (10 or fewer PubMed links)

Score = 45.5 bits (106), Expect = 0.012
Identities = 25/121 (21%), Positives = 43/121 (36%), Gaps = 23/121 (19%)

```
Query  209  NEVITGMRLTKVGNAIWPEIQVAKVTLFGLIDKSTRK------WVGP-----------G  250
            N V+TGM+                              I     I+   +K+   GLI+   +                                   W
Sbjct  381  NYVVTGMKFELKNGIIHIRIKQSKLLANGLIENAVSELPKSEQWKKIDDYTINDKDIFEG  440

Query  251  GKMKSLCAPIKSLVAAEPTRLPAGHVLTQVQLVGTTKKN-----VKVGGQKLNFYTGKLD  305
                                                                         ++                  E              L                           VLT    ++                                                                              +                                  +               +                                +                                 +V                                                          NF       TG+L
Sbjct  441  KDYYMLSWQNRTMCLDELDSLNKSDVLTGLRFKLESVNDLTYLKLEVRLTPYNFTTGRLS  500

Query  306  K  306

Sbjct  501  D  501
```

>**ref|XP_002044470.1|** G GM11959 [Drosophila sechellia]
gb|EDW53677.1| G GM11959 [Drosophila sechellia]
Length=799

GENE ID: 6620262 Dsec\GM11959 | GM11959 gene product from transcript GM11959-RA [Drosophila sechellia] (10 or fewer PubMed links)

Score = 43.9 bits (102), Expect = 0.035
Identities = 28/164 (18%), Positives = 54/164 (33%), Gaps = 14/164 (8%)

```
Query  180  DGEVVRSCKQNNVAVGASCMYAGDLKAGANEVITGMRLTKVGNAIWPEIQVAKVTLFGL  238
            D  V          + ++       D+         + V+TG+R    VG    +    E              +
Sbjct  619  DRHVKNGVDYHKLSYEYRTIDLDDVDTEDNSFVVTGVRFRVVGTHLNLEA------YYSE  672

Query  239  IDKSTRKWVGPGGKMKSLCAPIKSLVAAEPTRLPAGHVLTQVQLVGTTKKNVKVGGQKLN  298
             D  T K + P                 +     A     +L      +             +    V +           Q ++
Sbjct  673  FDFLTGQLIQPEYNSYWKSNDNTDVSGARREKLSLNADVSTRTIAPSLP-LSRHNQFID  731

Query  299  FYTGKLDKPKTAPAKKVVKLNDLVSVDIEPVYVSTNVAGAGLRY  342
            F     LDK            + + D+VS       P         +AG G+ Y
Sbjct  732  FTNTGLDKDAAQSTVPFIDIQDVVSNPPVP------LAGIGIYY  769
```

>**gb|ACM40897.1|** putative cysteine endopeptidase [Culicoides nubeculosus]
Length=395

Score = 43.5 bits (101), Expect = 0.045
Identities = 23/129 (17%), Positives = 45/129 (35%), Gaps = 11/129 (8%)

```
Query  187  CKQNNVAVGASCMYAGDLKAGA--NEVITGMRLTKVGNAIWPEIQVAKVTLFGLIDKSTR  244
            C +  N          +           L++            N+VITG+++   +     +  +I+    +   +    G   K+
Sbjct  169  CDEPNDDRSVRSFSLNSLESDTLQNKVITGVQIVQNNVVFCLKIHQSCLYANGKINKNGG  228

Query  245  KWVGPGGKMKSLCAPIK--------SLVAAEPTRLPAGHVLTQVQLVGTTKKNVKVGG  295
                  W                                                                                      S+++                                                 +V+T                       V+                                          +                V
Sbjct  229  SWKSDNPNNNTFNLAFNKHNIHMGSSILSTADPKKTREYVVTGVKFADIEGNLALGVKFT  288

Query  296  KLNFYTGKL  304
                            TGKL
Sbjct  289  LFEISTGKL  297
```

>**gb|ADJ67274.1|** Cul n 10 allergen [Culicoides nubeculosus]
Length=419

Score = 42.8 bits (99), Expect = 0.077
Identities = 21/122 (17%), Positives = 45/122 (36%), Gaps = 8/122 (6%)

```
Query  191  NVAVGASCMYAGDLKAGANEVITGMRLTKVGNAIWPEIQVAKVTLFGLIDKSTRKWVGP-  249
            + +            +              +   A           N+V+      G++L               +     ++I   +    +      G    D+           W
Sbjct  194  SESIRSFSLNAVESDTKDNKVVIGVQLVKDKIFYFKIRQSPLLPLGKADQKRKLSWKSD  253

Query  250  ------GGKMKSLCAPIKSLVAAEPTRLPAGHVLTQVQLVGTTKKNVKVGGQKLNFYTG  302
                                                                   +                                L                                                             +S+                                                        T                                                           V+T                           V+                                                                                                   +                  V                                  K                     +G
Sbjct  254  PSLSSGSKDIFKLDVNKRSIHIGSATSNLTDFVVTGVKFAEIEGNLALGVKLTKFDVMSG  313

Query  303  KL  304
            KL
Sbjct  314  KL  315
```

>**ref|XP_001601809.1|** GM PREDICTED: similar to GA15301-PA [Nasonia vitripennis]
Length=666

GENE ID: 100117628 LOC100117628 | similar to GA15301-PA [Nasonia vitripennis] (10 or fewer PubMed links)

Sort alignments for this subject
E value **Score** **Percent identi**
Query start position **Subject**

Score = 42.4 bits (98), Expect = 0.10
Identities = 15/55 (28%), Positives = 22/55 (40%), Gaps = 1/55 (1%)

```
Query  207  GANEVITGMRLTKVGNAIWPEIQVAKVTLFGLIDKSTRKWVGPGGKMKSLCAPIK  261
                N+V+TG+RL K       +IQ     G  I    +W                  S        K
Sbjct  419  DQNKVVTGVRLIRKHHNHIFHIQIQQGTLEANGTI-SGTLEWKEIKEISSSDLLSDK  472
```

Score = 38.5 bits (88), Expect = 1.5
Identities = 31/150 (21%), Positives = 51/150 (34%), Gaps = 21/150 (14%)

```
Query  192  VAVGASCMYAGDLKAGANEVITGMRLTKVGNAIWPEIQVAKVT-LFGLIDKSTRKWVGPG  250
            V          + +Y      DL +G N ++TG+R    +V  +    EIQ                    +            +                  W
Sbjct  509  VTYDQNTVYLDDLDSGPNALLTGLRFQQVNDDLRLEIQSTPFDYTTTKLKTDQSSW----  564

Query  251  GKMKSLCAPIKSLVAAEPTRLPAGHVLTQVQLVGTTKKNVKVGGQKLNFYTGKLDKPKTA  310
                        +    P                           V                                                                       P                                                           +                                       K+                                                                                                           V+                                                                                                                                              L                                                                   G+                                   P
Sbjct  565  -TANTNTRPTRHEVFLTDPDDPTR--TKESDNFSKNKQFVRFRMTDLRHDLGQYTVP---  618

Query  311  PAKKVVKLNDLVSVDIEPVYVSTNVAGAGL  340
                                                      + L D V+                                                                                                                          P                                                                            ++GAGL
Sbjct  619  ----FLDLQDVTPAALP------ISGAGL  638
```

>**gb|ACE75299.1|** conserved hypothetical protein [Glyptapanteles indiensis]
Length=643

Score = 42.0 bits (97), Expect = 0.13
Identities = 22/133 (17%), Positives = 43/133 (33%), Gaps = 28/133 (21%)

```
Query  209  NEVITGMRLTKVGNAIWPEIQVAKVTLFGLIDKSTRKWVGPG-----------------  250
            N V+TG+R                                       +I+    ++   FG       I                      T              W          G
Sbjct  380  NMVMTGVRFAEKDRMFHLQIEQGELGSFGEIVPGTVEWKELGDFQYDQSAGEGSFSIKKGE  439

Query  251  -------GKMKSLCAPIKSLVAAEPTRLPAGHVLTQVQLVGTTKKN---VKVGGQKLNFY  300
                                                                                               S                                 A                 +                             +                                                               G                                     VL                                                V                                                          +                                                             +                                                              +V              V
Sbjct  440  KFVKLTEFVDFSFVALDQRTINLDELFANDGQVLIGVRFIYNGMDDAFELQVKSWPVDIR  499

Query  301  TGKLDKPKTAPAK  313
            TG+L                                                                                                       +                                                    +
Sbjct  500  TGELANGNPSDVE  512
```

>**gb|ACH56924.1|** hypothetical protein [Simulium vittatum]
Length=389

Score = 40.5 bits (93), Expect = 0.38
Identities = 27/154 (18%), Positives = 62/154 (41%), Gaps = 11/154 (7%)

```
Query  163  DRLARGLNRVAENRFGND-GEVVRSCKQNNVAVGASCMYAGDLKAGANEVITGMRLTKVG  221
            + +     ++ +R+                F           D                  G                                                          K+                         +S                                                                               +                                   +VI                 G+                  LT   K+
Sbjct  125  EVIQMNIHRLSSKFFVCDQGRSGEHYKKFKNSLPMVSLKPQESDIKDGKVIVGLQLTTKD  184

Query  222  NAIWPEIQVAKVTLFGLIDKSTRKWVG-------PGGKMKSLCAPIKSLVAAEPTRLPAG  274
                      I                                              +    +         ++S      W+                                                                                            P                     +                                                                      P                                               +              +         +V+T                                                                     G
Sbjct  185  DVLFYKIKQAPLLPHFHVNESASSWMDIEYMLSRPDDKNLTTVEPYHWKLMMKELTVPEN  244

Query  275  HVLTQVQLVGTTKKNVKVGGQ--KLNFYTGKLD  305
                    VL                     T                                +     +K                                                                                 +              +
Sbjct  245  TVLTGIGFGYDSKNQLDIQLKYTPVLNASSGELD  278
```

>**ref|NP_001138187.1|** UG protein tyrosine phosphatase 10D, isoform E [Drosophila melanog
gb|ACL82919.1| G protein tyrosine phosphatase 10D, isoform E [Drosophila melanogaster]
Length=1558

GENE ID: 32115 Ptp10D | Protein tyrosine phosphatase 10D [Drosophila melanogaster] (Over 10 PubMed links)

Score = 38.2 bits (87), Expect = 1.9
Identities = 20/111 (19%), Positives = 34/111 (31%), Gaps = 7/111 (6%)

```
Query  179  NDGEVVRSCKQNNVAVGASCMYAGDLKAGANEVITGMRLTKVGNAIWPE----IQVAKVT  234
             + ++         K         +     +     G              EV T +                  V N I                     I +
Sbjct  635  TEADITDMTKGEKYTIQVNTVSFGVESPVPQEVNTTVPPNPVSNIIQLVDSRNITLEWPK  694

Query  235  LFGLIDKSTRKWVG---PGGKMKSLCAPIKSLVAAEPTRLPAGHVLTQVQL  282
                G ++         KW                  PG                            + KS                                  R+      G ++      VQ
Sbjct  695  PEGRVESYILKWWPSDNPGRVQTKNVSENKSADDLSTVRVLIGELMPGVQY  745
```

>**ref|XP_001355649.2|** G GA14821 [Drosophila pseudoobscura pseudoobscura]
gb|EAL32708.2| GA14821 [Drosophila pseudoobscura pseudoobscura]
Length=1955

GENE ID: 4815782 Dpse\GA14821 | GA14821 gene product from transcript GA14821-RA [Drosophila pseudoobscura pseudoobscura] (10 or fewer PubMed links)

Score = 38.2 bits (87), Expect = 1.9
Identities = 20/111 (19%), Positives = 34/111 (31%), Gaps = 7/111 (6%)

```
Query  179  NDGEVVRSCKQNNVAVGASCMYAGDLKAGANEVITGMRLTKVGNAIWPE----IQVAKVT  234
             + ++         K         +     +     G              EV T +                  V N I                     I +
Sbjct  608  TEADITDMTKGEKYTIQVNTVSFGVESPVPQEVNTTVPPNPVSNIIQLVDSRNITLEWPK  667

Query  235  LFGLIDKSTRKWVG---PGGKMKSLCAPIKSLVAAEPTRLPAGHVLTQVQL  282
                G ++         KW                  PG                            + KS                                  R+      G ++      VQ
Sbjct  668  PEGRVESYILKWWPSDNPGRVQTKNVSENKSADDLSTVRVLIGELMPGVQY  718
```

>**ref|XP_002106698.1|** UG Ptp10D [Drosophila simulans]
gb|EDX17660.1| G Ptp10D [Drosophila simulans]
Length=1316

GENE ID: 6725695 Dsim\Ptp10D | GD17038 gene product from transcript GD17038-RA [Drosophila simulans] (10 or fewer PubMed links)

Score = 38.2 bits (87), Expect = 1.9
Identities = 20/111 (19%), Positives = 34/111 (31%), Gaps = 7/111 (6%)

```
Query  179  NDGEVVRSCKQNNVAVGASCMYAGDLKAGANEVITGMRLTKVGNAIWPE----IQVAKVT  234
             + ++         K         +     +     G              EV T +                  V N I                     I +
Sbjct  598  TEADITDMTKGEKYTIQVNTVSFGVESPVPQEVNTTVPPNPVSNIIQLVDSRNITLEWPK  657

Query  235  LFGLIDKSTRKWVG---PGGKMKSLCAPIKSLVAAEPTRLPAGHVLTQVQL  282
                G ++         KW                  PG                            + KS                                  R+      G ++      VQ
Sbjct  658  PEGRVESYILKWWPSDNPGRVQTKNVSENKSADDLSTVRVLIGELMPGVQY  708
```

>**ref|XP_002101393.1|** G GE15658 [Drosophila yakuba]
gb|EDX02501.1| G GE15658 [Drosophila yakuba]
Length=1970

GENE ID: 6525558 Dyak\GE15658 | GE15658 gene product from transcript GE15658-RA [Drosophila yakuba] (10 or fewer PubMed links)

Score = 38.2 bits (87), Expect = 1.9
Identities = 20/111 (19%), Positives = 34/111 (31%), Gaps = 7/111 (6%)

```
Query  179  NDGEVVRSCKQNNVAVGASCMYAGDLKAGANEVITGMRLTKVGNAIWPE----IQVAKVT  234
             + ++         K         +     +     G              EV T +                  V N I                     I +
Sbjct  635  TEADITDMTKGEKYTIQVNTVSFGVESPVPQEVNTTVPPNPVSNIIQLVDSRNITLEWPK  694

Query  235  LFGLIDKSTRKWVG---PGGKMKSLCAPIKSLVAAEPTRLPAGHVLTQVQL  282
                G ++         KW                  PG                            + KS                                  R+      G ++      VQ
Sbjct  695  PEGRVESYILKWWPSDNPGRVQTKNVSENKSADDLSTVRVLIGELMPGVQY  745
```

>**ref|XP_002044105.1|** G GM13099 [Drosophila sechellia]
gb|EDW51417.1| G GM13099 [Drosophila sechellia]
Length=1977

GENE ID: 6619893 Dsec\GM13099 | GM13099 gene product from transcript GM13099-RA [Drosophila sechellia] (10 or fewer PubMed links)

Score = 38.2 bits (87), Expect = 1.9
Identities = 20/111 (19%), Positives = 34/111 (31%), Gaps = 7/111 (6%)

```
Query  179  NDGEVVRSCKQNNVAVGASCMYAGDLKAGANEVITGMRLTKVGNAIWPE----IQVAKVT  234
             + ++         K         +     +     G              EV T +                  V N I                     I +
Sbjct  636  TEADITDMTKGEKYTIQVNTVSFGVESPVPQEVNTTVPPNPVSNIIQLVDSRNITLEWPK  695

Query  235  LFGLIDKSTRKWVG---PGGKMKSLCAPIKSLVAAEPTRLPAGHVLTQVQL  282
                G ++         KW                  PG                            + KS                                  R+      G ++      VQ
Sbjct  696  PEGRVESYILKWWPSDNPGRVQTKNVSENKSADDLSTVRVLIGELMPGVQY  746
```

>**ref|XP_002022578.1|** G GL13109 [Drosophila persimilis]
gb|EDW26613.1| G GL13109 [Drosophila persimilis]
Length=476

GENE ID: 6597375 Dper\GL13109 | GL13109 gene product from transcript GL13109-RA [Drosophila persimilis] (10 or fewer PubMed links)

Score = 38.2 bits (87), Expect = 1.9
Identities = 20/111 (19%), Positives = 34/111 (31%), Gaps = 7/111 (6%)

```
Query  179  NDGEVVRSCKQNNVAVGASCMYAGDLKAGANEVITGMRLTKVGNAIWPE----IQVAKVT  234
             + ++         K         +     +     G              EV T +                  V N I                     I +
Sbjct  220  TEADITDMTKGEKYTIQVNTVSFGVESPVPQEVNTTVPPNPVSNIIQLVDSRNITLEWPK  279

Query  235  LFGLIDKSTRKWVG---PGGKMKSLCAPIKSLVAAEPTRLPAGHVLTQVQL  282
                G ++         KW                  PG                            + KS                                  R+      G ++      VQ
Sbjct  280  PEGRVESYILKWWPSDNPGRVQTKNVSENKSADDLSTVRVLIGELMPGVQY  330
```

>**ref|XP_001946656.1|** GM PREDICTED: similar to 120.7 kDa protein in NOF-FB transposable element, partial [Acyrthosiphon pisum]
Length=1538

GENE ID: 100161269 LOC100161269 | similar to 120.7 kDa protein in NOF-FB transposable element [Acyrthosiphon pisum]

Score = 38.2 bits (87), Expect = 1.9
Identities = 20/100 (20%), Positives = 42/100 (42%), Gaps = 0/100 (0%)

```
Query  125  VTIPKMQREHELVMGFLLNRLRIFALRAKKCTDNWESKDRLARGLNRVAENRFGNDGEVV  184
             + IP ++ E + V+     + L     F+++                  D          +D+                  NR ++ R   R
Sbjct  1435 IVIPDLENEEDEVIMSNHSSLNTFSVKNNIIPDTVYDEDKATENWNRKSKRRIKSYLTTN  1494

Query  185  RSCKQNNVAVGASCMYAGDLKAGANEVITGMRLTKVGNAI  224
                    K     +++            SC                LK G+   E     +++         G    I
Sbjct  1495 PHLKYLDLSSKKSCRSLPVLKNGSIEDLKAIQIRGYGKII  1534
```

>**ref|XP_001977094.1|** G GG18436 [Drosophila erecta]
gb|EDV46021.1| G GG18436 [Drosophila erecta]
Length=1978

GENE ID: 6551306 Dere\GG18436 | GG18436 gene product from transcript GG18436-RA [Drosophila erecta] (10 or fewer PubMed links)

Score = 38.2 bits (87), Expect = 1.9
Identities = 20/111 (19%), Positives = 34/111 (31%), Gaps = 7/111 (6%)

```
Query  179  NDGEVVRSCKQNNVAVGASCMYAGDLKAGANEVITGMRLTKVGNAIWPE----IQVAKVT  234
             + ++         K         +     +     G              EV T +                  V N I                     I +
Sbjct  635  TEADITDMTKGEKYTIQVNTVSFGVESPVPQEVNTTVPPNPVSNIIQLVDSRNITLEWPK  694

Query  235  LFGLIDKSTRKWVG---PGGKMKSLCAPIKSLVAAEPTRLPAGHVLTQVQL  282
                G ++         KW                  PG                            + KS                                  R+      G ++      VQ
Sbjct  695  PEGRVESYILKWWPSDNPGRVQTKNVSENKSADDLSTVRVLIGELMPGVQY  745
```

>**ref|XP_001966042.1|** G GF19443 [Drosophila ananassae]
gb|EDV38451.1| G GF19443 [Drosophila ananassae]
Length=1997

GENE ID: 6502202 Dana\GF19443 | GF19443 gene product from transcript GF19443-RA [Drosophila ananassae] (10 or fewer PubMed links)

Score = 38.2 bits (87), Expect = 1.9
Identities = 20/111 (19%), Positives = 34/111 (31%), Gaps = 7/111 (6%)

```
Query  179  NDGEVVRSCKQNNVAVGASCMYAGDLKAGANEVITGMRLTKVGNAIWPE----IQVAKVT  234
             + ++         K         +     +     G              EV T +                  V N I                     I +
Sbjct  669  TEADITDMTKGEKYTIQVNTVSFGVESPVPQEVNTTVPPNPVSNIIQLVDSRNITLEWPK  728

Query  235  LFGLIDKSTRKWVG---PGGKMKSLCAPIKSLVAAEPTRLPAGHVLTQVQL  282
                G ++         KW                  PG                            + KS                                  R+      G ++      VQ
Sbjct  729  PEGRVESYILKWWPSDNPGRVQTKNVSENKSADDLSTVRVLIGELMPGVQY  779
```

>**gb|AAO42638.1|** RE52018p [Drosophila melanogaster]
Length=1631

Score = 38.2 bits (87), Expect = 1.9
Identities = 20/111 (19%), Positives = 34/111 (31%), Gaps = 7/111 (6%)

```
Query  179  NDGEVVRSCKQNNVAVGASCMYAGDLKAGANEVITGMRLTKVGNAIWPE----IQVAKVT  234
             + ++         K         +     +     G              EV T +                  V N I                     I +
Sbjct  635  TEADITDMTKGEKYTIQVNTVSFGVESPVPQEVNTTVPPNPVSNIIQLVDSRNITLEWPK  694

Query  235  LFGLIDKSTRKWVG---PGGKMKSLCAPIKSLVAAEPTRLPAGHVLTQVQL  282
                G ++         KW                  PG                            + KS                                  R+      G ++      VQ
Sbjct  695  PEGRVESYILKWWPSDNPGRVQTKNVSENKSADDLSTVRVLIGELMPGVQY  745
```

>**gb|AAA28952.1|** receptor-linked protein tyrosine phosphatase [Drosophila melanogaster]
Length=1558

Score = 38.2 bits (87), Expect = 1.9
Identities = 20/111 (19%), Positives = 34/111 (31%), Gaps = 7/111 (6%)

```
Query  179  NDGEVVRSCKQNNVAVGASCMYAGDLKAGANEVITGMRLTKVGNAIWPE----IQVAKVT  234
             + ++         K         +     +     G              EV T +                  V N I                     I +
Sbjct  635  TEADITDMTKGEKYTIQVNTVSFGVESPVPQEVNTTVPPNPVSNIIQLVDSRNITLEWPK  694

Query  235  LFGLIDKSTRKWVG---PGGKMKSLCAPIKSLVAAEPTRLPAGHVLTQVQL  282
                G ++         KW                  PG                            + KS                                  R+      G ++      VQ
Sbjct  695  PEGRVESYILKWWPSDNPGRVQTKNVSENKSADDLSTVRVLIGELMPGVQY  745
```

>**gb|AAA28484.1|** protein tyrosine phosphatase [Drosophila melanogaster]
Length=1631

Score = 38.2 bits (87), Expect = 1.9
Identities = 20/111 (19%), Positives = 34/111 (31%), Gaps = 7/111 (6%)

```
Query  179  NDGEVVRSCKQNNVAVGASCMYAGDLKAGANEVITGMRLTKVGNAIWPE----IQVAKVT  234
             + ++         K         +     +     G              EV T +                  V N I                     I +
Sbjct  635  TEADITDMTKGEKYTIQVNTVSFGVESPVPQEVNTTVPPNPVSNIIQLVDSRNITLEWPK  694

Query  235  LFGLIDKSTRKWVG---PGGKMKSLCAPIKSLVAAEPTRLPAGHVLTQVQL  282
                G ++         KW                  PG                            + KS                                  R+      G ++      VQ
Sbjct  695  PEGRVESYILKWWPSDNPGRVQTKNVSENKSADDLSTVRVLIGELMPGVQY  745
```

>**ref|NP_996413.1|** UG protein tyrosine phosphatase 10D, isoform D [Drosophila melanogast
ref|NP_996414.1| G protein tyrosine phosphatase 10D, isoform A [Drosophila melanogaster
gb|AAS65319.1| G protein tyrosine phosphatase 10D, isoform A [Drosophila melanogaster]
gb|AAS65320.1| G protein tyrosine phosphatase 10D, isoform D [Drosophila melanogaster]
Length=1962

GENE ID: 32115 Ptp10D | Protein tyrosine phosphatase 10D [Drosophila melanogaster] (Over 10 PubMed links)

Score = 38.2 bits (87), Expect = 1.9
Identities = 20/111 (19%), Positives = 34/111 (31%), Gaps = 7/111 (6%)

```
Query  179  NDGEVVRSCKQNNVAVGASCMYAGDLKAGANEVITGMRLTKVGNAIWPE----IQVAKVT  234
             + ++         K         +     +     G              EV T +                  V N I                     I +
Sbjct  635  TEADITDMTKGEKYTIQVNTVSFGVESPVPQEVNTTVPPNPVSNIIQLVDSRNITLEWPK  694

Query  235  LFGLIDKSTRKWVG---PGGKMKSLCAPIKSLVAAEPTRLPAGHVLTQVQL  282
                G ++         KW                  PG                            + KS                                  R+      G ++      VQ
Sbjct  695  PEGRVESYILKWWPSDNPGRVQTKNVSENKSADDLSTVRVLIGELMPGVQY  745
```

>**ref|NP_727544.2|** G protein tyrosine phosphatase 10D, isoform B [Drosophila melanogaster
sp|P35992.3|PTP10_DROME G RecName: Full=Tyrosine-protein phosphatase 10D; AltName: Full protein-tyrosine phosphatase 10D; Short=DPTP10D;
Flags: Precursor
gb|AAF48072.3| G protein tyrosine phosphatase 10D, isoform B [Drosophila melanogaster]
Length=1631

GENE ID: 32115 Ptp10D | Protein tyrosine phosphatase 10D [Drosophila melanogaster] (Over 10 PubMed links)

Score = 38.2 bits (87), Expect = 1.9
Identities = 20/111 (19%), Positives = 34/111 (31%), Gaps = 7/111 (6%)

```
Query  179  NDGEVVRSCKQNNVAVGASCMYAGDLKAGANEVITGMRLTKVGNAIWPE----IQVAKVT  234
             + ++         K         +     +     G              EV T +                  V N I                     I +
Sbjct  635  TEADITDMTKGEKYTIQVNTVSFGVESPVPQEVNTTVPPNPVSNIIQLVDSRNITLEWPK  694

Query  235  LFGLIDKSTRKWVG---PGGKMKSLCAPIKSLVAAEPTRLPAGHVLTQVQL  282
                G ++         KW                  PG                            + KS                                  R+      G ++      VQ
Sbjct  695  PEGRVESYILKWWPSDNPGRVQTKNVSENKSADDLSTVRVLIGELMPGVQY  745
```

>**emb|CBM69274.1|** venom protein Ci-60 [Chelonus inanitus]
Length=205

Score = 37.8 bits (86), Expect = 2.5
Identities = 13/61 (22%), Positives = 23/61 (38%), Gaps = 2/61 (3%)

```
Query  224  IWPEIQVAKVTLFGLIDKSTRKWVGPGGKMKSLCAPIKSLVA--AEPTRLPAGHVLTQVQ  281
              +IQ  K+    G+I + T   WV P             +        +       A         +       P    + LT +
Sbjct  2    VKLQIQKGKLQNGGIIGRGTNHWVPPSRTCTKVAPIVHDATAFVFDNITFPPDYALTGIS  61

Query  282  L  282

Sbjct  62   F  62
```

>**gb|ACH56925.1|** hypothetical protein [Simulium vittatum]
Length=221

Score = 37.8 bits (86), Expect = 2.5
Identities = 22/106 (21%), Positives = 41/106 (39%), Gaps = 8/106 (7%)

```
Query  208  ANEVITGMRLTKVGNAIWPEIQVAKVTLFGLIDKSTRKWVGP-----GGKMKSLCAPIKS  262
            +VI G++L           +          +I    A    +      ++    S +KW                                      K++       P
Sbjct  5    NGKVIVGLQLLAKDGVLTFKILEAPLLPHFHVNASEKKWKELEYIRSSPDNKTVVEPHHW  64

Query  263  LVAAEPTRLPAGHVLTQVQLVGTTKKNVKVGGQ---KLNFYTGKLD  305
                +     +P           VLT   +                     K     +     +          LN        TG+LD
Sbjct  65   KLMMKELTVPENTVLTGIGFRYDGKNQLDIQLKYTPVLNASTGELD  110
```

>**gb|ACZ28340.1|** hypothetical conserved protein [Simulium nigrimanum]
Length=346

Score = 37.4 bits (85), Expect = 3.2
Identities = 21/109 (20%), Positives = 42/109 (39%), Gaps = 14/109 (12%)

```
Query  208  ANEVITGMRLTKVGNAIWPEIQVAKVTLFGLIDKSTRKWVGPGGKMKSLCAPIKSLVA--  265
                +VI G++LT V + +     +I    A    +          ++         +W+                                                                                                                          + V
Sbjct  126  NGKVIVGLQLTIVFDVLTFQIMQAPLISNFYVNATQSEWI---KTYNQHSLANNNTVVEP  182

Query  266  ------AEPTRLPAGHVLTQVQLVGTTKKNVKVGGQ---KLNFYTGKLD  305
                                                    +             +P                  VLT   +                     +K     +     +          LN        TG+LD
Sbjct  183  YHWKLMMKELTVPENTVLTGIGFRYDSKNQLDIQLKYTPVLNATTGELD  231
```

>**dbj|BAE94118.1|** S-layer protein [Clostridium difficile]
Length=341

Score = 37.0 bits (84), Expect = 4.2
Identities = 43/168 (26%), Positives = 68/168 (41%), Gaps = 13/168 (7%)

```
Query  206  AGANEVITGMRLTKVGNAIWPEIQVAKVTLFGLI-DKSTRKWVGPGGKMKSLCAPIKSLV  264
            A +N +    +   KV           +      K++          +I  D S+                      V PGG              +           +K+LV
Sbjct  30   ADSNYTVVQGKYQKVITGLQDALKDGKISKIDVIDGSSIGEVEPGGDPAAAAVKLKNLV  89

Query  265  AAEPTRLPAGHVLTQVQLVGTTKKNVKVGGQKLNFYTGKLDKPKTAPAKK-VVKLNDLVS  323
                    +     L     G                 VQ                                  T                   +L        Y                  KLDK    K                          + +                        +N  L+
Sbjct  90   DDKLDNLGDGKY---VQFNVTYSTTSVITKAELKNYYNKLDKEKILVSNEPTTTVNGLIK  146

Query  324  VDIEPVYVSTNVAGAGLR--------YDVETKTITPVLVAQNMATLKV  363
                    D             P                           +                     A                     L+                                                  YD E                T+T                             A N+A+  KV
Sbjct  147  ADQSPNQANGVAADEPLKLSDVITLSYDDEAGTLTATQNATNVASNKV  194
```

>**ref|YP_003292364.1|** G putative secreted protein [Lactobacillus johnsonii FI9785]
emb|CAX66097.1| G putative secreted protein [Lactobacillus johnsonii FI9785]
Length=953

GENE ID: 8570992 FI9785_210 | putative secreted protein [Lactobacillus johnsonii FI9785] (10 or fewer PubMed links)

Score = 36.6 bits (83), Expect = 5.5
Identities = 22/73 (31%), Positives = 34/73 (47%), Gaps = 0/73 (0%)

```
Query  10   IASISAVANGYLSDLTAAEKMANVINKAAKDDAKKSIRDSKFLNSAMDQLNLEFINPYNT  69
            +A    A A     LS+  TAA K         AA++D +                   + K  + +A+D LN        N
Sbjct  643  VAEAVAQAQNVLSEATAAVKASQAKVDAAQNDVQAKDNNLKSVQAALDSLNQTLNNLENA  702

Query  70   TNNHVGMEEGLAK  82
             +N           +     L K
Sbjct  703  QSNLSAAQTALNK  715
```

>**ref|XP_002040062.1|** G GM15999 [Drosophila sechellia]
gb|EDW56927.1| G GM15999 [Drosophila sechellia]
Length=549

GENE ID: 6615691 Dsec\GM15999 | GM15999 gene product from transcript GM15999-RA [Drosophila sechellia] (10 or fewer PubMed links)

Score = 36.6 bits (83), Expect = 5.5
Identities = 8/58 (14%), Positives = 23/58 (40%), Gaps = 1/58 (1%)

```
Query  179  NDGEVVRSCKQNNVAVGASCMYAGDLKAGA-NEVITGMRLTKVGNAIWPEIQVAKVTL  235
             D +V                   +     + +++   M          ++    ++V+TG R
Sbjct  461  TDVDVRDGYDYHTLSMDSRAMDVDEISVNSTDQVVTGFRFRVFKQHLNLEVLFSSFNF  518
```

>**ref|XP_315969.4|** UG AGAP005939-PA [Anopheles gambiae str. PEST]
gb|EAA11364.4| G AGAP005939-PA [Anopheles gambiae str. PEST]
Length=1055

GENE ID: 1276606 AgaP_AGAP005939 | AGAP005939-PA [Anopheles gambiae str. PEST] (10 or fewer PubMed links)

Score = 36.6 bits (83), Expect = 5.5
Identities = 28/123 (23%), Positives = 47/123 (39%), Gaps = 5/123 (4%)

```
Query  216  RLTKVGNAIWPEIQVAKVTLFGLIDKSTRKWVGPGGKMKSLCAPIKS----LVAAEPTR-  270
            RL           N + P ++         V         D + R+W P                         +     A           +S                         V       +
Sbjct  823  QLLATNNRLQPAVEKTPVKVSPARDTARREWQSPVTPQHTASADTESDQKIKVDLKFLQA  882

Query  271  LPAGHVLTQVQLVGTTKKNVKVGGQKLNFYTGKLDKPKTAPAKKVVKLNDLVSVDIEPV  330
            LP        L     + +          K N V             +     F          L           P ++PAK++                                                 V          V           P
Sbjct  883  LPLHLRLEVEKQIELNKDNMVVTCNEPEFEEPLLVTPDSSPAKELRTPTKGVVVAAAPDV  942

Query  331  VST  333
            ST
Sbjct  943  AST  945
```

>**gb|EFA79656.1|** DNA-dependent protein kinase subunit [Polysphondylium pallidum PN500]
Length=4074

Score = 36.2 bits (82), Expect = 7.2
Identities = 30/130 (24%), Positives = 54/130 (42%), Gaps = 18/130 (13%)

```
Query  101  IRERSDYFGNQLANNRPDDRI--------LCPVTIPKMQREHELVMGFLLNRLRIFALRA  152
            IRER++        +                                           R                         I                                     L                          +          PK                       RE++L                        +                              +        ++I+                        L+A
Sbjct  3188 IRERAELYHQMSKGARKLGNIIVSEAYFRLAVKSYPKT-RENDLAFPLVSSLIKIYCLKA  3246

Query  153  KKCTDNWESKDRLARGLNRVAENRFGNDGEVVRSCKQNNVAVGAS------CMYAGDLKA  206
            K T                                                      E+   DR           +                                   L           V                                                                                +D           +          +++                                                        N                                                                                                                         +Y           D           K
Sbjct  3247 KNSTTQVETLDRFIKALKFVESK---SDEDSIKNNPDNLQRYKRLHGNIQWEIYQLDQKL  3303

Query  207  GANEVITGMR  216
            G+N V+   ++
Sbjct  3304 GSNLVMENLK  3313
```

>**ref|ZP_04797167.1|** D cell wall associated fibronectin-binding protein [Staphylococcus epidermidis W23144]
gb|EES36170.1| D cell wall associated fibronectin-binding protein [Staphylococcus epidermidis W23144]
Length=10153

Score = 36.2 bits (82), Expect = 7.2
Identities = 47/215 (22%), Positives = 79/215 (37%), Gaps = 37/215 (17%)

```
Query  21   LSDLTAA--EKMANVINKA-AKDDAKKSIRDSKFLNSAMDQLNLEFINPYNTTNNHVGME  77
            +L+DLT A    EK+       IN +        ++DD K   I    +K LN AM  QL                                                                 N
Sbjct  6243 LTDLTDAQREKLREQINSSNSRDDIKQEAKAIDAMKQLKEQVAQKDGVHANSDYTN  6302

Query  78   EGLAKKLQSEPTTCDAARTISYTIRERSDYFGNQLANNRPDDRILCPVT-----------  126
            E          A+K                                                                                                              DA                                    ++                      +++                                        D                    N                              N                                                                  I                           +
Sbjct  6303 EDSAQK--------DA---YNHALKQAEDIINNSSNPNLNAQDITNALNNIKQAQDNLHG  6351

Query  127  IPKMQREHELVMGFL--LNRLR-------IFALRAKKCTDNWESKDRLARGLN---RVAE  174
                K+Q++                                          +                     LN                            L                                                                            I                          A+                                                   D                                                        K                                  +                        A+                      L                                       +          E
Sbjct  6352 AQKLQQDKNTTNQAIGNLNHLNQPQKDALIQAINGATSRDQVAEKLKEAQALDEAMKQLE  6411
```

```
Query  175   NRFGNDGEVVRSCKQNNVAVGASCMYAGDLKAGAN  209
             ++   D ++  S    N        +Y   ++A  +
Sbjct  6412  DQVNQDDQISNSSPFINEDSDKQKIYNDKIQAAKD  6446
```

>**ref|ZP_04006822.1|** conserved hypothetical protein [Lactobacillus johnsonii ATCC 33200]
gb|EEJ60332.1| conserved hypothetical protein [Lactobacillus johnsonii ATCC 33200]
Length=935

Score = 35.8 bits (81), Expect = 9.4
Identities = 22/73 (31%), Positives = 34/73 (47%), Gaps = 0/73 (0%)

```
Query  10   IASISAVANGYLSDLTAAEKMANVINKAAKDDAKKSIRDSKFLNSAMDQLNLEFINPYNT  69
            +A   A A   LS+ TAA K +      AA++D +     + K + +A+D LN    N  N
Sbjct  601  VAETVAQAQNVLSEATAAVKASQAKVDAAQNDVQAKDNNLKSVQAALDSLNQTLNNLENA  660

Query  70   TNNHVGMEEGLAK  82
             +N    +  L K
Sbjct  661  QSNLSAAQTALNK  673
```
